# Development of an accurate kinetic model for the central carbon metabolism of *Escherichia coli*

**DOI:** 10.1186/s12934-016-0511-x

**Published:** 2016-06-21

**Authors:** Nusrat Jahan, Kazuhiro Maeda, Yu Matsuoka, Yurie Sugimoto, Hiroyuki Kurata

**Affiliations:** Department of Bioscience and Bioinformatics, Kyushu Institute of Technology, 680-4 Kawazu, Iizuka, Fukuoka, 820-8502 Japan; Frontier Research Academy for Young Researchers, Kyushu Institute of Technology, 1-1 Sensui-cho, Tobata, Kitakyushu, Fukuoka, 804-8550 Japan; Biomedical Informatics R&D Center, Kyushu Institute of Technology, 680-4 Kawazu, Iizuka, Fukuoka, 820-8502 Japan

**Keywords:** Systems biology, Rational design, Dynamic model, Enzyme kinetics, Transcription factor, Signal transduction, Allosteric enzyme

## Abstract

**Background:**

A kinetic model provides insights into the dynamic response of biological systems and predicts how their complex metabolic and gene regulatory networks generate particular functions. Of many biological systems, *Escherichia coli* metabolic pathways have been modeled extensively at the enzymatic and genetic levels, but existing models cannot accurately reproduce experimental behaviors in a batch culture, due to the inadequate estimation of a specific cell growth rate and a large number of unmeasured parameters.

**Results:**

In this study, we developed a detailed kinetic model for the central carbon metabolism of *E. coli* in a batch culture, which includes the glycolytic pathway, tricarboxylic acid cycle, pentose phosphate pathway, Entner-Doudoroff pathway, anaplerotic pathway, glyoxylate shunt, oxidative phosphorylation, phosphotransferase system (Pts), non-Pts and metabolic gene regulations by four protein transcription factors: cAMP receptor, catabolite repressor/activator, pyruvate dehydrogenase complex repressor and isocitrate lyase regulator. The kinetic parameters were estimated by a constrained optimization method on a supercomputer. The model estimated a specific growth rate based on reaction kinetics and accurately reproduced the dynamics of wild-type *E. coli* and multiple genetic mutants in a batch culture.

**Conclusions:**

This model overcame the intrinsic limitations of existing kinetic models in a batch culture, predicted the effects of multilayer regulations (allosteric effectors and gene expression) on central carbon metabolism and proposed rationally designed fast-growing cells based on understandings of molecular processes.

**Electronic supplementary material:**

The online version of this article (doi:10.1186/s12934-016-0511-x) contains supplementary material, which is available to authorized users.

## Background

Systems biology and synthetic biology aim to understand the mechanism by which a biochemical network yields dynamic behaviors in response to environmental stresses or genetic variations and to rationally design and engineer such networks [1–5]. The biochemical network consists of biomolecules such as genes, proteins and metabolites and their interactions that generate a variety of cellular functions. The systems and synthetic biology approach is to construct a biochemical network map, develop mathematical modeling and simulations, experimentally validate the model and predict or rationally design new functions [6]. Mathematical models must reproduce experimental data and phenotypes under different culture and genetic conditions, which leads to computer-aided design (CAD) of cells [7, 8].

Hundreds of the kinetic models from microbes to mammals have been presented and construction of complete models is still underway, as shown in the BioModels database [9, 10] and JWS online [11]. Out of many biological systems, the central carbon metabolism of *Escherichia coli* has been extensively modeled [12] because (1) *E. coli* is widely used to produce useful materials in the industry and has been studied extensively as a model microbe; and (2) central carbon metabolism is the basis of the conserved phenomenon of life and the hub on which nearly all catabolic and biosynthetic processes are built.

Chassagnole et al. [13] developed a dynamic model that links the sugar transport system with the reactions of the glycolysis and pentose phosphate pathways in *E. coli* to simulate the transient data obtained by the fast sampling system. Schmid et al. [14] constructed a dynamic model that combines the behavior of *trp* operon gene expression with the metabolic network of central carbon metabolism to enhance the production of tryptophan. Experimental observations of intracellular metabolite concentrations under transient response conditions were used to estimate the kinetic parameters. Recently, Pontes Freitas Alberton et al. [15] and Peskov et al. [16] built dynamic models based on more than 100 biochemical reactions to predict changes in steady-state flux distributions of gene knockout mutants and simulated a transient response to a short pulse of substrate addition. Khodayari et al. [17] integrated the ensemble modeling formalism with an efficient genetic algorithm-based technique, which satisfied the steady-state experimental flux data for a wild type (WT) and seven mutant strains in a continuous culture. Their model was a large-scale general mass action model with more than 800 variables, but its application was restricted to the steady state with a constant cell growth rate.

In a batch culture, several kinetic models of *E. coli* central metabolism have been built. Since both metabolite concentrations and enzyme concentrations vary over time and with changing intracellular and extracellular conditions, the models must integrate gene regulatory networks and define cell growth functions that involve a number of reactions and gene expressions. Transcription factors (TFs), such as catabolite repressor/activator (Cra), cAMP receptor protein (Crp), anoxic redox control protein (ArcA), pyruvate dehydrogenase complex repressor (PdhR), fumarate nitrate reduction regulator (Fnr) and isocitrate lyase regulator (IclR), regulate the expression of central metabolic genes. Kadir et al. [18] developed a kinetic model of the central carbon metabolism, including glycolysis, the tricarboxylic acid (TCA) cycle, the pentose phosphate pathway, the glyoxylate shunt and anaplerotic pathways, of WT and a few genetic mutants in a batch culture. They incorporated metabolic gene regulations by if–then rule-based Boolean logic, but the model required re-tuning to reproduce the values of critical parameters in the given experimental data for each genetic mutant. Matsuoka et al. [19] built a kinetic model of the central carbon metabolism with xylose and glucose uptake systems, incorporating the dynamics of metabolic enzyme activities without gene expression. Kotte et al. [20] integrated the enzymatic reactions and transcriptional regulations of the central metabolism where multiple transcription factors regulated the metabolic gene expression rates. The Kotte model simulated the dynamics of WT in a batch culture but was not intensively validated by experimental data because the model focused on the regulatory architecture of the metabolic and gene regulatory networks rather than reproducing experimental data. Usuda et al. [21] constructed a kinetic model integrating the phosphotransferase system, glycolysis, the pentose phosphate pathway, the TCA cycle, the glyoxylate shunt, anaplerotic pathways, glutamine/aspartate metabolisms and metabolic gene expressions together with several TFs. This model reproduced a flux distribution and metabolite profile comparable to experimental data in a batch culture, while the specific cell growth rate was given by experimental time-course data of optical densities.

Estimation of specific growth rates is key to dynamic modeling in a batch culture. The Monod equation [22] and its derivatives are widely used to estimate growth rates by assuming a single growth-limiting substrate. To incorporate multiple growth-determining factors, the carbon catabolite repression model used all incoming substrate fluxes, weighted with yield coefficients, to estimate specific growth rates [23, 24], but it is often difficult to estimate the yield coefficients, which depend on environmental conditions and the genetic status of cells. As an alternative approach, the specific growth rate was formulated as a function of ATP production flux, which can be estimated by kinetic models [18, 19].

In this study, we develop a kinetic model for the central carbon metabolism of *E. coli* in a batch culture to overcome intrinsic problems of the existing kinetic models. Our model accurately reproduced the dynamics of WT and multiple genetic mutants in a batch culture and estimated a specific growth rate based on molecular processes. The unknown values of kinetic parameters were estimated by a constrained optimization method on a supercomputer.

## Results

### Model construction

**Fig. 1:**
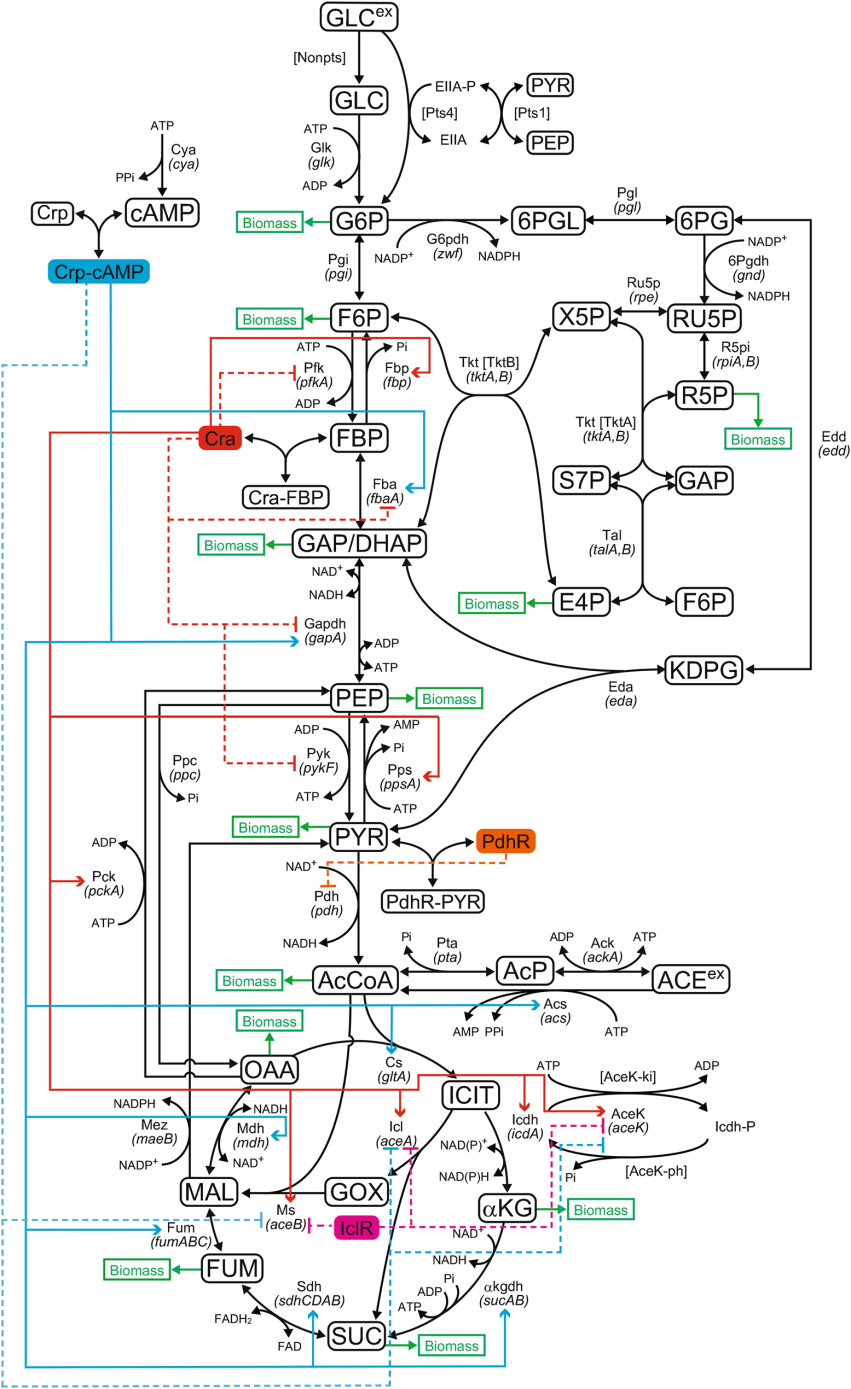
An *E.coli* metabolic network map. The *solid line* indicates activation and the *dotted line* indicates repression

We developed a detailed kinetic model for the central carbon metabolism of *E. coli*, including the glycolytic pathway, TCA cycle, pentose phosphate pathway, Entner-Doudoroff (ED) pathway, anaplerotic pathway, glyoxylate shunt, oxidative phosphorylation, Pts and non-Pts. Four TFs (Crp, Cra, PdhR and IclR) were employed to regulate metabolic gene expressions. The employed network map is shown in Fig. 1. Crp is a TF that regulates many genes involved in energy metabolism and Cra activates expression of genes in the gluconeogenic pathway, glyoxylate shunt and some of TCA cycle and represses expression of genes encoding glycolytic enzymes. The detailed kinetic rate equations were derived from many literatures as shown in "Methods" section. The kinetic model contains 27 metabolites, 22 enzymes and Pts proteins, 38 fluxes, 21 gene expressions and 12 biomass production rate equations for precursor intracellular metabolites. Since the values of many kinetic parameters have not been measured in vivo, we estimated them by a genetic algorithm so that the model could reproduce the experimental data. Experimental data by Shimizu’s group [18, 25], including biomass (cell concentration), extracellular and intracellular metabolite concentrations and metabolic fluxes, were used to construct the kinetic model (Table 1).

**Table 1:** Quantitative representation of the training and testing datasets

		Strain	Extracellular component (Fig. 2)		Intracellular metabolite (Fig. 4)		Intracellular flux (Fig. 5)	
			# time point	# component	# time point	# metabolite	# time point	# flux
Training dataset	Batch culture [25, 28]	WT	12	3	8	9	6	25^a^, 10^b^
		∆*pykF*	10	3	5	9	3	25
		∆*pgi*	18	3	6	9	3	28
		∆*ppc*	23	3	–	–	–	–

		Dilution rate (D)	Intracellular flux (Fig. 6) # flux					
Testing dataset	Continuous culture [26]	0.2	38					
		0.4	38					
		0.5	38					
		0.7	38					

The training datasets were used for model construction. The testing datasets were used for model validation. The numerical values of the experimental data are shown in the MATLAB version of the model: ExpDataForBatchCulture.m, ExpDataForBatchCulture_Flux.m, ExpDataForBatchCulture_IntracellularMetabolite.m, ExpDataForContinuousCulture.m

^a^25 fluxes for three time points (growth phase)

^b^10 fluxes for the remaining three time points (stationary phase)

### Verification by experimental data

**Fig. 2:**
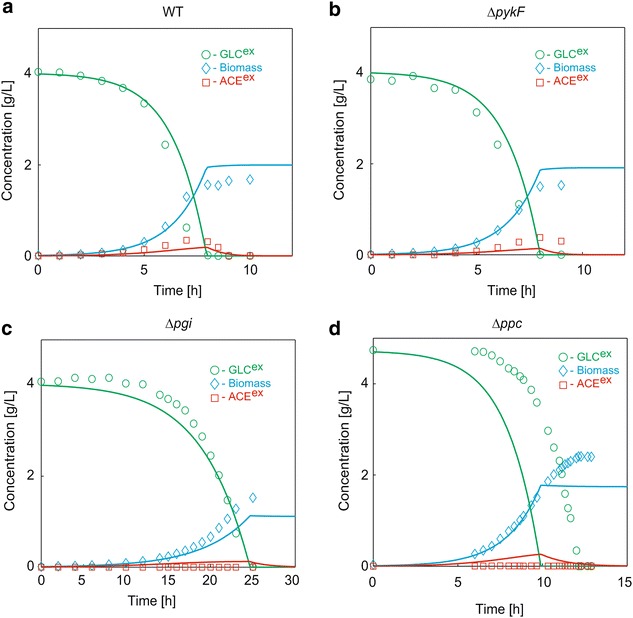
The experimental validation of WT and genetic mutant strains in a batch culture. The *green*, *blue* and *red lines* represent the simulation results of the extracellular glucose, biomass and acetate, respectively. The corresponding open circles represent the experimental data. **a** WT. **b** ∆*pykF*. **c** ∆*pgi*. **d** ∆*ppc*

To test whether the kinetic model can reproduce the experimental data (Table 1), the simulated results of WT, *pykF* knockout mutant (∆*pykF*), *pgi* knockout mutant (∆*pgi*) and *ppc* knockout mutant (∆*ppc*) strains were compared with the experimental data on biomass and extracellular glucose and acetate concentrations in a batch culture (Fig. 2). Pyk catalyzes the conversion of PEP to PYR at the final step of glycolysis, Pgi catalyzes the conversion of G6P to F6P at the initial step of glycolysis and Ppc catalyzes an anaplerotic reaction of PEP to OAA. The time courses for all intracellular metabolite concentrations, enzyme concentrations and metabolic fluxes (enzymatic reaction rates) were simulated as shown in Additional file 1: Figure S1. The biomass concentration increased with a decrease in glucose (Fig. 2). For WT, glucose was completely consumed at approximately 8 h. Acetate was produced during the growth phase and consumed thereafter. The simulated time courses for WT and ∆*pykF* were well consistent with the experimental data, which demonstrates that the kinetic model accurately reproduces the dynamics of not only WT but also a gene-knockout mutant. The growth rate of ∆*pykF* was almost the same as that of WT. Our model reproduced the delay of cell growth and glucose uptake of ∆*pgi* and ∆*ppc*, while there were some discrepancies between the simulated and experimental data. The simulated time-course of extracellular glucose for ∆*pgi* was consistent with experimental data, whereas the simulated cell growth was lower than them, i.e., the model underestimated the cell growth of ∆*pgi*. The simulated time-course of cell growth for ∆*ppc* reproduced the experimental data, whereas the simulated glucose uptake was faster than the experimental data, i.e., the model overestimated the glucose uptake rate.

The model reproduced the experimental acetate production for WT and ∆*pyk*, where the Acs activity responsible for acetate degradation is regulated by Crp-cAMP. When acetate was being consumed as a carbon source, ICIT and GOX transiently increased for WT and ∆*pykF*, showing the activated glyoxylate shunt [25] (Additional file 1: Figure S1). On the other hand, the simulated acetate time-courses for ∆*pgi* and ∆*ppc* were not consistent with experimental data where the acetate production hardly occurred for ∆*pgi* and ∆*ppc*. It suggests that the model misses some mechanisms controlling the acetate production.

**Fig. 3:**
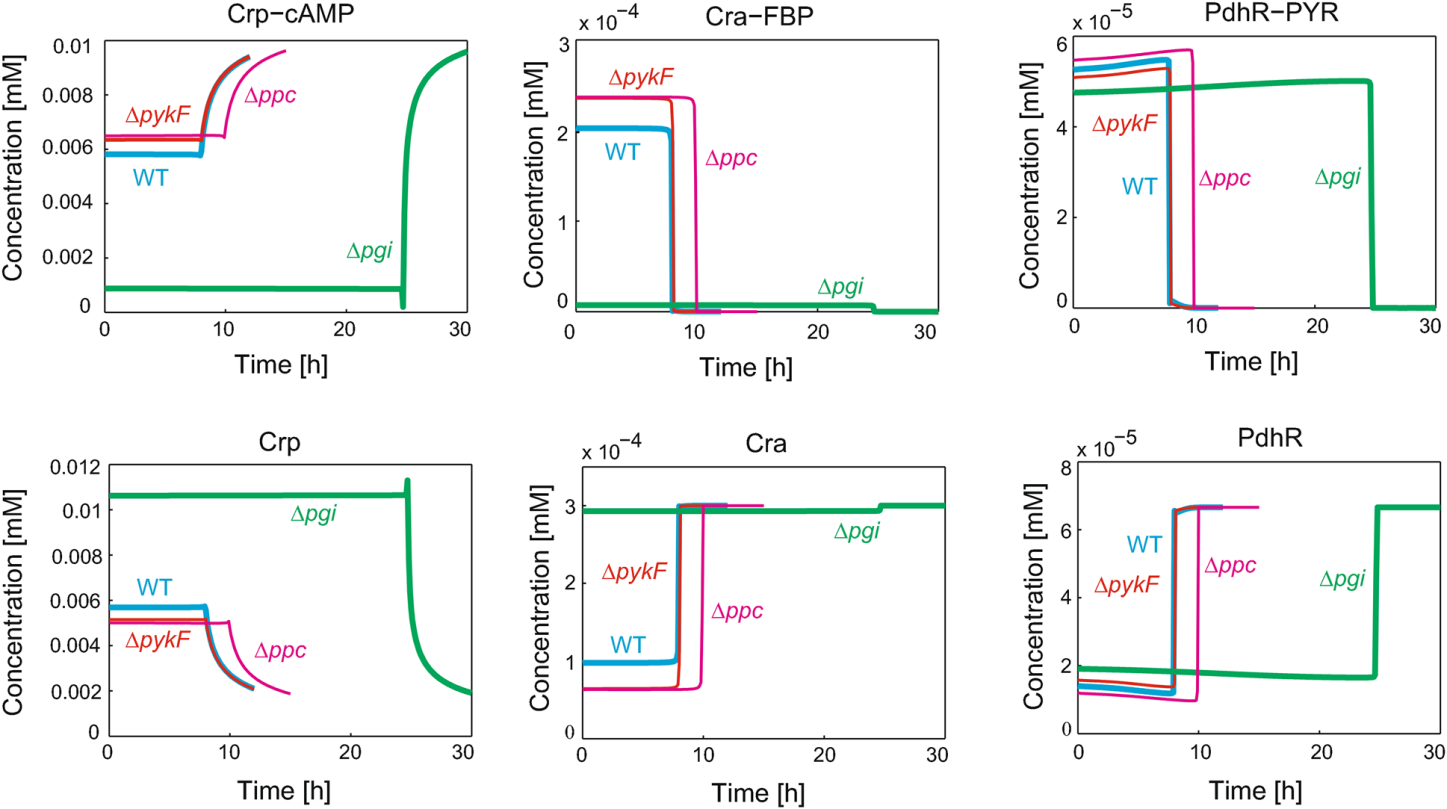
TF and TF-metabolite concentration of WT and genetic mutant strains. The *blue*, *red*, *green* and *magenta lines* indicate WT, ∆*pykF*, ∆*pgi* and ∆*ppc*, respectively

We simulated the time courses of three TFs (Crp, Cra and PdhR) and their complexes for WT and three mutants to confirm their function (Fig. 3). The concentrations of the TFs and their complexes greatly changed between the growth and stationary phases, showing the TFs are directly involved in the phase transition. In the stationary phase, the Crp-cAMP concentration increased with a decrease in the free Crp, the Cra-FBP decreased with an increase in the free Cra and the PdhR-PYR decreased with an increase in the free PdhR. At the beginning of the stationary phase, phosphorylation of EIIA to EIIA-P triggers cAMP production. The resulting Crp-cAMP greatly changed metabolic gene expression.

**Fig. 4:**
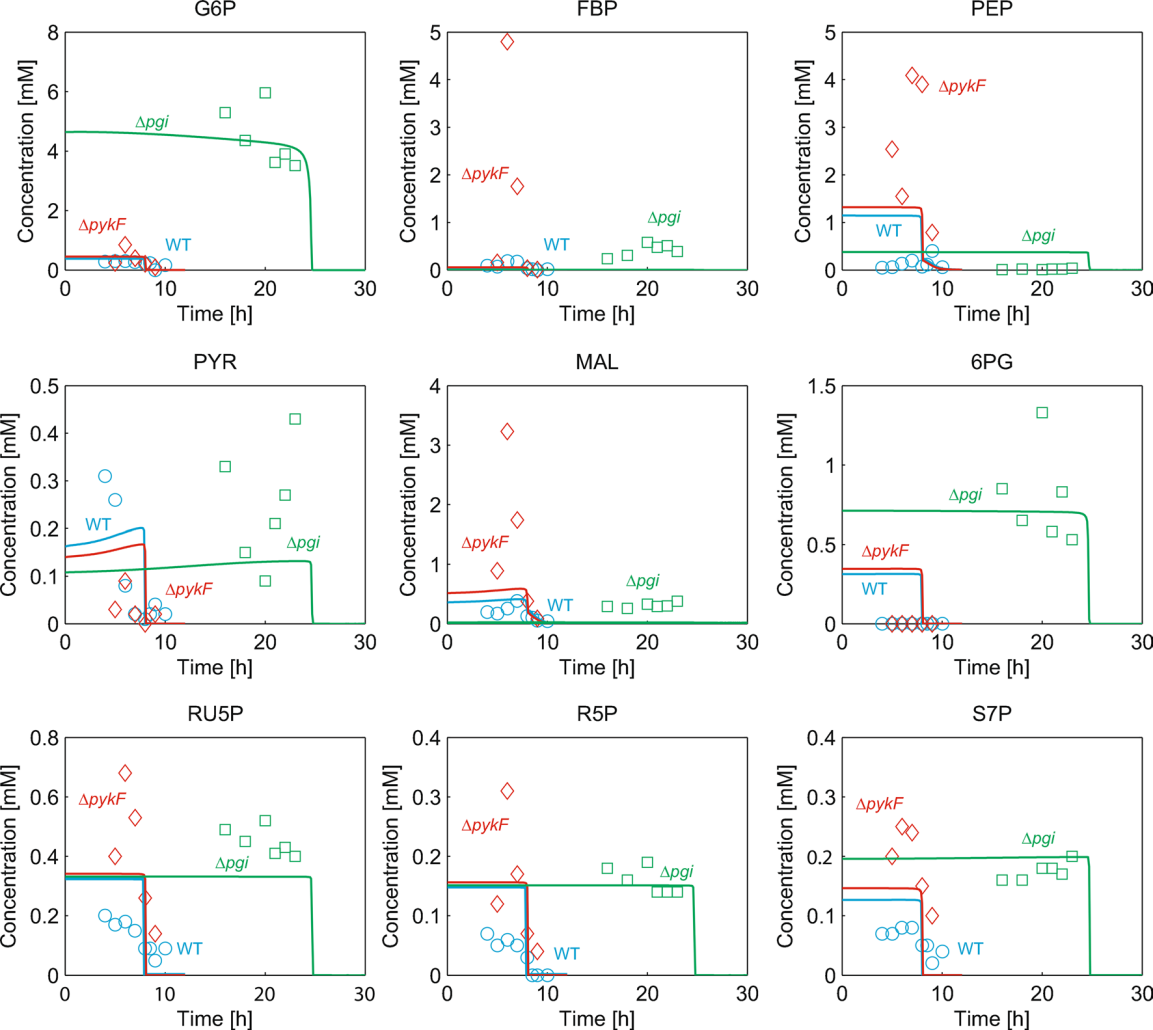
Comparison of the simulated intracellular metabolite concentrations with experimental data for WT and genetic mutant strains in a batch culture. The *blue*, *red* and *green lines* represent the simulation results of WT, ∆*pykF* and ∆*pgi*, respectively. The corresponding *open circles* represent the experimental data

We next tested whether the model reproduces experimental metabolite concentrations for WT, ∆*pykF* and ∆*pgi* (Fig. 4; Additional file 1: Figure S1). Note that there are no experimental data of intracellular metabolite concentrations of ∆*ppc* in a batch culture [18]. ∆*pykF* showed higher experimental concentrations of FBP, PEP, MAL, RU5P, R5P and S7P and a lower concentration of PYR than WT [25]. The simulated results showed the same tendency as the experimental data, while the change in the simulated FBP, PEP, MAL, RU5P, R5P, S7P and PYR between WT and ∆*pykF* was small relative to the experimental data. In the simulation of ∆*pykF*, PEP increased to a small extent, whereas the fluxes of Ppc and Mez increased, which is expected to compensate for a lack of the Pyk flux. The increased PEP increased EIIA-P, thereby increasing cAMP, compared with WT (Additional file 1: Figure S1). An increase in FBP and a decrease in PYR resulted in the increased Cra-FBP and decreased PdhR-PYR, respectively (Additional file 1: Figure S1; Fig. 3). For ∆*pgi*, the simulated metabolite concentrations were rather consistent with experimental data. G6P is metabolized through the pentose phosphate pathway more than the glycolytic pathway. A low flux of Gapdh decreased the Ppc flux, which decreases OAA, resulting in suppression of TCA cycle flux [25]. The low PEP concentration decreased EIIA-P, resulting in a decrease in cAMP and Crp-cAMP, compared with WT (Additional file 1: Figure S1; Fig. 3). A decrease in Crp-cAMP suppressed the *acs* and metabolic genes of the TCA cycle [20]. In addition, the low concentration of PEP decreased the activity of the Pts, which reduced the glucose uptake rate and suppressed cell growth. The decreased FBP and PYR in ∆*pgi* resulted in the decreased Cra-FBP and PdhR-PYR, respectively (Additional file 1: Figure S1; Fig. 3).

**Fig. 5:**
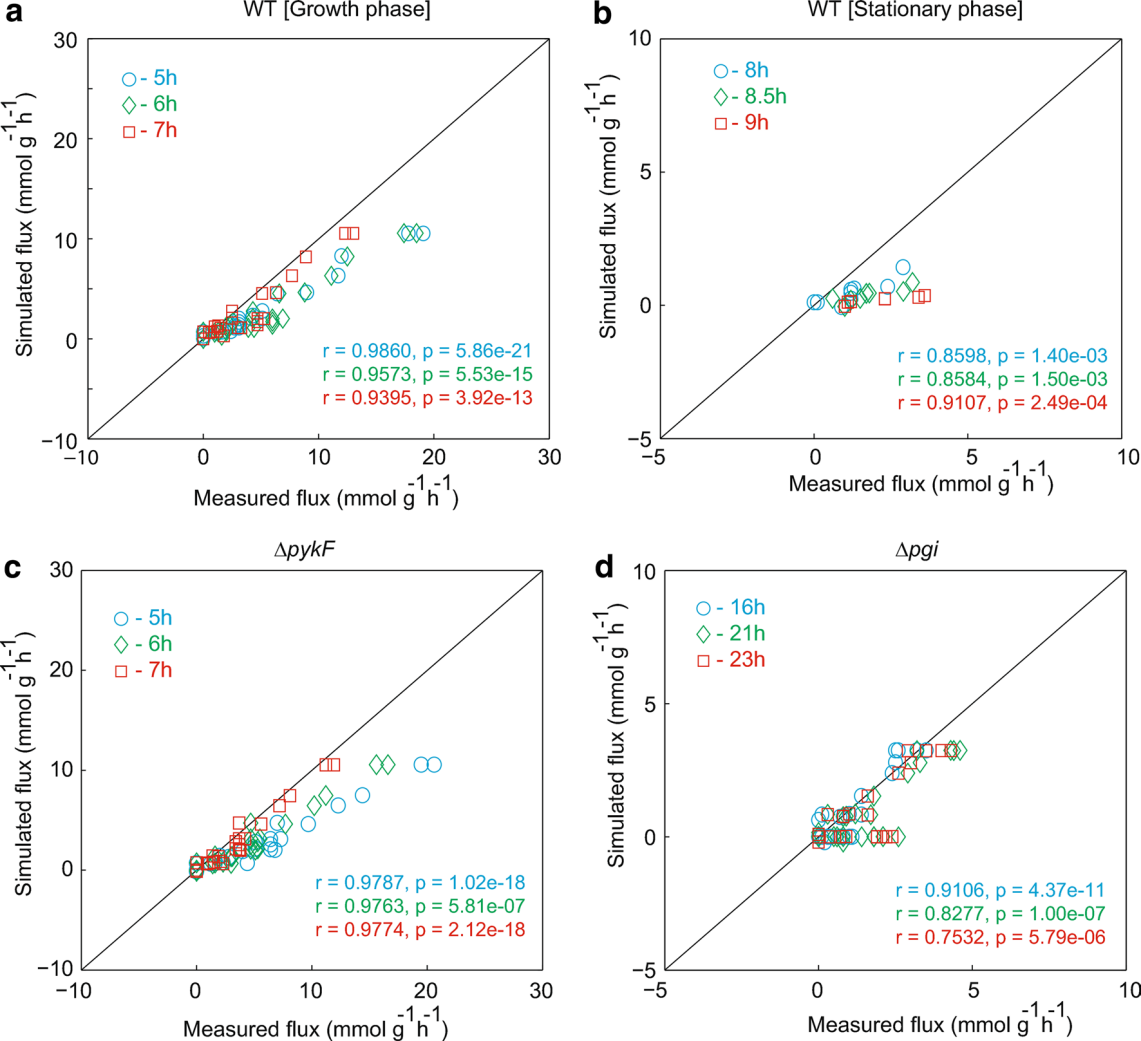
Comparison of the simulated flux with experimental data for WT and genetic mutant strains in a batch culture. **a** Growth phase of WT. The *blue circles*, *green diamonds* and *red squares* indicate data at 5, 6 and 7 h, respectively. **b** Stationary phase of WT. The *blue circles*, *green diamonds* and *red squares* indicate data at 8, 8.5 and 9 h, respectively. **c** ∆*pykF*. The *blue circles*, *green diamonds* and *red squares* indicate data at 5, 6 and 7 h, respectively. **d** ∆*pgi*. The *blue circles*, *green diamonds* and *red squares* indicate data at 16, 21 and 23 h, respectively

To test whether the model reproduces experimental metabolic fluxes, the simulated fluxes of WT were compared with experimental measurements at the growth phase (5, 6 and 7 h) and at the stationary phase (8, 8.5 and 9 h) in a batch culture (Fig. 5a, b) [25]. Note that there are no experimental data of intracellular fluxes of ∆*ppc* in a batch culture [18]. The Pearson’s correlation coefficients (r) between the experimental and simulated fluxes in the growth phase were 0.9860, 0.9573 and 0.9395 with p values of 5.86 × 10^−21^, 5.53 × 10^−15^ and 3.92 × 10^−13^ at 5, 6 and 7 h, respectively. The simulated metabolic fluxes were consistent with the experimental data. In the stationary phase, the Pearson’s correlation coefficients between the experimental and simulated fluxes were 0.8598, 0.8584 and 0.9107 with p values of 1.40 × 10^−3^, 1.50 × 10^−3^ and 2.49 × 10^−4^ at 8, 8.5 and 9 h, respectively. The kinetic model more accurately reproduced the metabolic fluxes of the growth phase than those of the stationary phase. Furthermore, the simulated fluxes of ∆*pykF* were consistent with experimental data [25] at 5, 6 and 7 h in a batch culture with r = 0.9787, 0.9763 and 0.9774 with p values = 1.02 × 10^−18^, 5.81 × 10^−7^ and 2.12 × 10^−18^, respectively (Fig. 5c). As shown in Fig. 5d, the simulated fluxes of ∆*pgi* were rather consistent with experimental data at 16, 21 and 23 h in a batch culture [25]. The Pearson’s correlation coefficients between measured and simulated fluxes for ∆*pgi* were 0.9106, 0.8277 and 0.7532 with p values of 4.37 × 10^−11^, 1.00 × 10^−7^ and 5.79 × 10^−6^ at 16, 21 and 23 h, respectively. We confirmed that the kinetic model underestimates fluxes of the TCA cycle (Additional file 1: Figure S2).

### Validation of the model

**Fig. 6:**
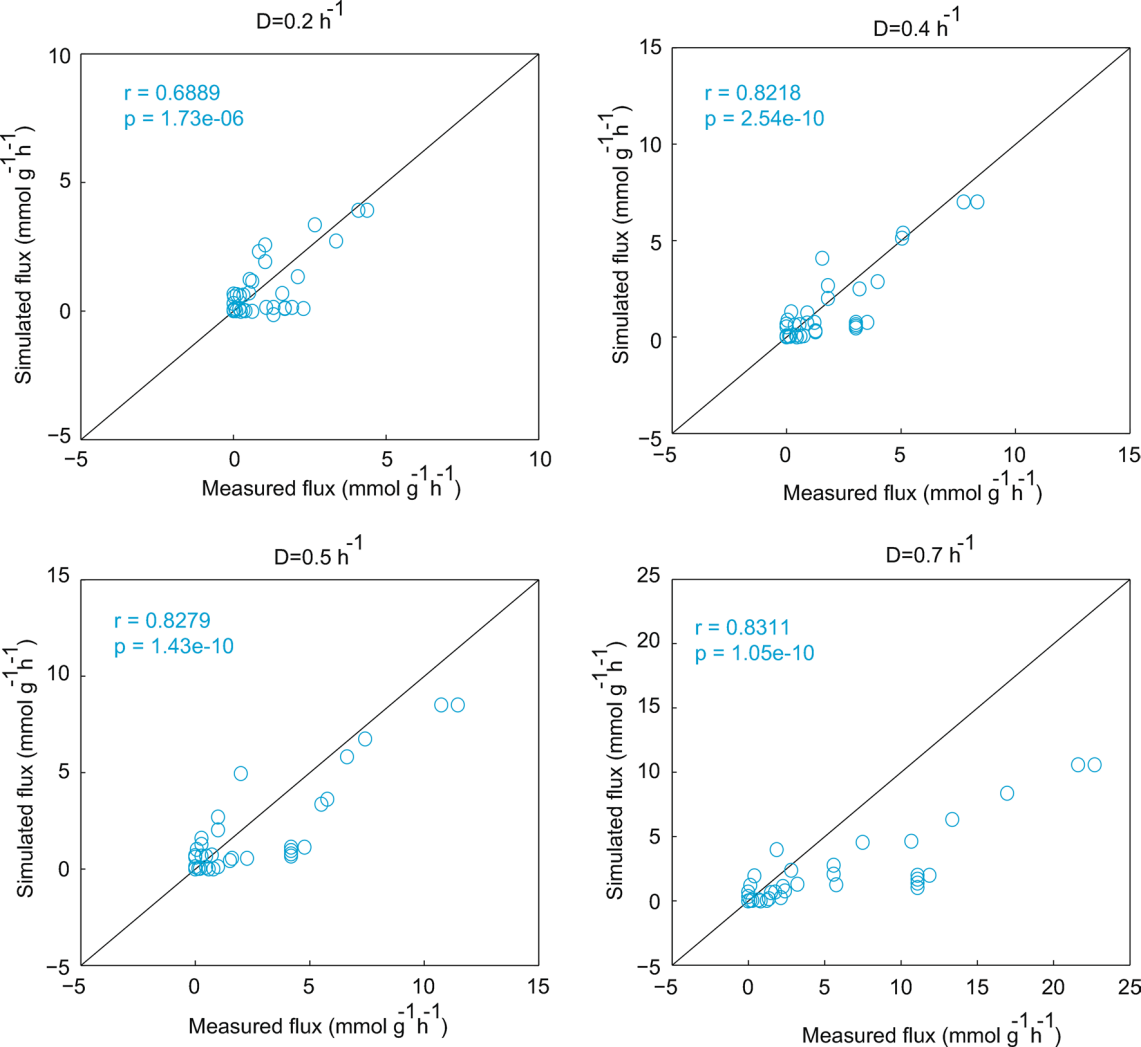
Comparison of the simulated flux with experimental data for WT at different dilution rate (*D*) in a continuous culture

To validate the kinetic model with experimental data that was not used for parameter estimation, we simulated the steady-state flux distribution for a continuous culture [26] (Table 1). We transformed the batch culture model into a continuous culture model by introducing the dilution rate, *D*. Figure 6 compares the predicted fluxes for WT at *D* = 0.2, 0.4, 0.5 and 0.7 h^−1^ with the experimental data, demonstrating the Pearson’s correlation coefficients for WT of 0.6889, 0.8218, 0.8279 and 0.8311, with p values of 1.73 × 10^−6^, 2.54 × 10^−10^, 1.43 × 10^−10^ and 1.05 × 10^−10^, respectively. We confirmed that the kinetic model underestimates fluxes of the TCA cycle (Additional file 1: Figure S3), as seen for the batch culture of ∆*pgi.*

In [26], not only WT but also 24 gene knockout mutants cultured at *D* = 0.2 h^−1^ were available. The kinetic model did not accurately reproduce the steady-state fluxes of those mutants cultured at *D* = 0.2 h^−1^ (data not shown), probably because our model was built based on batch-culture data in which the specific growth rate is high. This suggested that our model is not still applicable to a low dilution rate. Note that there were no experimental data for the genetic mutants cultured at a high dilution rate.

In this study, we used the experimental data of Shimizu’s group for model construction and validation. Metabolomics [27] and fluxomics were now available for many *E. coli* mutant strains under aerobic [26] and anaerobic conditions [28–30]. However, detailed experimental conditions, such as pre-cultures, aeration, pH, ingredient concentrations, reactor configuration, stirring and measurement uncertainty, were not exactly controlled among such many experiments. It is important that experimental conditions are set to the same except those that kinetic models regard as the parameters. A current dynamic model is hard to assign model parameters to all experimental conditions due to their complexity. For our model validation, the data obtained under anaerobic conditions [28] should not be used, because the model does not have any parameter to consider the anaerobic status and it was constructed under an aerobic condition. To avoid fluctuating experimental conditions or to obtain exact consistency of the simulation with experiments, a series of experiments may be performed under the strictly defined conditions by the same laboratories. Our kinetic model was built based on a series of Shimizu’s group experiments, where the condition factors that our model does not employ as the parameters are conserved over their experiments.

### Dynamic sensitivity analysis

To find critical parameters for cell growth of WT, the sensitivities of the cell concentration with respect to 38 kinetic parameters of enzyme activities (*k*_*cat*_ and *V*_*max*_) were simulated by a finite difference method [31]. The critical kinetic parameters for cell growth were sorted according to their absolute values, as shown in Table 2, where the sensitivity was sampled at 6 h (growth phase). The enzymes that catalyze the glucose uptake ($v_{Pts4}^{\hbox{max} }$,$v_{Nonpts}^{\hbox{max} }$), branching reactions (*k*_*Mdh1_cat*_*, k*_Ppc_*cat*_, $v_{G6pdh}^{\hbox{max} }$) and irreversible reactions (*k*_Pfk*_cat*_, *k*_*Fbp_cat*_) were identified as the critical factors responsible for cell growth. These results are reasonable, because glucose uptake (Pts4, nonPts) determines cell growth, the branching reactions (Ppc, G6pdh, Mdh) directly involve the metabolic flows of glycolysis and TCA cycle and irreversible enzymes are more responsible for reaction changes than reversible ones.

**Table 2:** Ranking of the critical parameters

Rank	Parameter	Sensitivity
1	$v_{Pts4}^{\hbox{max} }$	3.0405
2	$v_{Nonpts}^{\hbox{max} }$	0.8187
3	$v_{G6pdh}^{\hbox{max} }$	−0.4044
4	*k*_*Pfk_cat*_	0.3947
5	*k*_*Mdh1_cat*_	0.2211
6	*k*_*Fbp_cat*_	−0.1907
7	*k*_*Ppc_cat*_	0.1620
8	*k*_*Sdh1_cat*_	0.1179
9	*k*_*Pdh_cat*_	0.1154
10	*k*_*Cs_cat*_	0.1032
11	*k*_*Pyk_cat*_	−0.0576
12	$v_{Pta}^{\hbox{max} }$	0.0473
13	$v_{6pgdh}^{\hbox{max} }$	0.0408
14	*k*_*Mez_cat*_	−0.0393
15	$v_{Ack}^{\hbox{max} }$	0.0360
16	*k*_*Fum1_cat*_	0.0298
17	$v_{Edd}^{\hbox{max} }$	−0.0266
18	*k*_*Gapdh_cat*_	0.0248
19	*k*_*Mdh2_cat*_	0.0135
20	$v_{Ru5p}^{\hbox{max} }$	0.0090
21	*k*_*Pck_cat*_	−0.0079
22	*k*_*Pps_cat*_	0.0071
23	*k*_*Acs_cat*_	−0.0066
24	*k*_*Fba_cat*_	0.0061
25	$v_{TktA}^{\hbox{max} }$	0.0058
26	*k*_*Fum2_cat*_	0.0056
27	$v_{TktB}^{\hbox{max} }$	0.0039
28	$v_{R5PI}^{\hbox{max} }$	−0.0021
29	$v_{Eda}^{\hbox{max} }$	−0.0021
30	$v_{Pgi}^{\hbox{max} }$	0.0016
31	*k*_*Sdh2_cat*_	0.0007
32	$v_{Tal}^{\hbox{max} }$	0.0007
33	*k*_*Icdh_cat*_	0.0004
34	$k_{Glk\_cat}$	0.0002
35	*k*_*αkgdh_cat*_	0.0002
36	$v_{Pgl}^{\hbox{max} }$	−9.54E−07
37	*k*_*Ms_cat*_	6.97E−07
38	*k*_*Icl_cat*_	6.39E−07

The sensitivities were sampled at 6 h. The kinetic parameters were sorted according to their absolute values

### Prediction of multilayer regulations

Metabolic systems are typically controlled by multilayer regulations comprising gene expression, enzyme modification (e.g., phosphorylation, methylation and adenylylation) and allosteric reactions. It is difficult to directly measure those magnitudes in vivo. Only a few studies have estimated how allosteric regulations contribute to *E. coli* central carbon metabolism under different conditions [32, 33], suggesting that Pfk, Pyk and Ppc play a significant role in metabolic regulations. In this study, the kinetic model was used to predict how the regulation layers affect the dynamics of metabolic systems. We estimated the ratio of total metabolite synthesis of virtual mutants lacking specific layer regulation for the growth phase to WT (synthesis ratio).

### Gene regulation

**Fig. 7:**
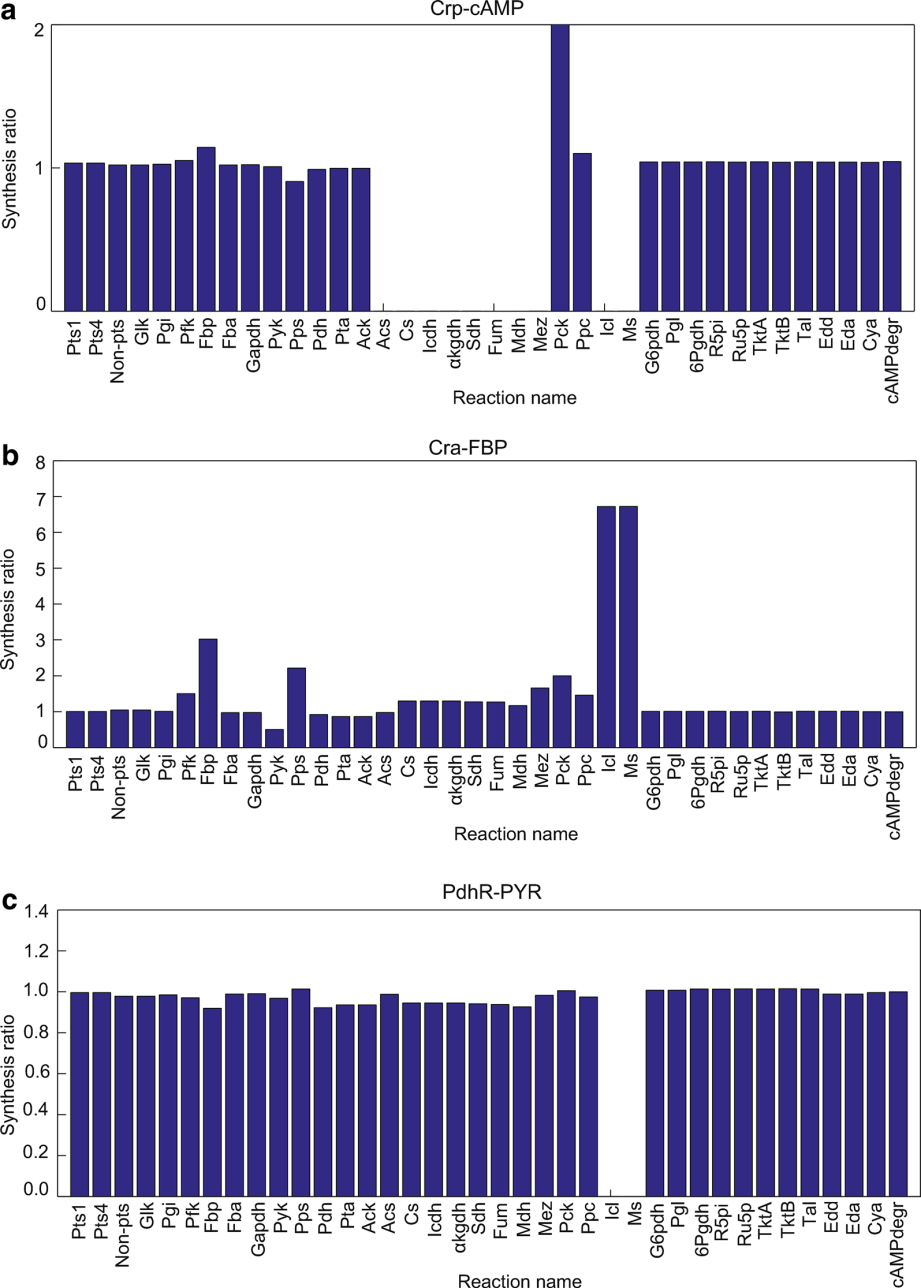
Synthesis ratios of a virtual mutant lacking a TF-metabolite complex to WT. The *x-axis* represents the reaction name and the *y-axis* represents the synthesis ratio. **a** Crp-cAMP (Pck more then 2). **b** Cra-FBP. **c** PdhR-PYR

The synthesis ratios of virtual mutants lacking binding between a TF and metabolite are shown in Fig. 7. A synthesis ratio of one indicates no difference relative to WT. A synthesis ratio of > 1 indicates that the virtual mutant enhances the total metabolite synthesis by its associated enzyme during the growth phase. In the virtual mutant lacking the Crp-cAMP complex (Fig. 7a), the synthesis ratios of the TCA cycle (Cs, Icdh, αkgdh, Sdh, Fum and Mdh), glyoxylate shunt (Icl and Ms) and acetate degradation (Acs) were remarkably suppressed; the Pck flux increased due to the increased OAA concentration (Additional file 1: Figure S4). Since the TCA cycle, which contributes to ATP production flux, was suppressed, the biomass concentration decreased relative to WT (Additional file 1: Figure S4). In the virtual mutant lacking a Cra-FBP complex (Fig. 7b), the synthesis ratios of gluconeogenesis (Fbp, Pps, Pck and Mez), glyoxylate shunt (Icl and Ms) and TCA cycle (Cs, Icdh, αkgdh, Sdh, Fum and Mdh) increased, while the synthesis ratio of glycolysis (Pyk) decreased. In Additional file 1: Figure S5, the free Cra increased the fluxes of gluconeogenesis (Fbp, Pps and Pck) and decreased the glycolysis (Pyk) flux. In the virtual mutant lacking a PdhR-PYR complex, the synthesis ratios of the Pdh, acetate production (Pta and Ack) and the TCA cycle fluxes were slightly suppressed; those of Icl and Ms were suppressed (Fig. 7c). In Additional file 1: Figure S6, Pdh flux decreased, which increased PYR and decreased the TCA cycle metabolites. Note that the synthesis ratios of Icl and Ms are very sensitive to a small perturbation to their fluxes, because their WT fluxes are very small for the growth phase.

Although the virtual mutants would be difficult to implement in vivo, we can illustrate one experimental validation. The simulated results of the virtual mutant without a Crp-cAMP complex were consistent with the experimental data for ∆*crp* [34]. The activities of the TCA cycle and glyoxylate shunt were suppressed, increasing the acetate production.

### Allosteric regulation

**Fig. 8:**
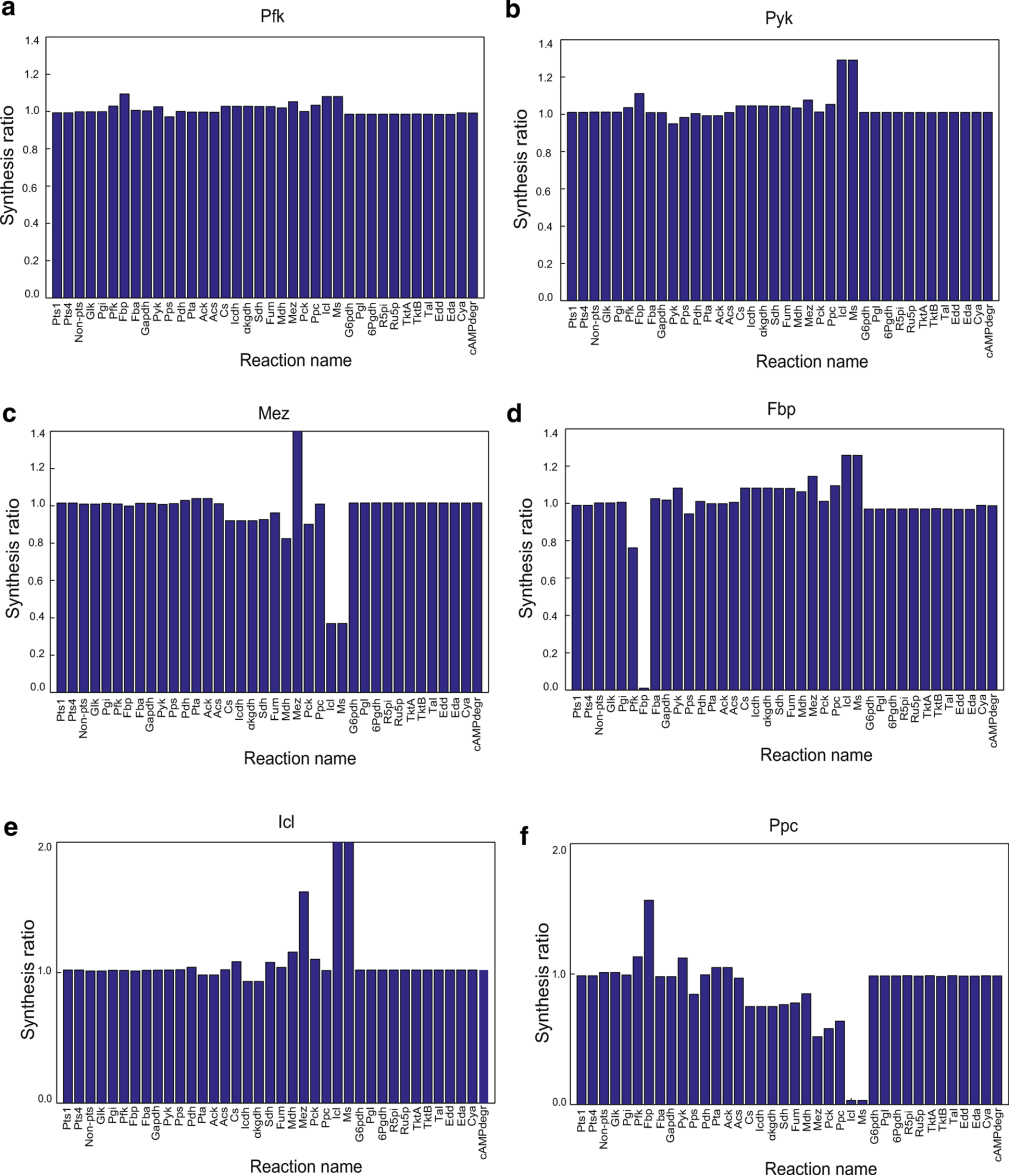
Synthesis ratios of a virtual mutant lacking allosteric reactions to WT. The *x-axis* represents the reaction name and the *y-axis* represents the synthesis ratio.** a** Pfk. **b** Pyk. **c** Mez (Mez more then 1.4). **d** Fbp. **e** Icl (Icl and MS more then 2). **f** Ppc

Allosteric enzyme activity is modulated by binding of an effector metabolite to a site other than the enzyme’s active site. We constructed a virtual mutant that prevents binding of effector metabolites to each allosteric enzyme. For the allosteric enzymes of Pgi and Cs, their synthesis ratios were close to one (data not shown), suggesting that their allosteric regulations hardly function or are little effective under the employed condition. This observation does not rule out potential functions under other stress conditions. Figure 8 shows the synthesis ratio to WT of a virtual mutant that lacks allosteric reactions with respect to six allosteric enzymes. As pointed out in [32, 33], allosteric regulations of Pfk, Pyk and Ppc showed a significant contribution to changes in metabolic flux distributions. Additional file 1: Figures S7–S12 illustrate the detailed dynamic behaviors of their virtual mutants lacking allosteric regulation.

Pfk activity is inhibited by PEP. Removal of this allosteric effect slightly increased the synthesis ratio of Pfk itself and its neighboring (Fbp and Pyk) reactions, TCA cycle (Cs, Icdh, αkgdh, Sdh, Fum and Mdh) and glyoxylate shunt (Icl and Ms) (Fig. 8a). Pyk activity is enhanced by FBP. Removal of this allosteric effect suppressed the synthesis ratio of Pyk itself and its neighboring (Pps) reaction; it increased the fluxes of the TCA cycle (Cs, Icdh, αkgdh, Sdh, Fum and Mdh) and glyoxylate shunt (Icl and Ms) (Fig. 8b). Mez activity is inhibited by AcCoA and cAMP. Loss of this allosteric inhibition increased the synthesis ratio of Mez; it decreased the fluxes of its neighboring (Mdh, Pck, Icl and Ms) reactions and the TCA cycle (Fig. 8c). Fbp activity is enhanced by PEP. Removal of this effect suppressed the synthesis ratio of Fbp itself and Pfk, while it enhanced the fluxes of the TCA cycle, anaplerotic reaction (Ppc) and glyoxylate shunt (Icl and MS) (Fig. 8d). Additional file 1: Figure S10 confirms that the Fbp and Pps fluxes decreased in the virtual mutant, increasing the net glycolysis flux. Consequently, the fluxes of both glycolysis and the TCA cycle were enhanced to increase the total ATP production flux. Interestingly, the virtual mutant of Fbp grew faster than WT. Icl activity is inhibited by PEP, GAP and αKG. Removal of their allosteric inhibition increased the synthesis ratios of Icl, Ms, Mez and Mdh and decreased the Icdh and αkgdh fluxes (Fig. 8e). Ppc activity is enhanced by FBP. Lack of this allosteric activation decreased the synthesis ratio of the Ppc, Pck and Mez, TCA cycle and glyoxylate shunt, while it enhanced the gluconeogenesis (Fbp) and acetate synthesis (Pta and Ack) fluxes (Fig. 8f).

## Discussion

### Discrepancy between simulation and experimental data

Despite extensive optimization of parameters, a small fraction of our simulated results is not well consistent with experimental data. Such discrepancies suggest biological complexity rather than mathematical difficulties in optimization. The inconsistency of the cell growth for ∆*pgi* and that of the glucose uptake for ∆*ppc* indicate that the models of ∆*pgi* and ∆*ppc* do not efficiently convert the consumed glucose into cell growth or ATP production. The model underestimated the TCA cycle fluxes for ∆*pgi*, or at a low dilution rate. They suggest that the model misses some mechanisms controlling the glucose uptake and TCA cycle under the suppressed growth.

We focus on the TCA cycle and the glucose uptake. For ∆*ppc*, PEP was accumulated to increase the glucose uptake rate (the Pts4 reaction) and the TCA cycle fluxes were suppressed because ∆*ppc* does not supply OAA to the TCA cycle. OAA is a key metabolite that drives the TCA cycle. The growth of ∆*ppc* was determined by the balance between the increased glucose uptake by accumulated PEP and the reduced TCA cycle fluxes by lack of the anaplerotic reaction. The suppressed growth of ∆*pgi* was caused by the mechanism that the reduced PEP and AcCoA decrease the fluxes of the glucose uptake and TCA cycle, respectively. Since the TCA cycle is a main source of cofactors such as NADH and FADH_2_ necessary for oxidative phosphorylation-mediated ATP production, the kinetic model can adjust the flux of the TCA cycle to alter the cell growth rate or ATP production. Actually, ATP is used not only for cell growth but also for adaptation/stress responses that regulate metabolism against unusual genetic and environmental conditions, such as gene knockout and low dilution rate. Thus, TCA cycle regulations for ATP production are critically responsible for growth estimation.

To improve our model, we suggest the following problems: (i) the dynamics of coenzymes, such as ATP, NADH and NADPH, are not considered, which alter energy and redox status; (ii) the dissolved oxygen concentration is not incorporated, which directly impacts ATP production; (iii) information on the interaction between central carbon metabolism and peripheral subnetworks such as amino acid, protein, lipid and nucleotide syntheses is lacking; (iv) some regulatory networks remain to be revealed. For (i), the flux of the TCA cycle, which directly relates to ATP production, was underestimated for ∆*pgi* and for WT at a low dilution rate. TCA cycle flux could be affected by the fluxes of the anaplerotic pathway at PEP, acetate production pathway at AcCoA and glyoxylate shunt at AcCoA and ICIT. In addition, the rates of such metabolic reactions are directly modulated by coenzyme concentrations. Thus, lack of coenzyme dynamics would cause inconsistencies with experimental data. Cofactor concentrations (e.g., ATP, NADH, NADPH) vary with time, depending on growth phase and genetic conditions. To integrate the variations in the cofactor concentrations, we need to include all of the reactions with cofactors, but it is difficult at the moment because many of the reactions with cofactors are outside the central carbon metabolism. For (ii), the kinetic model does not consider a decrease in dissolved oxygen, which is consumed by oxidative phosphorylation to produce ATP from NADH and FADH_2_; however, in a real batch culture, dissolved oxygen would decrease with increasing cell growth. In the late growth phase, metabolic reactions involving such coenzymes would be affected by a shortage of dissolved oxygen. Considering the dynamics of dissolved oxygen would be effective in improving the prediction accuracy of the kinetic model.

### Advantages of our kinetic model

The proposed kinetic model is the first model to our knowledge that reproduces the dynamics of multiple genetically modified mutants under an aerobic condition in a batch culture. Our model has strong advantages over previous models: (i) It employs literature-based detailed kinetic equations with gene regulations to accurately reproduce experimental data for WT and multiple genetic mutants in a batch culture, while estimating a specific cell growth rate based on the ATP production mechanism. (ii) The values of many kinetic parameters were extensively estimated by a constrained optimization technique on a supercomputer. (iii) Our model predicts the effects of multilayer regulations (allosteric effectors and gene expressions) on central carbon metabolism and enables rational design of fast-growing cells.

### Comparison with existing dynamic models

To characterize the performance of our kinetic model, we simulated other existing models for WT and genetic mutants. The Matsuoka [19], Kotte [18], Usuda [21] and Kadir [18] models were employed as reference models for a batch culture. In addition, the Chassagnole model [13] was used as a classical reference model for a continuous culture. The Matsuoka model reproduced the experimental time courses of extracellular glucose and biomass of WT and ∆*pykF*, whereas it neither reproduced the dynamics of ∆*pgi* nor ∆*ppc* (Additional file 2: Figure S13). The cell growth of ∆*ppc* was accelerated compared with WT, which is opposite to the experimental data. The Kotte model uses the Monod equation to simulate the glucose uptake, without estimating the specific growth rate based on molecular processes. The Kotte model reproduced the experimental time courses for WT and ∆*pykF*, but it did neither for ∆*pgi* nor ∆*ppc* (Additional file 2: Figure S14). The Usuda model incorporates glutamine/aspartate metabolism, fructose consumption and a TF (Mlc) involving the Pts. The Usuda model required the experimenatal growth data to simulate the WT model. Since it does not estimate the specific cell growth rate, it is not available for a batch culure of genetic mutants whose cell growth is varied. The Kadir model is a kinetic model to reproduce the dynamics of genetic mutants in both batch and continuous cultures, but their model has a serious problem: the values of some parameters responsible for cell growth and gene regulations are changed with respect to each genetic mutant. True dynamic modeling should use fixed kinetic parameter values across conditions. The Chassagnole model has been a classical reference model that consists of glycolysis and pentose phosphate pathways and simulated the steady state level of WT and a transient response to glucose pulses. The model is neither applicable to a batch culture nor genetic mutants, because it does not estimate the specific cell growth rate. In this study we simulated the Chassagnole model at a dilution rate of 0.5 and 0.7 h^−1^ in a continuous culture (Additional file 2: Figure S15). The consistency of our kinetic model with experimental data (Fig. 6) was almost the same as that of the Chassagnole model.

To challenge a key problem in cell growth estimation, use of the total ATP production flux is a reasonable choice because it is directly related to cell growth. The total ATP production flux is determined by quantifying the contribution of each utilized pathway: glycolysis, TCA cycle and oxidative phosphorylation under aerobic conditions. The total ATP production flux reflects the reconstituted metabolic pathways caused by genetic changes. The Matsuoka and Kadir models used the total ATP production flux for cell growth estimation and multiplied it by the Monod equation with extracellular glucose to fit experimental data. However, since the glucose uptake flux was defined as Pts protein-mediated influx, multiplication by the Monod equation seems redundant or unnecessary. According to the original idea, the specific growth rate should be determined by the total ATP production flux, not by the Monod equation. Our kinetic model is the first to estimate a specific growth rate function that is linear to the total ATP production flux to overcome existing problems.

### Powerful optimization required

Current kinetic models have used literature-based kinetic equations with measured parameters in vitro. Those kinetic equations can be combined to construct a large-scale model, but the parameter values measured in vitro are not always appropriate to simulate in vivo behaviors. Particularly, maximum velocities, V_max_, are unknown in vivo because the exact absolute concentrations and activities of their associated enzymes have not extensively been measured in vivo. Thus, parameter estimation is required to construct in vivo kinetic models. Since the number of undetermined parameters increases with increased network size, the values of many parameters must be estimated so that the model can reproduce the experimental data. Massive calculation power with constrained evolutionary search methods [35, 36] was required to estimate the unknown values of many parameters. We iterated 4 × 10^7^ simulations to estimate 351 parameters using a genetic algorithm over 8 days with 101 cores of Intel Xeon E5-2670 v3 2.3 GHz on a super computer.

### Rational understanding and design

How multilayer regulations determine metabolic fluxes has been intensively discussed and remains controversial, because it is difficult to measure the contribution of a specific regulatory module to the entire system in vivo by constructing a genetically modified mutant that lacks the regulation. Instead, a kinetic model with a virtual mutant whose specific reaction is disabled is used to estimate the contribution of the specific regulation to a central carbon metabolism. Expectedly Crp- and Cra- mediated regulations of metabolic genes shifted metabolic states among glycolysis, gluconeogenesis and TCA cycle. On the other hand, allosteric regulations of Pfk, Pyk, Mez, Fbp, Icl and Ppc were found to play a critical role in metabolic shift. Interestingly, a virtual mutant lacking allosteric regulation of Fbp was predicted to grow faster than WT; the other mutants did not. This shows a great example of a rational design of fast-growing cells based on understandings of molecular processes.

We discussed the feasibility of engineering such fast-growing cells. Adaptive laboratory evolution (ALE) has been studied to explore the optimal growth of *E. coli* [37–39]. It showed that the cell growth rate could be improved by evolutional mutation, although the mutation sites obtained by such evolutional experiments were not consistent with our virtual mutants. In our previous work [40], a rationally designed *mlc* knockout mutant with *ptsI* gene overexpression increased the specific glucose uptake rate and a *ptsI* gene overexpressing mutant increased both the specific glucose uptake rate and cell growth. Those successful examples suggest the possibility of rationally designing fast-growing strains.

### Towards whole-cell simulation of *E. coli*

The hypotheses generated from the inconsistencies between the simulation and experimental results need to be tested carefully with further experiments and iterative modeling, providing information on bottlenecks of cellular systems. Extension of the model with greater accuracy and integration of simulation with measurements is a powerful methodology for rational improvement and design of metabolic and gene regulatory networks.

In the near future, an extended kinetic model of an *E. coli* central carbon metabolism will be built that includes both sugar (carbon source) uptake [40] and ammonium ion (nitrogen source) uptake because nitrogen sources are responsible for synthesizing amino acids and nucleotides. The ammonia assimilation system consists of complex metabolic reactions, protein signal transduction and gene expression regulation to uptake a low concentration of environmental ammonium ions and directly connects to the TCA cycle through αKG and coenzymes such as ATP and NADPH. To date, several models have been developed that reproduced some dynamics under ammonia starvation [41–43] and revealed mechanisms of how the ammonia assimilation system is robust to ammonia starvation [8, 44]. However, there has been few detailed kinetic model of central carbon metabolism with sugar uptake systems and the ammonia assimilation system.

The proposed kinetic model remains to be improved to adapt many genetic and environmental changes. A comprehensive dynamic model, called a virtual *E. coli*, should be constructed to reproduce complex dynamics of a series of genetic mutants under different conditions, such as sugar starvation, nitrogen starvation, anaerobic conditions, osmotic pressure and a change in pH and combined with systematic experiments. The virtual *E. coli* is a “blueprint” unicellular organism that is advantageous because the overall central metabolic pathway structure is remarkably conserved. It provides a platform for understanding the design principles underlying molecular processes, rational design of useful material production and further modeling efforts of a variety of cells.

## Conclusions

*Escherichia coli* metabolic pathways have been modeled extensively at the enzymatic and genetic levels, but existing models cannot accurately reproduce experimental behaviors in a batch culture, due to inadequate estimation of cell growth function based on its molecular processes and a large number of unmeasured parameters. We developed a detailed kinetic model for the central carbon metabolism of *E. coli* in a batch culture, which includes the glycolytic pathway, tricarboxylic acid cycle, pentose phosphate pathway, Entner-Doudoroff pathway, anaplerotic pathway, glyoxylate shunt, oxidative phosphorylation, Pts, non-Pts and metabolic gene regulations by four protein transcription factors. The kinetic parameter values were extensively estimated by a constrained optimization method on a supercomputer. The model estimated a specific growth rate based on reaction kinetics and accurately reproduced the dynamics of WT and multiple genetic mutants in a batch culture. This model overcame the intrinsic limitations of existing kinetic models, predicted the effects of multilayer regulations on central carbon metabolism and proposed rationally designed fast-growing cells based on understandings of molecular processes.

## Methods

### Kinetic model

We used the following kinetic model for a batch or continuous culture:

1 $$\frac{dX}{dt} = {\rm{\mu \bf{(x}}}_{ 1} , {\rm\bf{{x}}}_{ 2} , {\rm\bf{{y},\bf{p})}}X - DX$$

2 $$\frac{{d\rm\bf{{y}}}}{dt} = D ( {\rm\bf{{y}}}_{feed} - {\rm\bf{{y}}}) - \rm\bf{{g}}({\rm\bf{{x}}}_{1} ,{\rm\bf{{x}}}_{2} ,{\rm\bf{{y}}},{\rm\bf{{p}}})\it{X}$$

3 $$\frac{{d\rm\bf{{x}}_{1} }}{dt} = {\rm\bf{{f}(x}}_{ 1} , {\rm\bf{{x}}}_{ 2} , {\rm\bf{{y,p})}}$$

4 $$\rm\bf{{x}}_{2} = {\rm\bf{{h(x}}}_{ 1} , {\rm\bf{{y,p})}}$$

The equations show the dynamics of a batch culture when the dilution rate, *D*, is set to zero. *X* is the cell concentration in a reactor,** x**_1_ is the vector of 50 time-varying molecule (metabolite, enzyme and Pts protein) concentrations,** x**_2_ is the vector of ancillary variables including intracellular protein (TF and TF-metabolite complex) concentrations,** y** is the vector of extracellular substrate (glucose and acetate) concentrations,** y**_*feed*_ is the substrate concentration vector of the feed,** p** is the vector of 341 kinetic or constant parameters, μ(**x**_1_,** x**_2_,** y**,** p**) is the specific growth rate,** g**(**x**_1_,** x**_2_,** y**,** p**) is the function vector of specific substrate (glucose and acetate) uptake rates,** f**(**x**_1_,** x**_2_,** y**,** p**) is the function vector of mass balance equations with respect to** x**_1_. The IclR concentration was set to a constant value. Details of the mass balance equations, reaction rates and ancillary variables are presented in Additional file 3: Tables S1–S3. All molecule concentrations are represented in units of millimolar (mM). Details of the initial concentrations and parameters are presented in Additional file 3: Tables S4–S6.

The visualization of the simulated results was performed using MATLAB R2014b on a personal computer (Windows 7 Professional, Intel Core i7-3770 3.40 GHz, RAM 8.00 GB).

### Cell growth rate estimation

A specific cell growth rate was estimated based on its direct connecting to ATP production flux. The total ATP production flux is given by:

5 $$\begin{aligned} v_{ATP} = OP_{NADH} + OP_{{FADH_{2} }} - v_{E,Glk} - v_{E,Pfk} + v_{E,Gapdh} + v_{E,Pyk} - v_{E,Pps} \\ + v_{E,Ack} - v_{E,Acs} + v_{E,\alpha kgdh} - v_{E,Pck} - v_{E,AceK - ki} - v_{E,Cya} \\ \end{aligned}$$

$OP_{NADH}$ and $OP_{{FADH_{2} }}$ are the specific ATP production fluxes through oxidative phosphorylation as follows:

6a $$OP_{NADH} = \left( {v_{E,Gapdh} + v_{E,Pdh} + v_{E,\alpha kgdh} + v_{E,Mdh} } \right) \times (P/O),$$

6b $$OP_{{FADH_{2} }} = v_{E,Sdh} \times (P/O) ^ {\rm{\prime}},$$

where (*P/O*) and (*P/O*)′ indicate the Phosphate/Oxygen (P/O) ratios for NADH and FADH_2_, respectively under aerobic conditions. Assuming that a specific cell growth rate, µ, is linearly correlated with the specific ATP production rate [18, 19, 25], it is given by:

7 $$\mu = k_{ATP} v_{ATP} ,$$

where *v*_*ATP*_ is the specific ATP production flux computed by Eq. (5) and *k*_*ATP*_ is the adjustable constant.

The biomass flux is estimated from twelve precursor metabolites (G6P, F6P, GAP, PEP, PYR, AcCoA, αKG, SUC, FUM, OAA, R5P and E4P) as follows:

8 $$v_{BM,\cdot} = (\alpha_{GLC} k_{BM\_GLC\_\cdot } + \alpha_{ACE} k_{BM\_ACE\_\cdot } )[ \cdot ]$$

The detailed rate equations for twelve precursor metabolites are shown in Additional file 3: Table S2. Here, $[ \cdot ]$ indicates the concentration of the corresponding metabolite and $k_{BM\_GLC\_\cdot } ,k_{BM\_ACE\_\cdot }$ are the first-order rate constants for that metabolite. $\alpha_{GLC}$ and $\alpha_{ACE}$ are weights that indicate which carbon source is available at a moment, given by:

9a $$\alpha_{GLC} = \frac{{[GLC^{ex} ]}}{{[GLC^{ex} ] + K_{Pts\_GLC} }},$$

9b $$\alpha_{ACE} = \frac{{[ACE^{ex} ]}}{{[ACE^{ex} ] + K_{Acs\_ACE} }}\left( {1 - \alpha_{GLC} } \right),$$

where $K_{Pts\_GLC}$ and $K_{Acs\_ACE}$ are the affinity constants.

### Parameter estimation

We estimated 351 model parameter values using limited amount of experimental data. Thus, most of parameter values are not identifiable. It is often costly and time-consuming to collect enough experimental data to identify all of those model parameters. This is a common situation in kinetic modeling. Instead, we developed a constrained optimization method to estimate parameter values by using limited experimental data and available measured parameter values. We assumed that in vitro measured values are close to in vivo values. The constrained optimization seeks reasonable parameter values, i.e., they are close to the in vitro measured values and allows the model to reproduce in vivo behaviors. This approach does not guarantee the estimated parameter values are ‘true’. However, at least, use of the estimated parameter values is the most realistic choice, considering the currently available experimental data. Details in parameter estimation are shown in Additional file 4.

The parameter estimation problem is formulated as a constrained optimization problem:

10a $$\rm{Minimize}\,obj\_fun(\rm\bf{{p}}),$$

10b $$\rm{Subject\,to}\,\rm\bf{{constraint}}\_\rm\bf{{fun}}(\rm\bf{{p}}) \, \bf{\le 0},$$

where $obj\_fun$ is the objective function that evaluates the difference between the estimated parameters and literature-based parameters. $\rm\bf{{constraint}}\_\rm\bf{{fun}}$ consists of multiple if–then rule-based score functions that evaluate whether the simulated behavior is consistent with experimental data [35]. We categorized search parameters into three groups: Classes I, II and III. Class I comprises the parameters for which measured values are available. Class II includes the parameters whose values can be estimated from references. Class III encompasses the parameters for which quantitative information is lacking. Penalty weights λ_1_, λ_2_ and λ_3_ were used for Class I, II and III, respectively. If the values of Class I parameters change drastically, large penalties are imposed (*λ*_1_ > *λ*_2_ > *λ*_3_ ≥ 0). Therefore, such parameter values are unlikely to survive to the end of the optimization process.

To solve the constrained optimization problem, we employed genetic algorithms (GAs) with UNDX [45] as a crossover method and Minimum Gap Generation (MGG) [46] as a generation alternation method. The optimization processes were parallelized using the Message Passing Interface on the super computer Shirokane3 provided by Human Genome Center, The University of Tokyo. The GAs were performed on the 101 cores of Intel Xeon E5-2670 v3 2.3 GHz. The estimated parameters are presented in Additional file 3: Tables S4–S6.

### Sensitivity analysis

The finite difference method is used to simulate dynamic sensitivities [31]. It is an indirect differential method that approximates the change in dependent variables with respect to a minute change in time-independent parameters. The dynamic sensitivities are given by:

11 $$s(x_{i} ,p_{j} ) = \frac{{\partial x_{i} }}{{\partial p_{j} }}\frac{{p_{j} }}{{x_{i} }} \cong \frac{{\Delta x_{i} }}{{\Delta p_{j} }}\frac{{p_{j} }}{{x_{i} }} = \frac{{x_{i} (t,p_{j} + \Delta p_{j} ) - x_{i} (t,p_{j} )}}{{\Delta p_{j} }}\frac{{p_{j} }}{{x_{i} }}$$

$$\Delta p_{j} = \eta \cdot p_{j}$$

where $\eta$ is the perturbation coefficient, *i* is the time index and *j* is the index of independent parameters. In this analysis, we calculated $s(X,p_{j} )$, the sensitivity of cell growth (*X*) with respect to 38 kinetic parameters of enzyme activities (*kcat* and *Vmax*). A perturbation coefficient of 0.01 was used.

### Correlation coefficient

The Pearson’s correlation coefficient between the simulated fluxes and experimental data was calculated with p values. A high value of *r* indicates high consistency. The p values are used for testing the hypothesis that the simulated and experimental fluxes are uncorrelated. (For example, reject the hypothesis at the 5 % level of significance having p < 0.05).

### Virtual genetic mutants

To characterize the behaviors of the model, we used mathematical comparisons between WT and “virtual” mutants where specific reactions are disabled [47]. The virtual mutants alter the reaction network structure without changing other kinetic parameters, enabling deletion of specific reactions that cannot be accomplished through biological experiments. In this study, we specifically deleted binding between TFs and metabolites and allosteric binding between effector metabolites and an enzyme. To suppress TF-metabolite binding, we increased the parameter values of *K*_*Crp*-*cAMP*_, *K*_*Cra*-*FBP*_ and *K*_*PdhR*-*PYR*_ 10^5^-fold in the equations for Crp-cAMP, Cra-BP and PdhR-PYR, respectively (Additional file 3: Table S3). To suppress allosteric regulation of Pfk, Pyk, Cs, Pgi, Icl, Mez, Fbp and Ppc, we increased the values of *K*_*Pfk_PEP*_, *K*_*Pyk_FBP*_, *K*_*Cs_αKG*_, *K*_*Pgi_G6P_6pginh*_, *K*_*Icl_PEP*_, *K*_*Icl_3PG*_, *K*_*Icl_αKG*_, *K*_*Mez_AcCoA*_, *K*_*Mez_cAMP*_, *K*_*Fbp_PEP*_ and *K*_*Ppc_FBP*_ by 10^5^-fold (Additional file 3: Table S2 for the rate equation of Pfk, Pyk, Cs, Pgi, Icl, Mez, Fbp and Ppc).

### Synthesis ratio

To estimate the contribution of specific regulation to the metabolite fluxes, we defined the synthesis ratio to WT of a virtual mutant by:

12 $$Synthesis\,ratio = \frac{{\int\nolimits_{0}^{{T_{VM} }} {v_{VM,enzyme} \frac{\left[ X \right]}{\rho }dt} }}{{\int\nolimits_{0}^{{T_{WT} }} {v_{WT,enzyme} \frac{\left[ X \right]}{\rho }dt} }} ,$$

where *T*_*WT*_ and *T*_*VM*_ are the end time of the growth phase of WT and virtual mutants, respectively, $v_{WT,enzyme}$ is the metabolic flux of WT and $v_{VM,enzyme}$ is the metabolic flux of a virtual mutant.

### Experimental data

*Escherichia coli* K-12 strain BW25113 (*lacI*^q^*rrnB*_T14_ ∆*lacZ*_WJ16_*hsdR514* ∆*araBAD*_AH33_ ∆*rhaBAD*_LD78_) was cultured in the synthetic medium in a batch culture [25]. Cultivation of WT and mutant (∆*pgi*, ∆*pykF*) strains was conducted at 37 °C in a working volume of 1.2 L in a 2-L reactor equipped with pH, dissolved oxygen and temperature sensors. The air flow rate was maintained at 1 L/min and the pH was maintained at 7.0 by automatic addition of HCl or NaOH throughout cultivation. The same *E. coli* strain was cultured in the synthetic medium in a continuous culture [26] where the dilution rate was set at 0.1, 0.2, 0.4, 0.5 or 0.7 h^−1^. Cultures were grown aerobically at 37 °C in a total working volume of 1 L, in a 2-L jar fermenter equipped with pH, dissolved oxygen concentration and temperature sensors and a turbidity meter. The air flow rate was kept at 1 L/min and pH was maintained at 7.0 by automatic addition of HCl or NaOH for the duration of the culture. Table 1 summarizes the training and testing datasets employed by our model construction and validation. Details are described in Additional file 4.

### Availability of data and materials

The kinetic model: the MATLAB version and the SBML version that consists of SBML (model) and SED-ML (simulation settings) files are freely available for non-commercial use according to the GNU Free Documentation License (GFDL) at:

http://www.cadlive.jp/cadlive_main/Softwares/KineticModel/Ecolimetabolism.html.

MATLAB2007 or higher is required. Our project, named Computer-Aided Design Living System (CADLIVE) Project, is described at http://www.cadlive.jp/.

## Additional files

10.1186/s12934-016-0511-x Simulation results.
10.1186/s12934-016-0511-x Comparison of our kinetic model with other existing models.
10.1186/s12934-016-0511-x Details of the kinetic model.
10.1186/s12934-016-0511-x Details of the parameter estimation method.

## Abbreviations

αKG: α-ketoglutarate
6PG: 6-phosphogluconate
6PGL: 6-phosphogluconolactonase
AcCoA: acetyl-CoA
AcP: acetyl phosphate
ACE^ex^: external acetate
ATP: adenosine-5′-triphosphate
ADP: adenosine-5′-diphosphate
AMP: adenosine-5′-monophosphate
cAMP: cyclic AMP
CoA: coenzyme A
E4P: erythrose-4-phosphate
F6P: fructose-6-phosphate
FBP: fructose-1,6-bisphosphate
FUM: fumarate
G6P: glucose-6-phosphate
GAP: glyceraldehyde-3-phosphate
GLC^ex^: external glucose
GOX: glyoxylate
ICIT: isocitrate
KDPG: 2-keto-3-deoxy-6-phosphogluconate
MAL: malate
NAD: nicotinamide adenine dinucleotide
NADH: nicotinamide adenine dinucleotide, reduced
NADP: dihydronicotinamide adenine dinucleotide phosphate
NADPH: dihydronicotinamide adenine dinucleotide phosphate, reduced
OAA: oxaloacetate
PEP: phosphoenol pyruvate
PYR: pyruvate
Pi: inorganic phosphate
R5P: ribose-5-phosphate
RU5P: ribulose-5-phosphate
S7P: sedoheptulose-7-phosphate
SUC: succinate
X5P: xylulose-5-phosphate
αkgdh: α-keto-D-gluconate dehydrogenase
6Pgdh: 6-phosphogluconate dehydrogenase
AceK: isocitrate dehydrogenase phosphatase/kinase
Ack: acetate kinase
Acs: acetyl coenzyme A synthetase
Cra: catabolite repressor/activator
Crp: cAMP receptor protein
Cs: citrate synthase
Cya: adenylate cyclase
Eda: 2-keto-3-deoxygluconate 6-phosphate aldolase
Edd: 6-phosphate dehydrase
EIIA: unphosphorylated Pts protein EIIA
EIIA-P: phosphorylated Pts protein EIIA
Fba: fructose-1,6-bisphosphate aldolase class II
Fbp: fructose-1,6-bisphosphatase
Fum: fumarase
G6pdh: glucose-6-phosphate dehydrogenase
Gapdh: glyceraldehyde 3-phosphate dehydrogenase
Glk: glucokinase
Icdh: isocitrate dehydrogenase
Icdh-P: phosphorylated isocitrate dehydrogenase
Icl: isocitrate lyase
IclR: isocitrate lyase regulator
Ms: malate synthase
Mez: malic enzyme
Mdh: malate dehydrogenase
Pck: phosphoenolpyruvate carboxykinase
Pdh: pyruvate dehydrogenase
PdhR: pyruvate dehydrogenase complex repressor
Pfk: phosphofructokinase
Pgi: phosphoglucose isomerase/glucosephosphate isomerase
Pgl: phosphogluconolactonase
Ppc: phosphoenolpyruvate carboxylase
Pps: phosphoenolpyruvate synthase
Pta: phosphotransacetylase
Pyk: pyruvate kinase I
Rpe: ribulose phosphate 3-epimerase
Rpi: ribulose 5-phosphate 3-isomerase
Sdh: succinate dehydrogenase
Tal: transaldolase
TktA: transketolase I
TktB: transketolase II
