# Supplementary material for: Development of an accurate kinetic model for the central carbon metabolism of Escherichia coli
Source: Microb Cell Fact. 2016 Jun 21;15:112. doi: 10.1186/s12934-016-0511-x (PMC4915146; doi:10.1186/s12934-016-0511-x)
Supplement: Supplementary file 1 — 10.1186/s12934-016-0511-x Simulation results. [file 12934_2016_511_MOESM1_ESM.pdf]

Simulation results

A

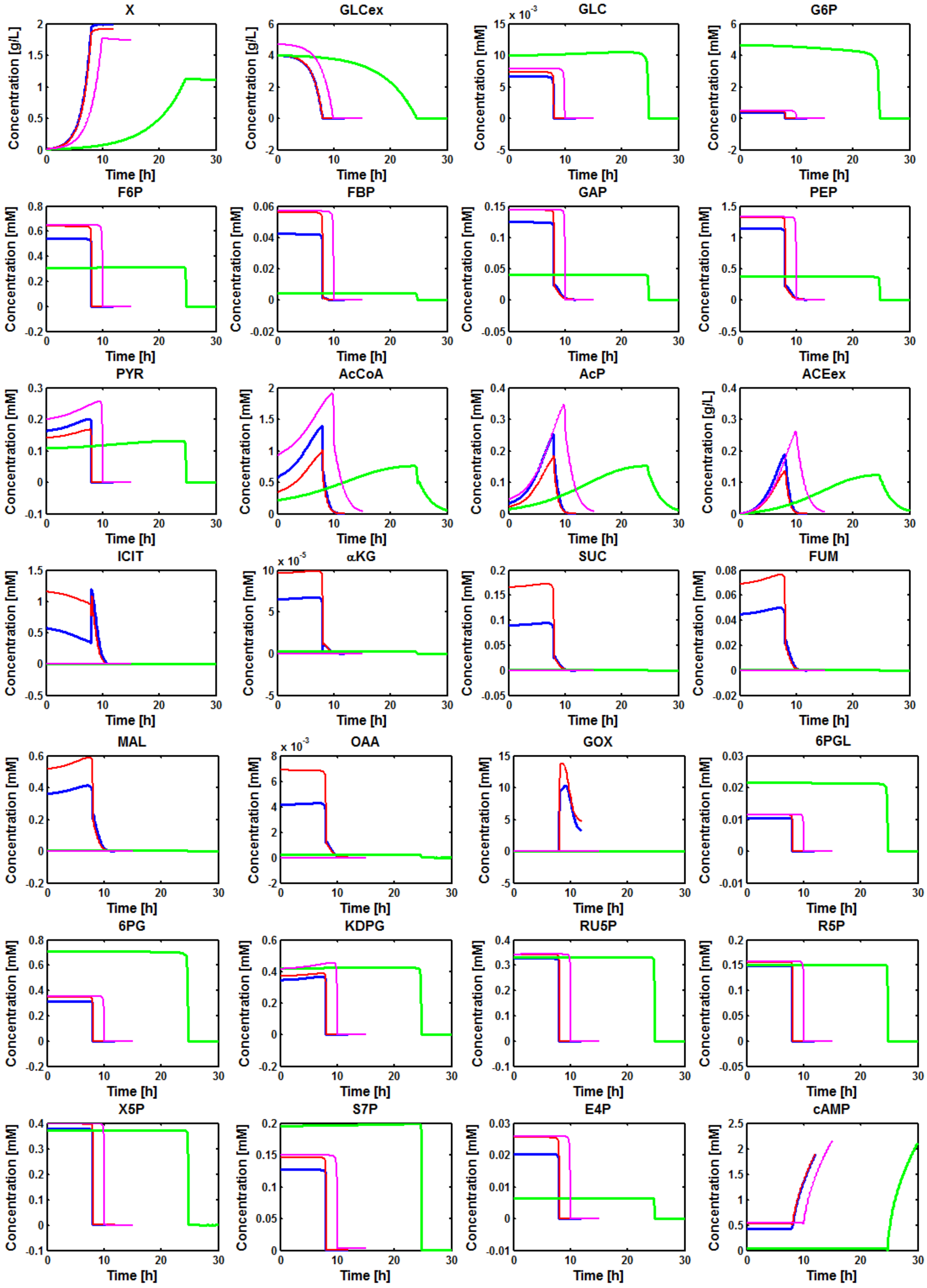

B

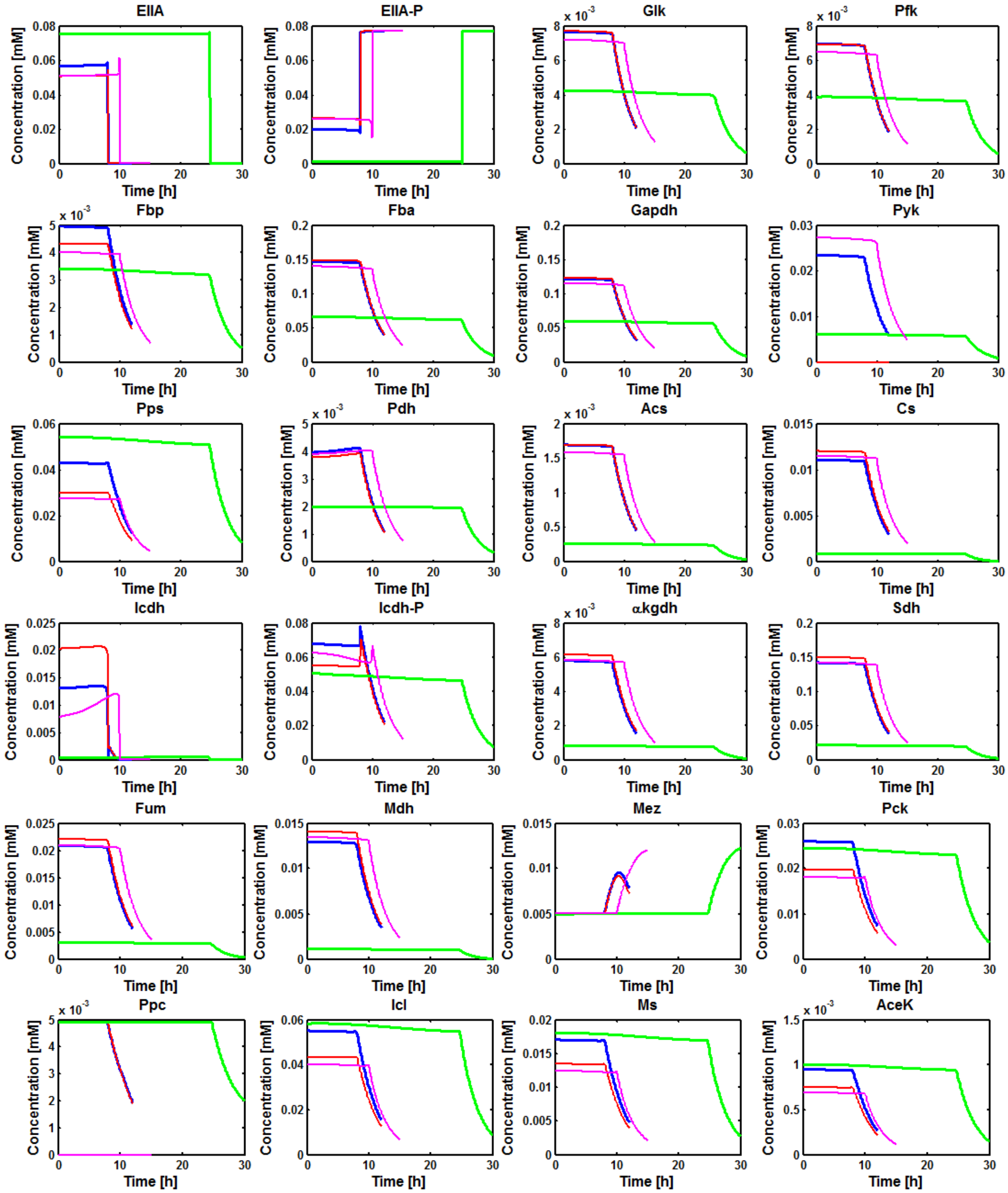
EIIA
EIIA-P
Glk
Pfk
Fbp
Fba
Gapdh
Pyk
Pps
Pdh
Acs
Cs
Icdh
Icdh-P
αkgdh
Sdh
Fum
Mdh
Mez
Pck
Ppc
Icl
Ms
AceK
Concentration [mM]
Time [h]
x 10^{-3}

C

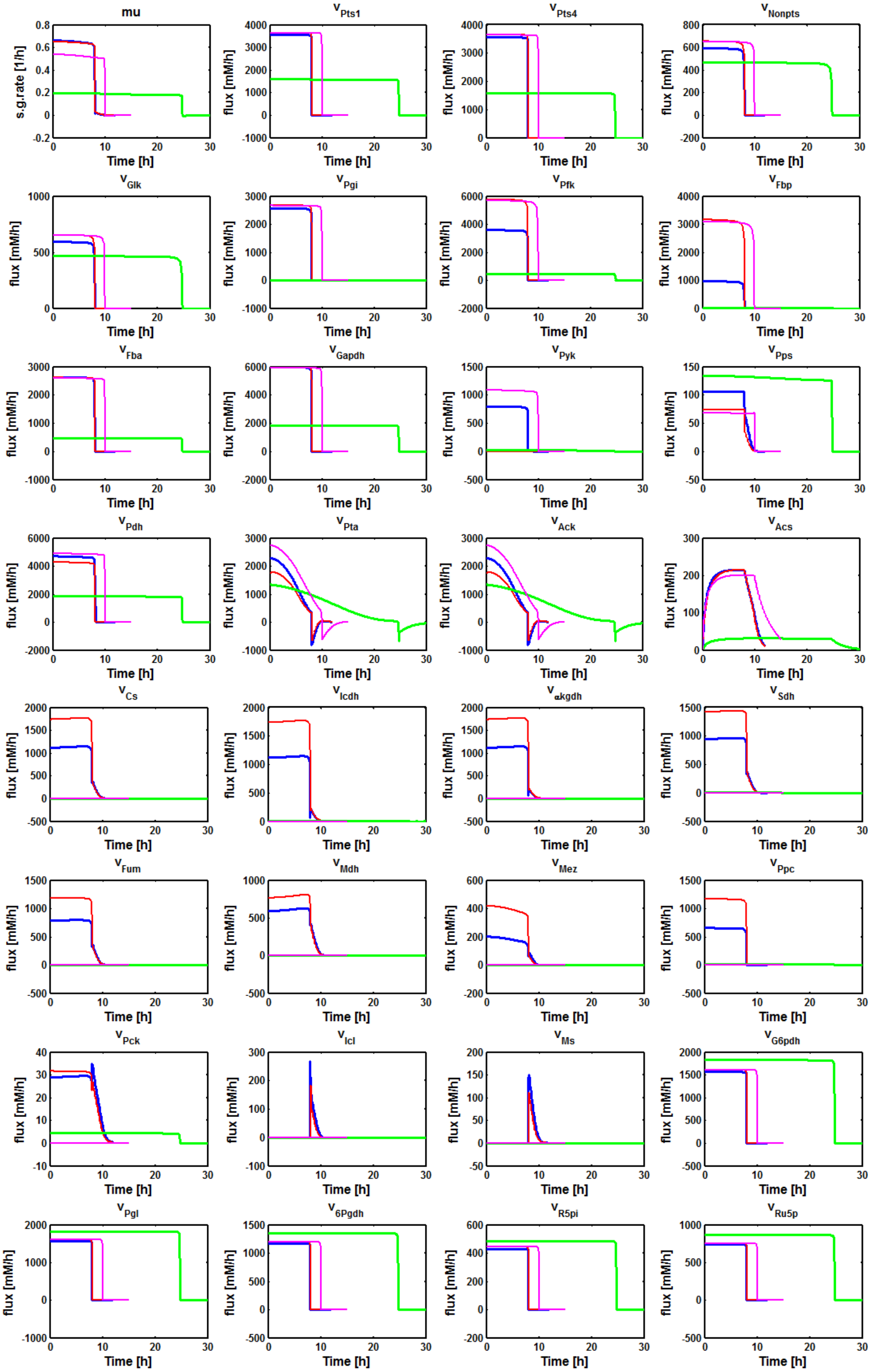
mu
s.g.rate [1/h]
v_{Pts1}
v_{Pts4}
v_{Nonpts}
v_{Glk}
v_{Pgi}
v_{Pfk}
v_{Fbp}
v_{Fba}
v_{Gapdh}
v_{Pyk}
v_{Pps}
v_{Pdh}
v_{Pta}
v_{Ack}
v_{Acs}
v_{Cs}
v_{Icdh}
$v_{\alpha kgdh}$
v_{Sdh}
v_{Fum}
v_{Mdh}
v_{Mez}
v_{Ppc}
v_{Pck}
v_{Icl}
v_{Ms}
v_{G6pdh}
v_{Pgl}
v_{6Pgdh}
v_{R5pi}
v_{Ru5p}
flux [mM/h]
Time [h]

C (continue…)

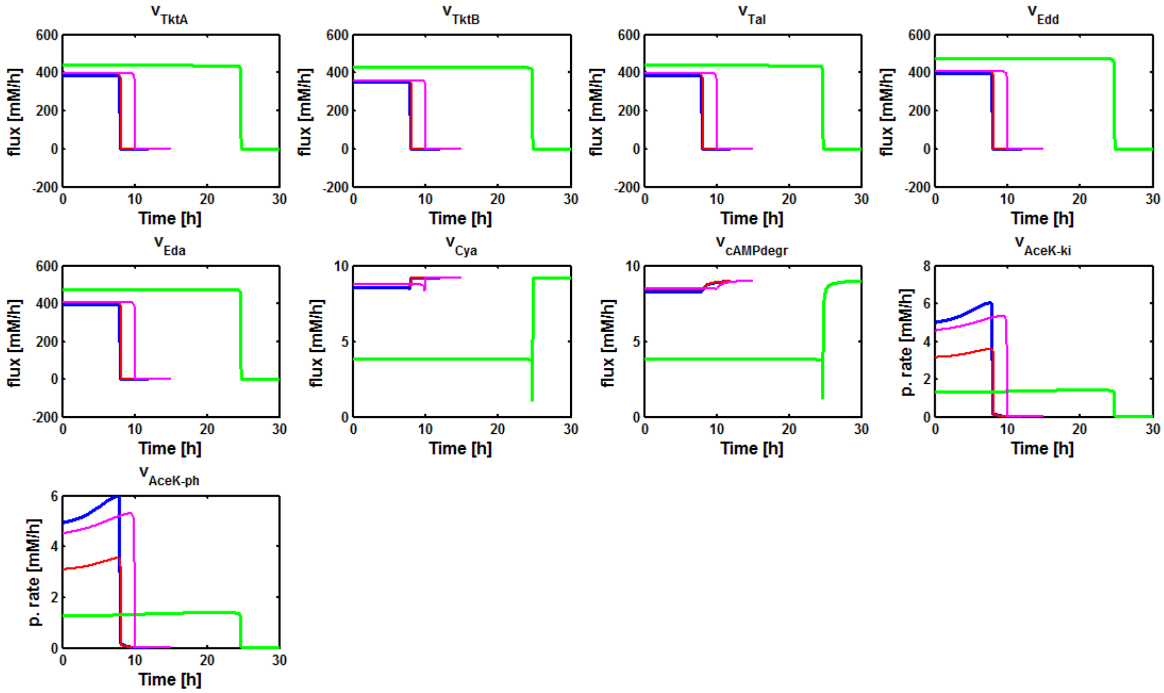

Figure S1: Simulated time course of WT, Δ*pykF* , Δ*pgi* and Δ*ppc*

The blue, red, green, and magenta lines indicate WT, Δ*pykF*, Δ*pgi* and Δ*ppc*, respectively.

A. Metabolite concentrations; **B.** Enzyme concentrations; **C.** Rates (Specific growth rate [s.g.rate]; flux; phosphorylation rate [p.rate]).

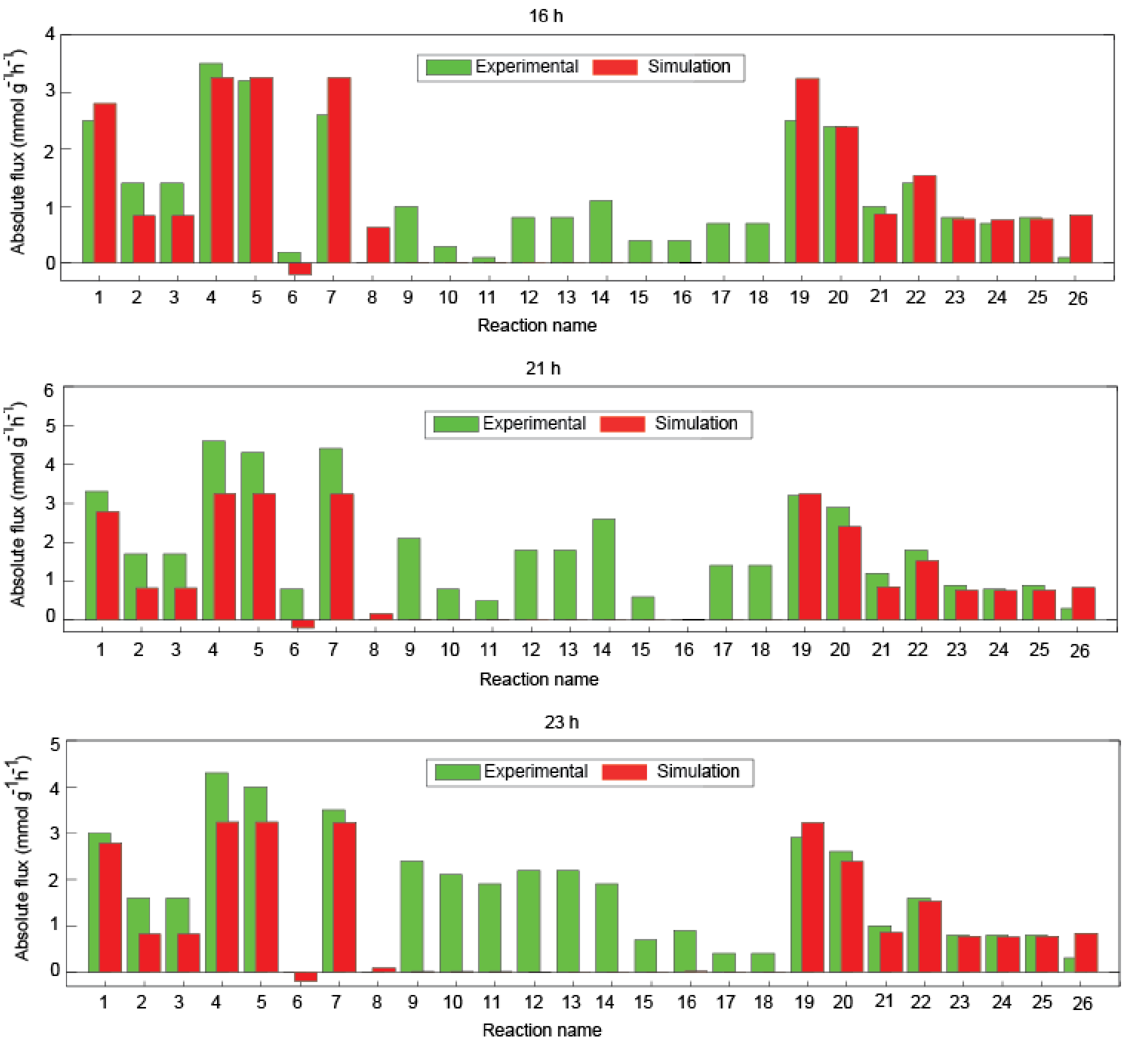

Figure S2: Comparison of the simulated flux with the experimental flux [1] for Δ*pgi* at 16 h, 21h and 23h, respectively.

The indexes of the reaction names indicate 1: v_Pts4, 2: vE_Pfk-vE_Fbp, 3: vE_Fba, 4: vE_Gapdh, 5: vE_Gapdh, 6: vE_Pyk-vE_Pps, 7: vE_Pdh, 8: vE_Pta, 9: vE_Cs, 10: vE_Icdh, 11: vE_αkgdh, 12: vE_Sdh, 13: vE_Fum, 14: vE_Mdh, 15: vE_Mez, 16: vE_Ppc-vE_Pck, 17: vE_Icl, 18: vE_Ms, 19: vE_G6pdh, 20: vE_6Pgdh, 21: vE_R5pi, 22: vE_Ru5p, 23: vE_TktA, 24: vE_TktB, 25: vE_Tal, 26: vE_Edd.

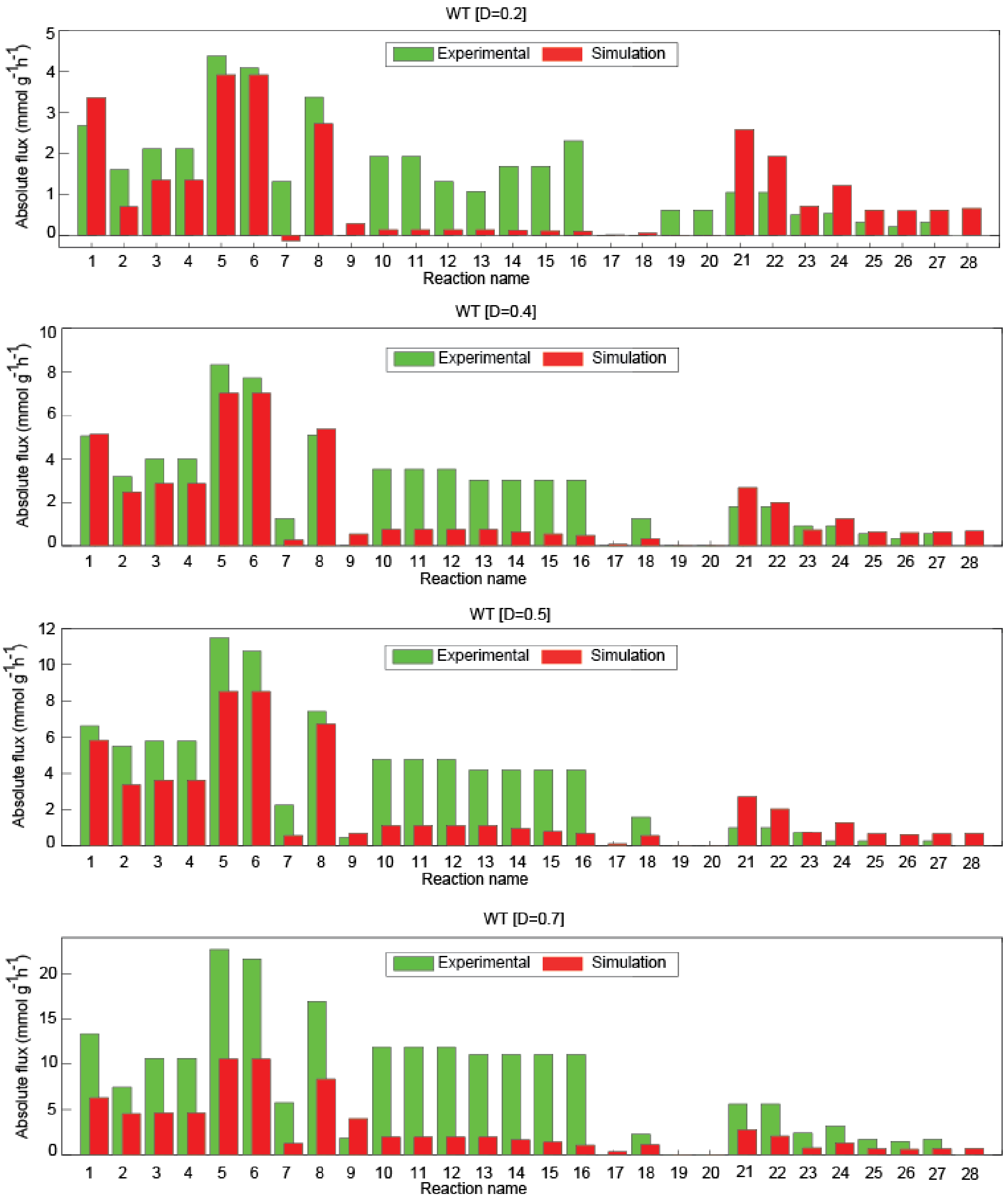

Figure S3: Comparison of the simulated flux with experimental flux [2] of WT at different dilution rates in a continuous culture.

The indexes of the reaction name indicate 1: v_Pts4, 2: vE_Pgi, 3: vE_Pfk - vE_Fbp, 4: vE_Fba, 5 : vE_Gapdh, 6: vE_Gapdh, 7: vE_Pyk - vE_Pps, 8: vE_Pdh, 9: vE_Ack - vE_Acs, 10: vE_Cs, 11: vE_Cs, 12: vE_Icdh, 13: vE_αkgdh, 14: vE_Sdh, 15: vE_Fum, 16: vE_Mdh, 17: vE_Mez, 18: vE_Ppc-vE_Pck, 19: vE_Icl, 20: vE_Ms, 21: vE_G6pdh, 22: vE_6Pgdh, 23: vE_R5pi, 24: vE_Ru5p, 25: vE_TktA, 26: vE_TktB, 27: vE_Tal, 28: vE_Edd.

A

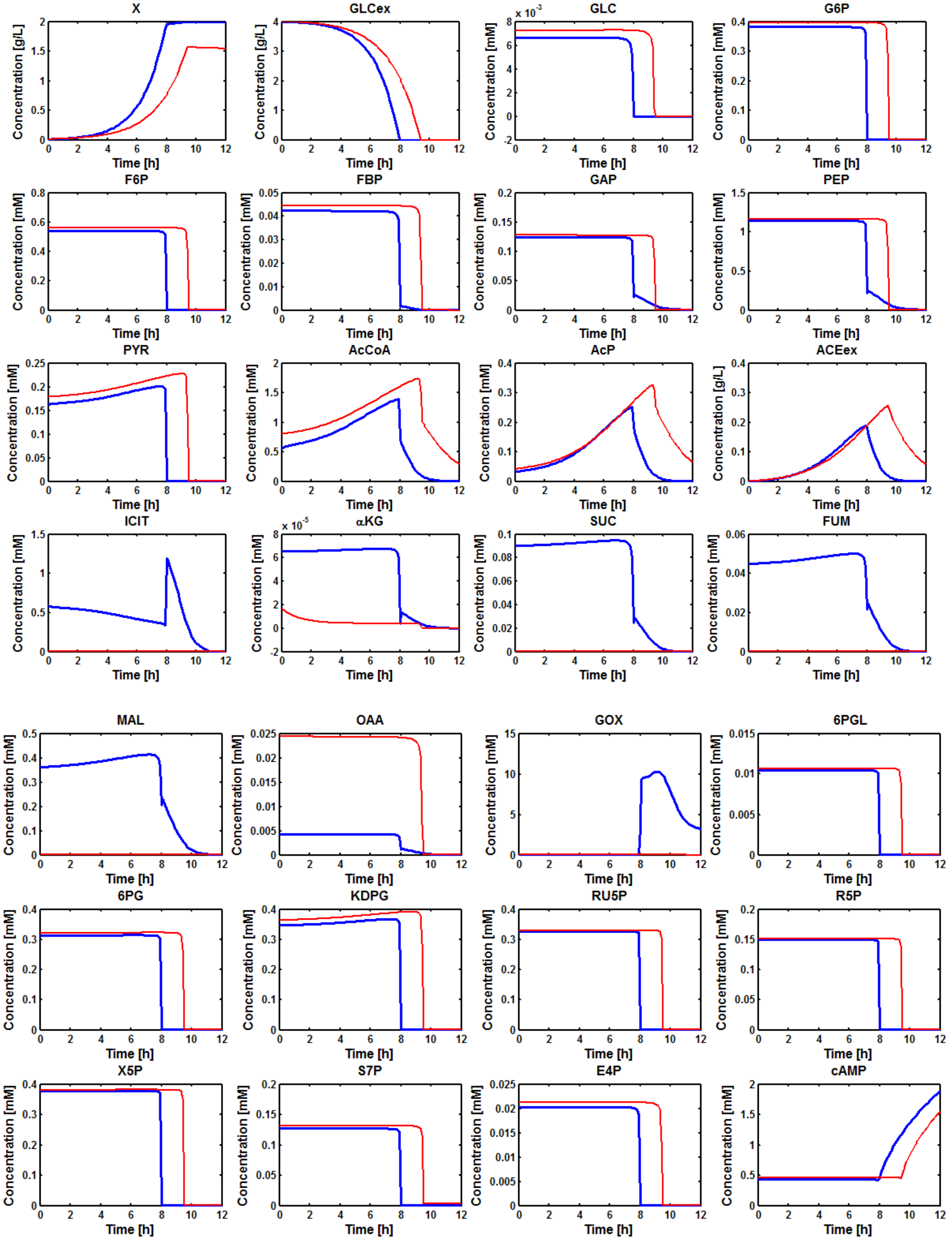
X
GLCex
GLC
G6P
F6P
FBP
GAP
PEP
PYR
AcCoA
AcP
ACEex
ICIT
αKG
SUC
FUM
MAL
OAA
GOX
6PGL
6PG
KDPG
RU5P
R5P
X5P
S7P
E4P
cAMP
Concentration [g/L]
Concentration [mM]
Time [h]

B

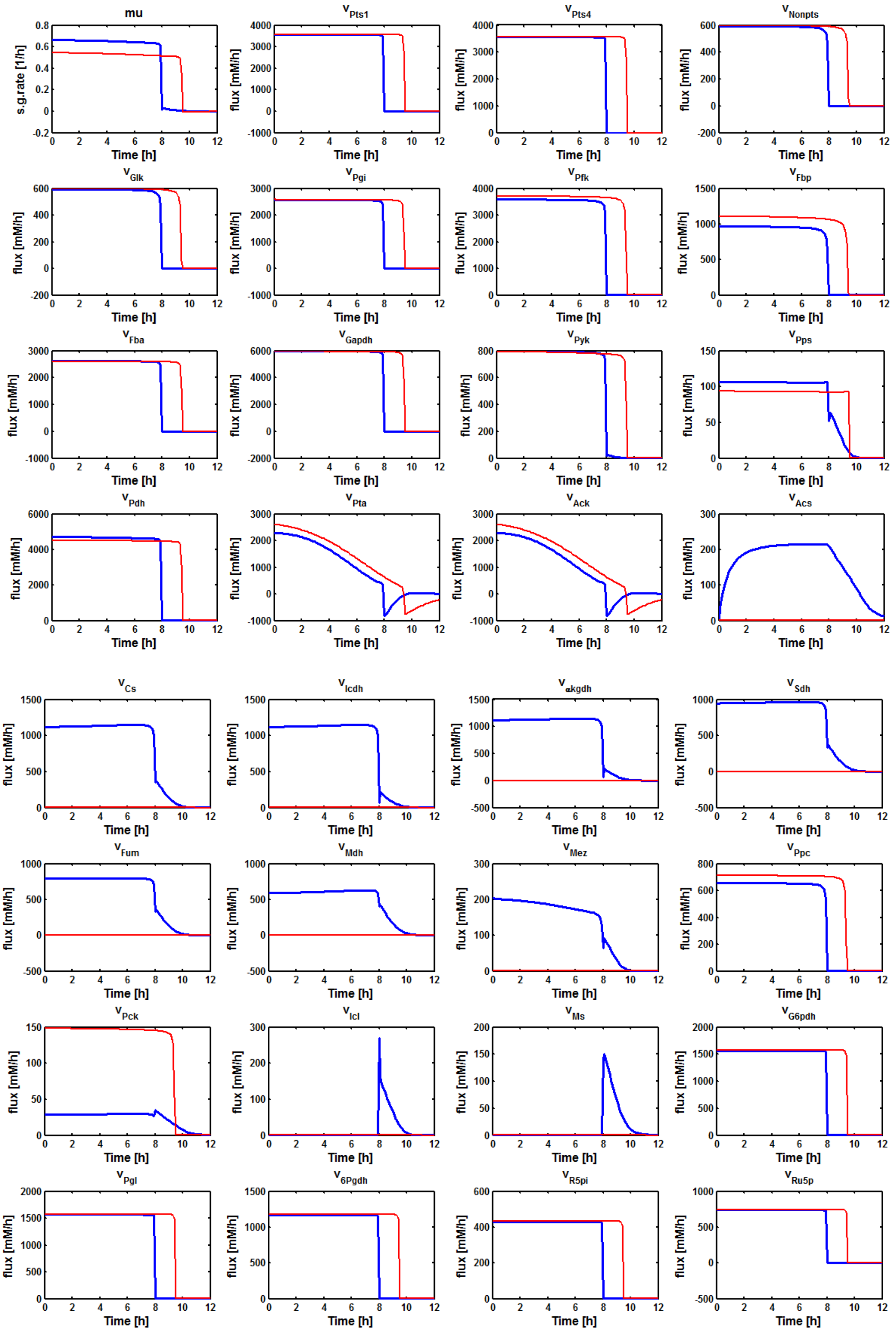
mu
s.g.rate [1/h]
Time [h]
flux [mM/h]
v Pts1
v Pts4
v Nonpts
v Glk
v Pgi
v Pfk
v Fbp
v Fba
v Gapdh
v Pyk
v Pps
v Pdh
v Pta
v Ack
v Acs
v Cs
v Icdh
v αkgdh
v Sdh
v Fum
v Mdh
v Mez
v Ppc
v Pck
v Icl
v Ms
v G6pdh
v Pgl
v 6Pgdh
v R5pi
v Ru5p

B (continue…)

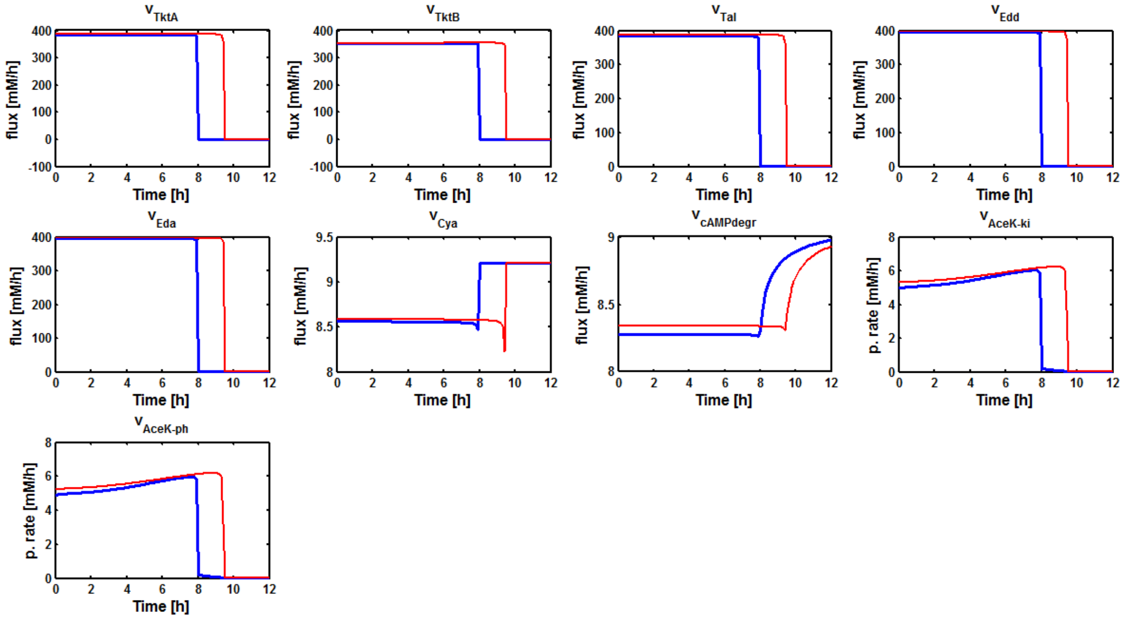

Figure S4: Effect of Crp regulation on dynamic behaviors.

The blue and red lines indicate WT and the virtual mutant lacking a Crp-cAMP complex, respectively.

A. Metabolite concentrations; **B.** Rates (Specific growth rate [s.g.rate]; flux; phosphorylation rate [p.rate]).

A

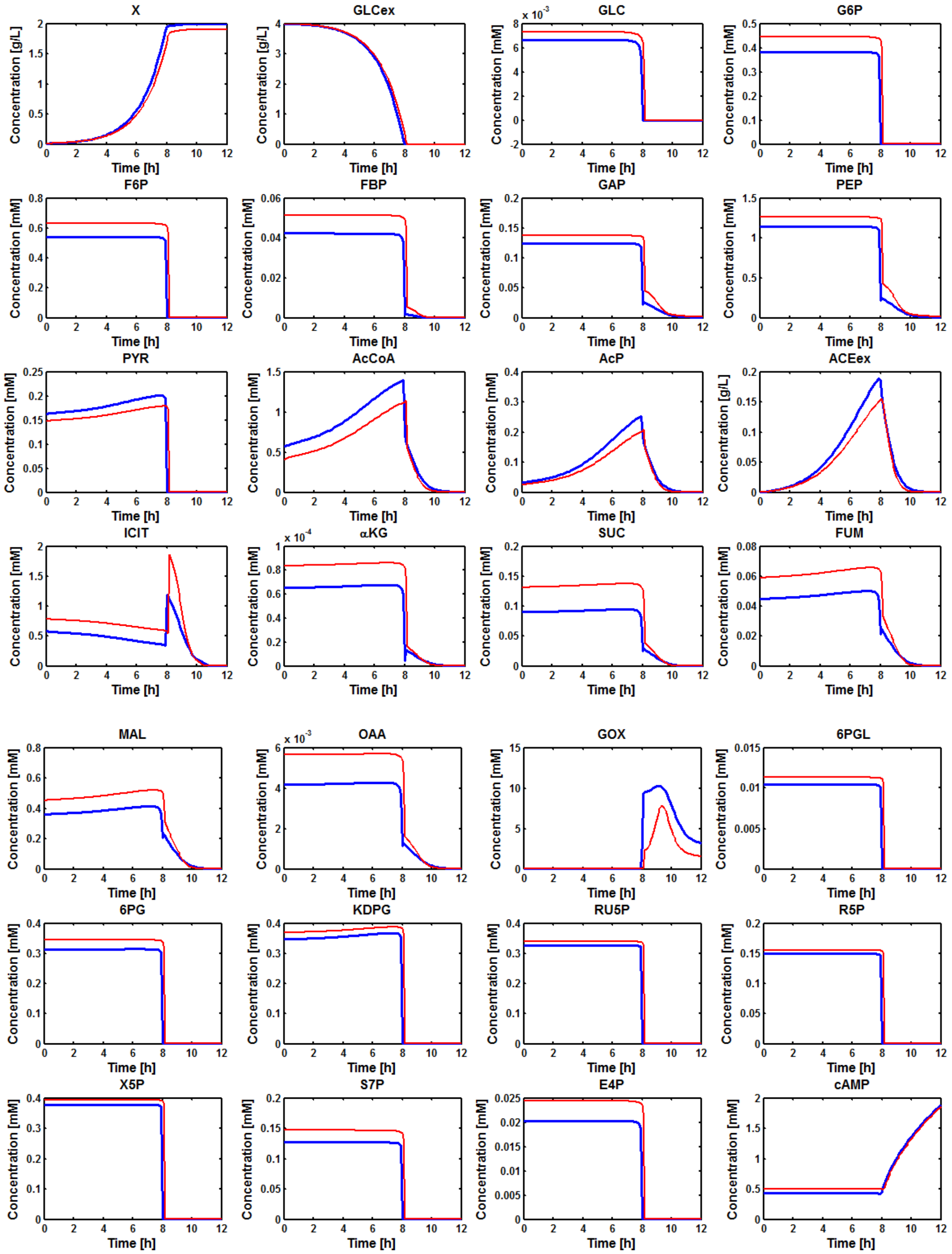
X
GLCex
GLC
G6P
F6P
FBP
GAP
PEP
PYR
AcCoA
AcP
ACEex
ICIT
αKG
SUC
FUM
MAL
OAA
GOX
6PGL
6PG
KDPG
RU5P
R5P
X5P
S7P
E4P
cAMP
Concentration [g/L]
Concentration [mM]
Time [h]

B

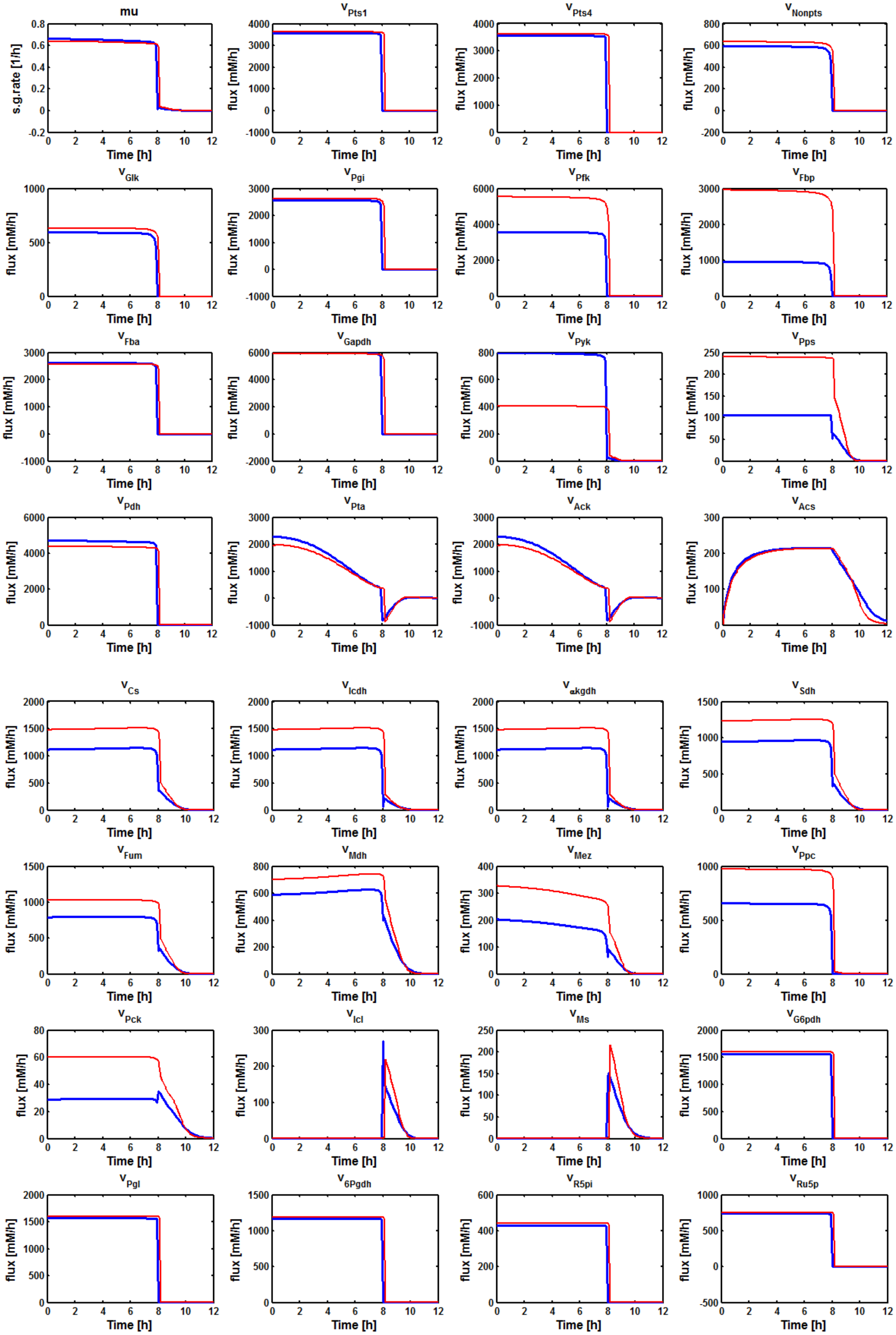

mu
s.g.rate [1/h]
Time [h]
flux [mM/h]
v_Pts1
v_Pts4
v_Nonpts
v_Glk
v_Pgi
v_Pfk
v_Fbp
v_Fba
v_Gapdh
v_Pyk
v_Pps
v_Pdh
v_Pta
v_Ack
v_Acs
v_Cs
v_Icdh
v_αkgdh
v_Sdh
v_Fum
v_Mdh
v_Mez
v_Ppc
v_Pck
v_Icl
v_Ms
v_G6pdh
v_Pgl
v_6Pgdh
v_R5pi
v_Ru5p

B (continue…)

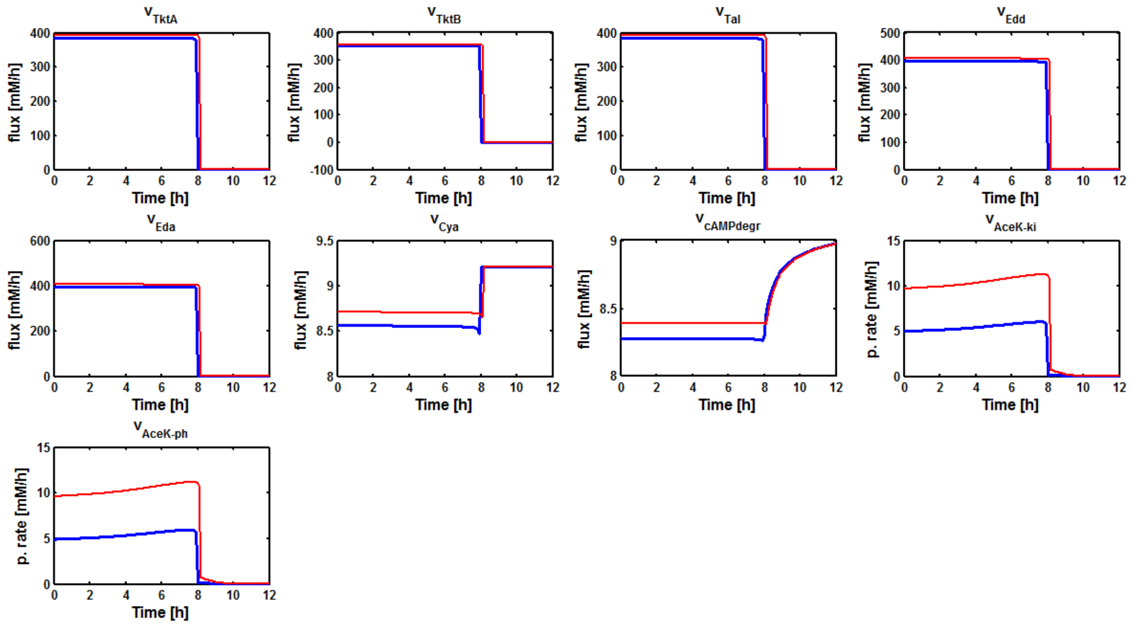

Figure S5: Effect of Cra regulation on dynamic behaviors.

The blue and red lines indicate WT and the virtual mutant lacking of a Cra-FBP complex, respectively.

A. Metabolite concentrations; **B.** Rates (Specific growth rate [s.g.rate]; flux; phosphorylation rate [p.rate]).

A

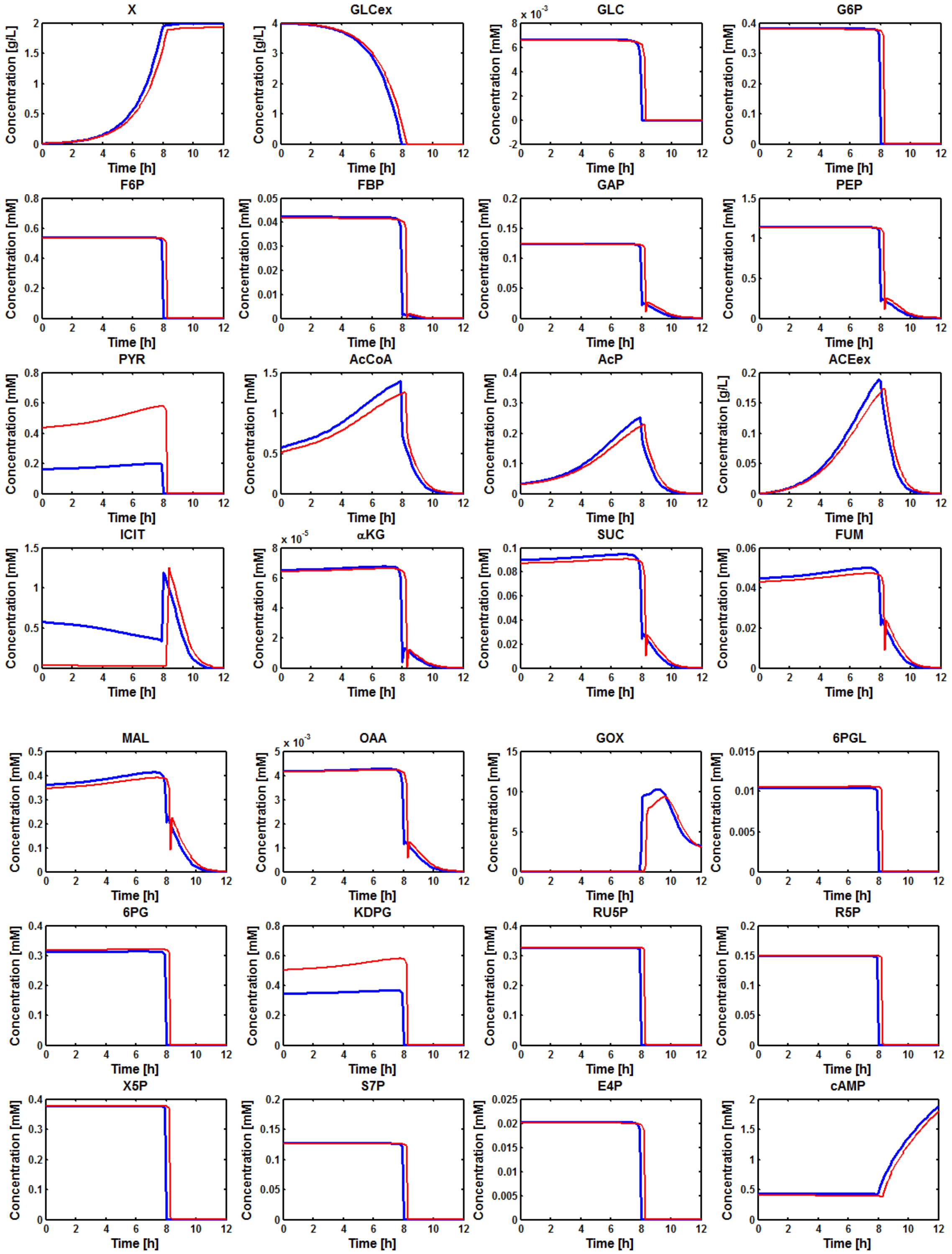
X
GLCex
GLC
G6P
F6P
FBP
GAP
PEP
PYR
AcCoA
AcP
ACEex
ICIT
αKG
SUC
FUM
MAL
OAA
GOX
6PGL
6PG
KDPG
RU5P
R5P
X5P
S7P
E4P
cAMP
Concentration [g/L]
Concentration [mM]
Time [h]
x 10^-3
x 10^-5

B

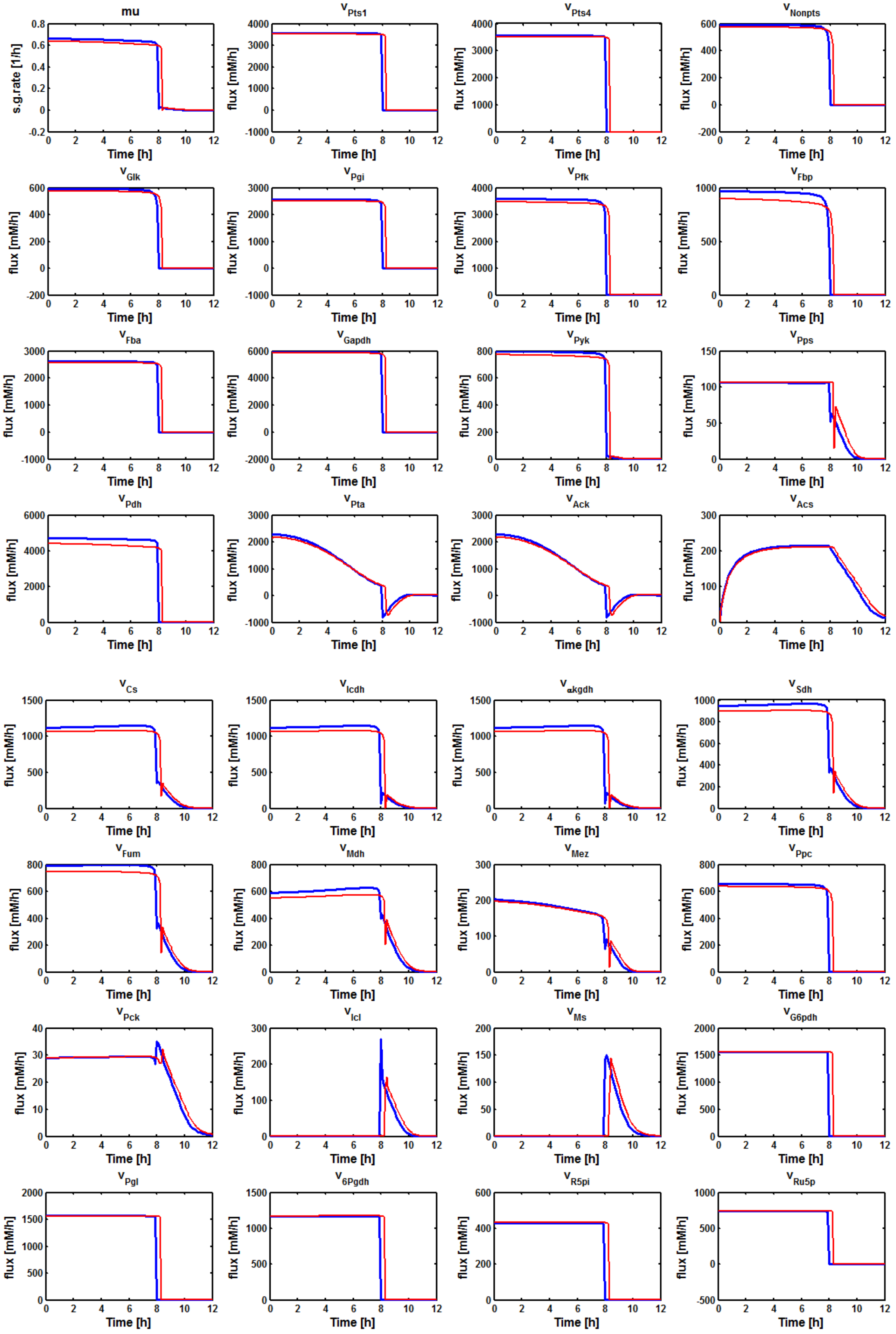
mu
s.g.rate [1/h]
flux [mM/h]
Time [h]
vPts1
vPts4
vNonpts
vGlk
vPgi
vPfk
vFbp
vFba
vGapdh
vPyk
vPps
vPdh
vPta
vAck
vAcs
vCs
vIcdh
vαkgdh
vSdh
vFum
vMdh
vMez
vPpc
vPck
vIcl
vMs
vG6pdh
vPgl
v6Pgdh
vR5pi
vRu5p

B (continue…)

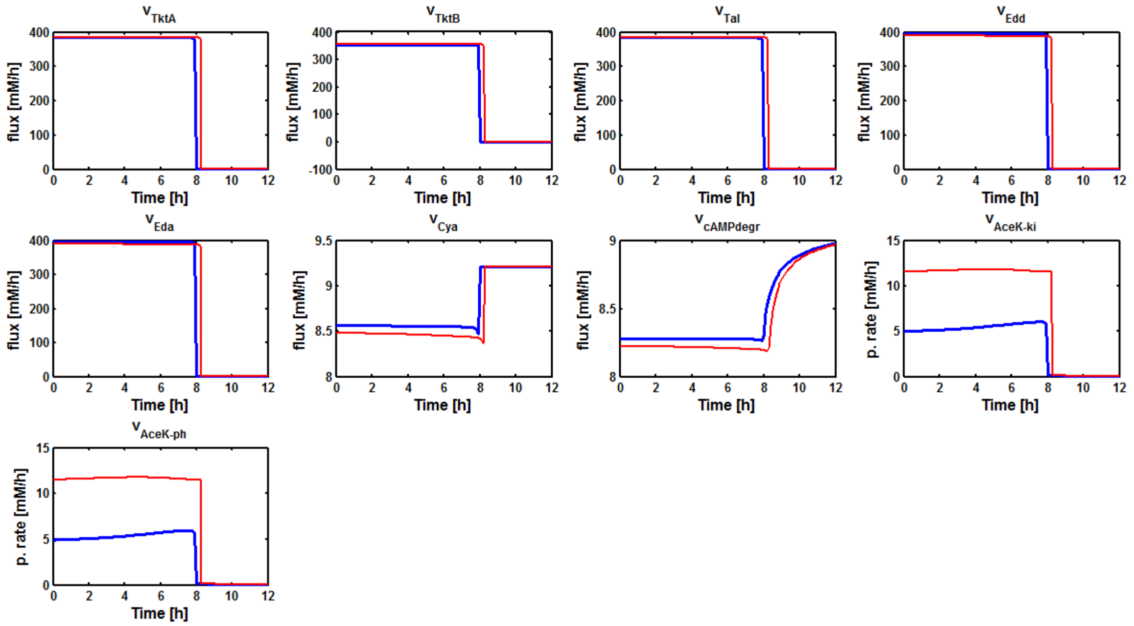

Figure S6: Effect of PdhR regulation on dynamic behaviors.

The blue and red lines indicate WT and the virtual mutant lacking a PdhR-PYR complex, respectively.

A. Metabolite concentrations; **B.** Rates (Specific growth rate [s.g.rate]; flux; phosphorylation rate [p.rate]).

A

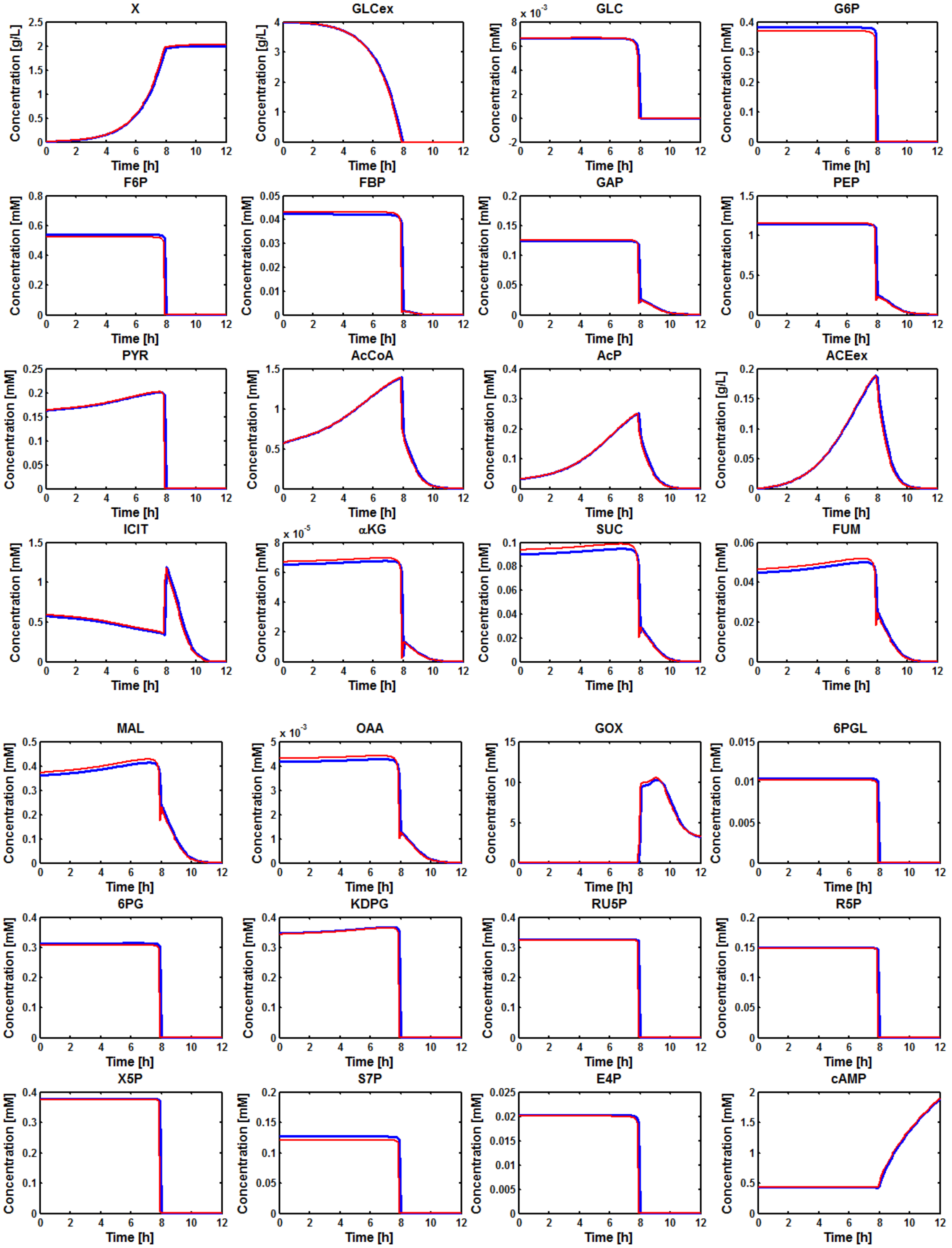
X
GLCex
GLC
G6P
F6P
FBP
GAP
PEP
PYR
AcCoA
AcP
ACEex
ICIT
αKG
SUC
FUM
MAL
OAA
GOX
6PGL
6PG
KDPG
RU5P
R5P
X5P
S7P
E4P
cAMP
Concentration [g/L]
Concentration [mM]
Time [h]

B

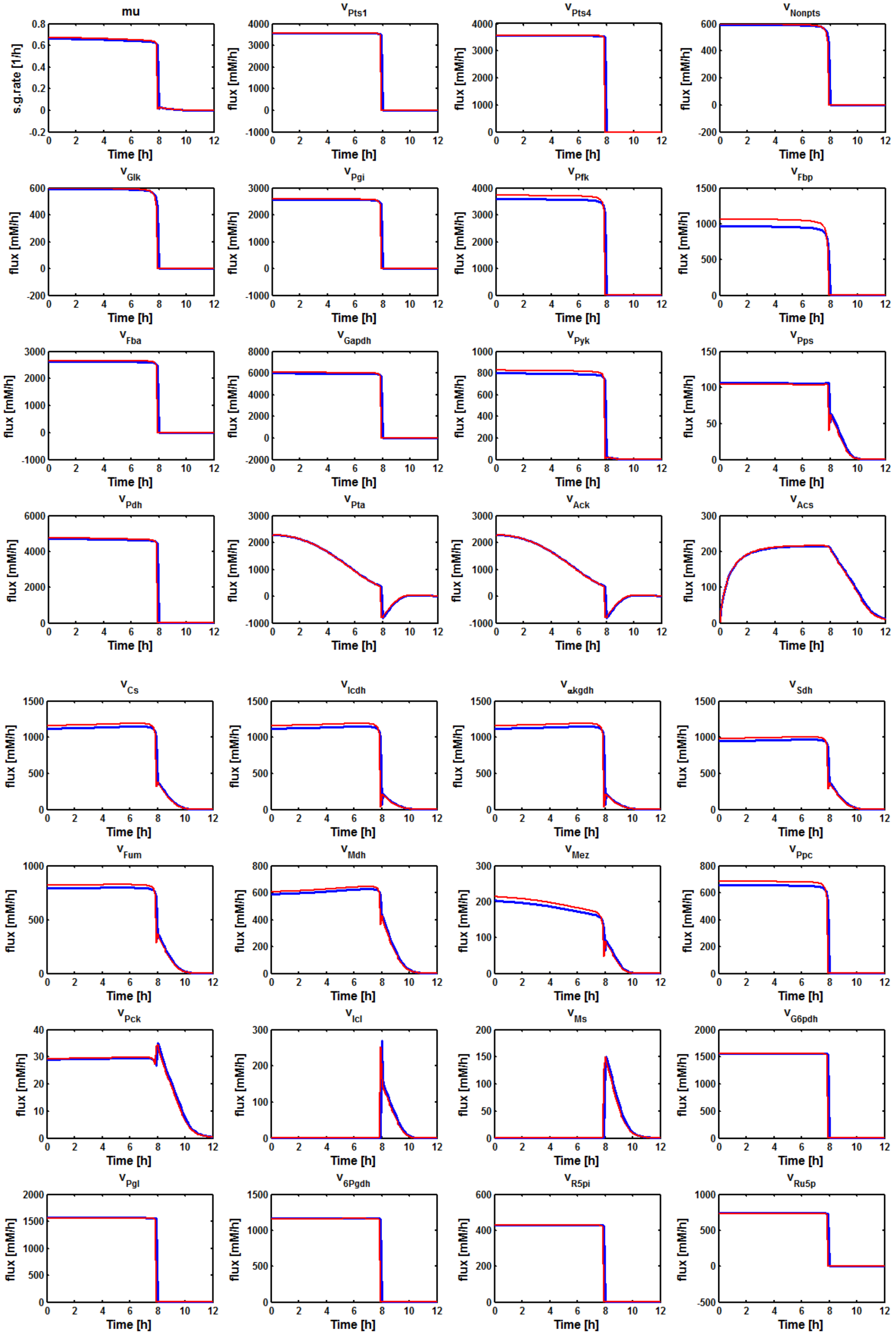
mu
s.g.rate [1/h]
Time [h]
v_{Pts1}
v_{Pts4}
v_{Nonpts}
v_{Glk}
v_{Pgi}
v_{Pfk}
v_{Fbp}
v_{Fba}
v_{Gapdh}
v_{Pyk}
v_{Pps}
v_{Pdh}
v_{Pta}
v_{Ack}
v_{Acs}
v_{Cs}
v_{Icdh}
$v_{\alpha kgdh}$
v_{Sdh}
v_{Fum}
v_{Mdh}
v_{Mez}
v_{Ppc}
v_{Pck}
v_{Icl}
v_{Ms}
v_{G6pdh}
v_{Pgl}
v_{6Pgdh}
v_{R5pi}
v_{Ru5p}
flux [mM/h]

B (continue…)

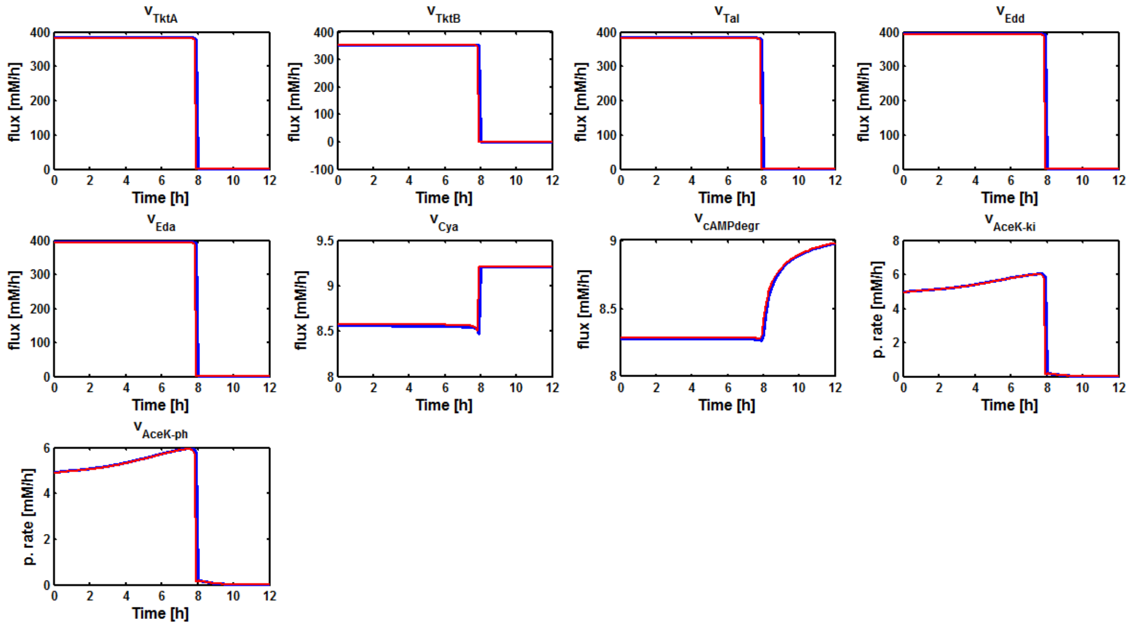

Figure S7: Effect of allosteric regulation of Pfk on dynamic behaviors.

The blue and red lines indicates WT and the virtual mutant lacking the allosteric regulations via Pfk, respectively.

A. Metabolite concentrations; **B.** Rates (Specific growth rate [s.g.rate]; flux; phosphorylation rate [p.rate]).

A

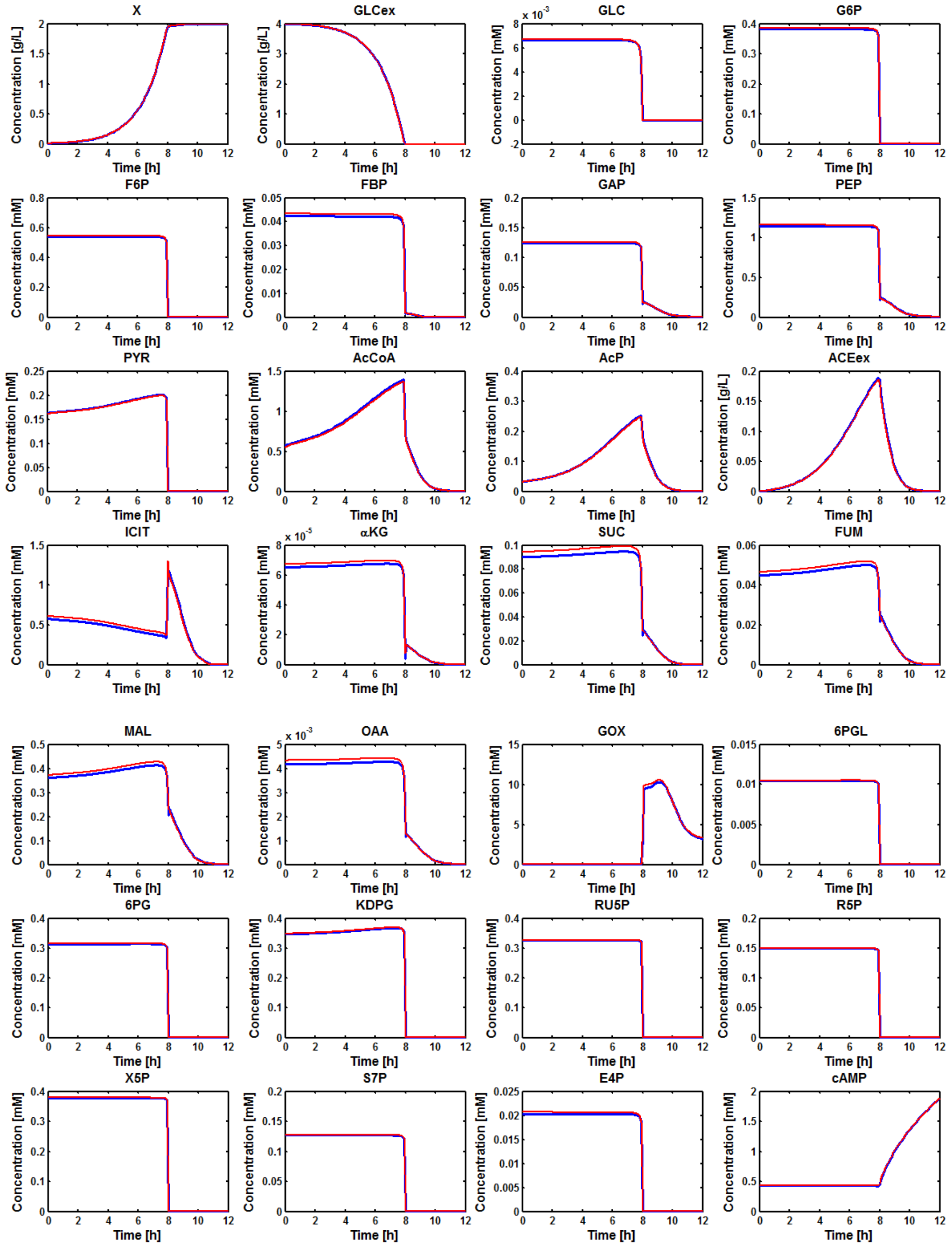

X
GLCex
GLC
G6P
F6P
FBP
GAP
PEP
PYR
AcCoA
AcP
ACEex
ICIT
αKG
SUC
FUM
MAL
OAA
GOX
6PGL
6PG
KDPG
RU5P
R5P
X5P
S7P
E4P
cAMP
Concentration [g/L]
Concentration [mM]
Time [h]

B

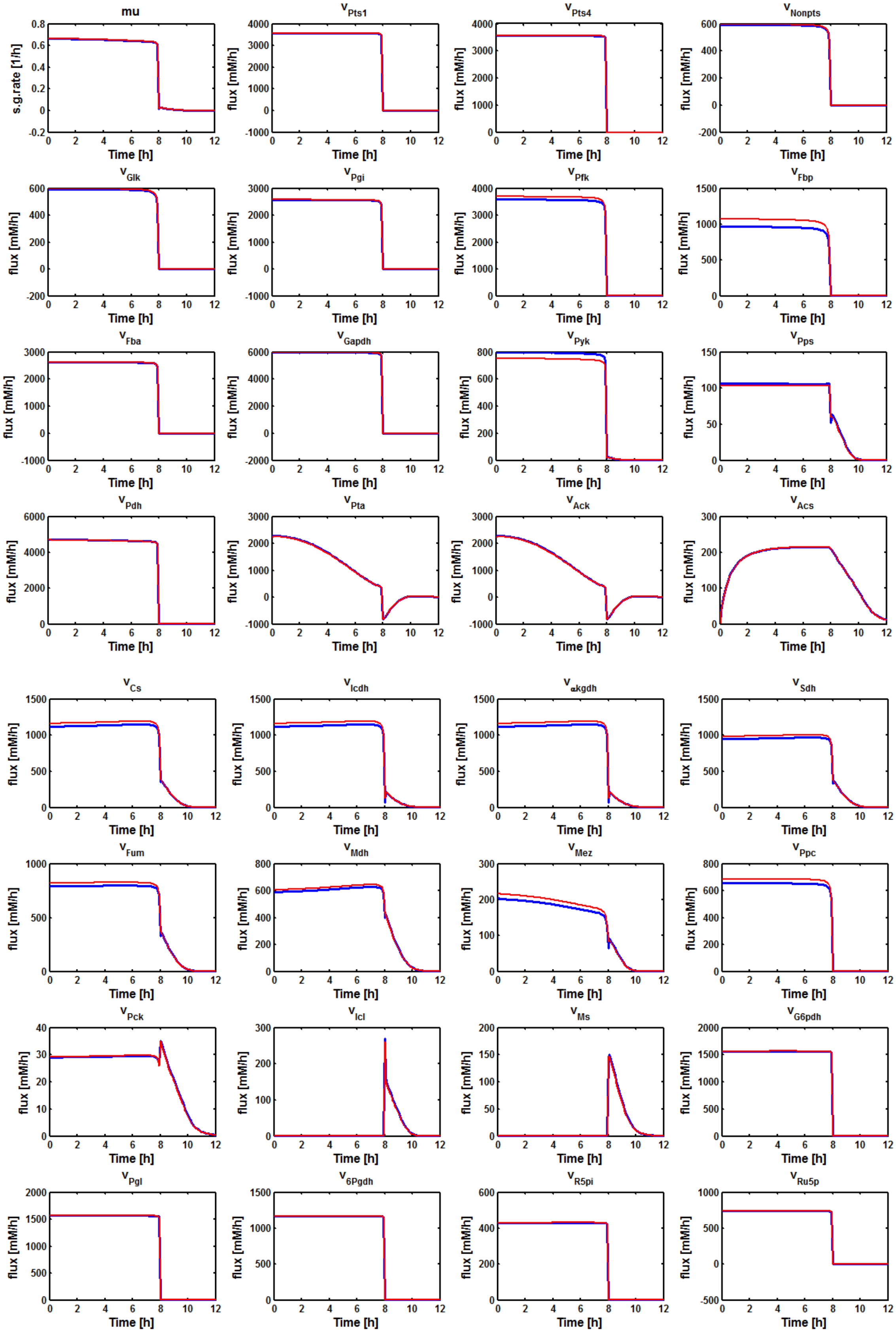

mu
s.g.rate [1/h]
flux [mM/h]
Time [h]
VPts1
VPts4
VNonpts
VGlk
VPgi
VPfk
VFbp
VFba
VGapdh
VPyk
VPps
VPdh
VPta
VAck
VAcs
VCs
VIcdh
Vαkgdh
VSdh
VFum
VMdh
VMez
VPpc
VPck
VIcl
VMs
VG6pdh
VPgl
V6Pgdh
VR5pi
VRu5p

B (continue…)

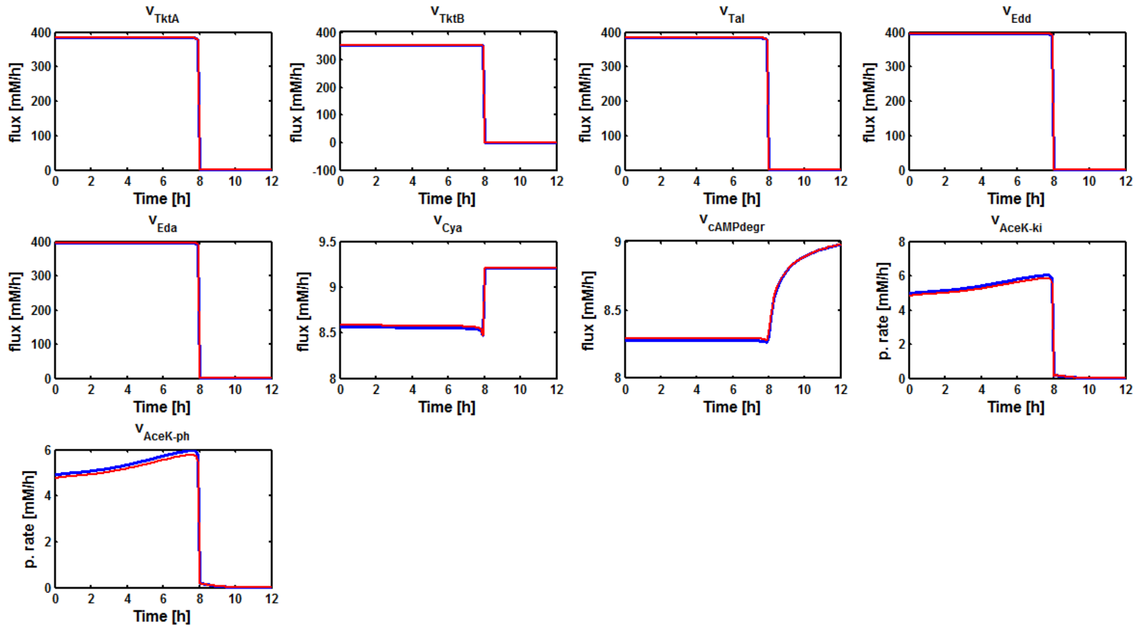

Figure S8: Effect of allosteric regulation of Pyk on dynamic behaviors.

The blue and red lines indicate WT and the virtual mutant lacking the allosteric regulation of Pyk.

A. Metabolite concentrations; **B.** Rates (Specific growth rate [s.g.rate]; flux; phosphorylation rate [p.rate]).

A

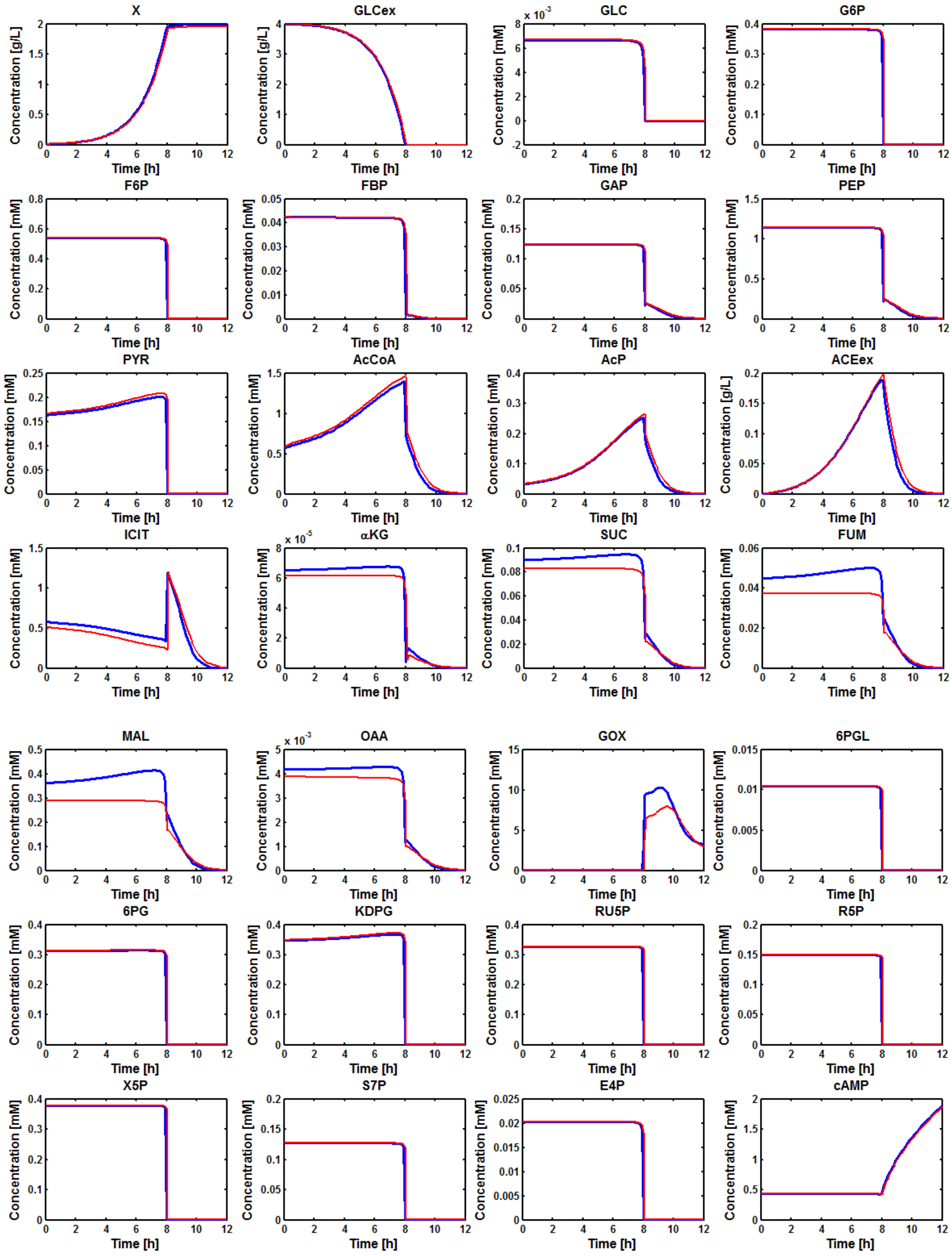
X
GLCex
GLC
G6P
F6P
FBP
GAP
PEP
PYR
AcCoA
AcP
ACEex
ICIT
αKG
SUC
FUM
MAL
OAA
GOX
6PGL
6PG
KDPG
RU5P
R5P
X5P
S7P
E4P
cAMP
Concentration [g/L]
Concentration [mM]
Time [h]
x 10^-3
x 10^-5

B

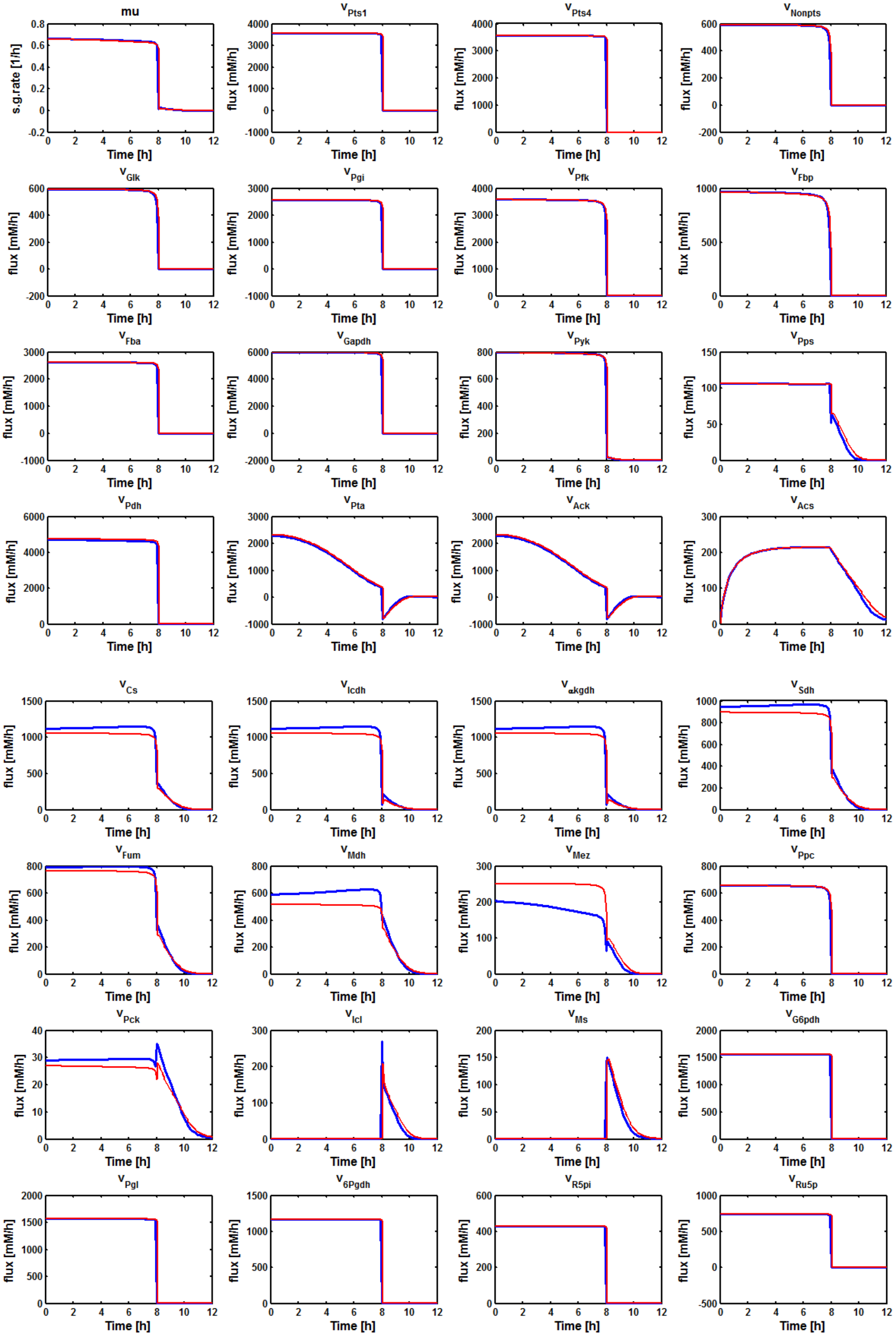

mu
v_{Pts1}
v_{Pts4}
v_{Nonpts}
v_{Glk}
v_{Pgi}
v_{Pfk}
v_{Fbp}
v_{Fba}
v_{Gapdh}
v_{Pyk}
v_{Pps}
v_{Pdh}
v_{Pta}
v_{Ack}
v_{Acs}
v_{Cs}
v_{Icdh}
$v_{\alpha kgdh}$
v_{Sdh}
v_{Fum}
v_{Mdh}
v_{Mez}
v_{Ppc}
v_{Pck}
v_{Icl}
v_{Ms}
v_{G6pdh}
v_{Pgl}
v_{6Pgdh}
v_{R5pi}
v_{Ru5p}
s.g.rate [1/h]
flux [mM/h]
Time [h]

B (continue…)

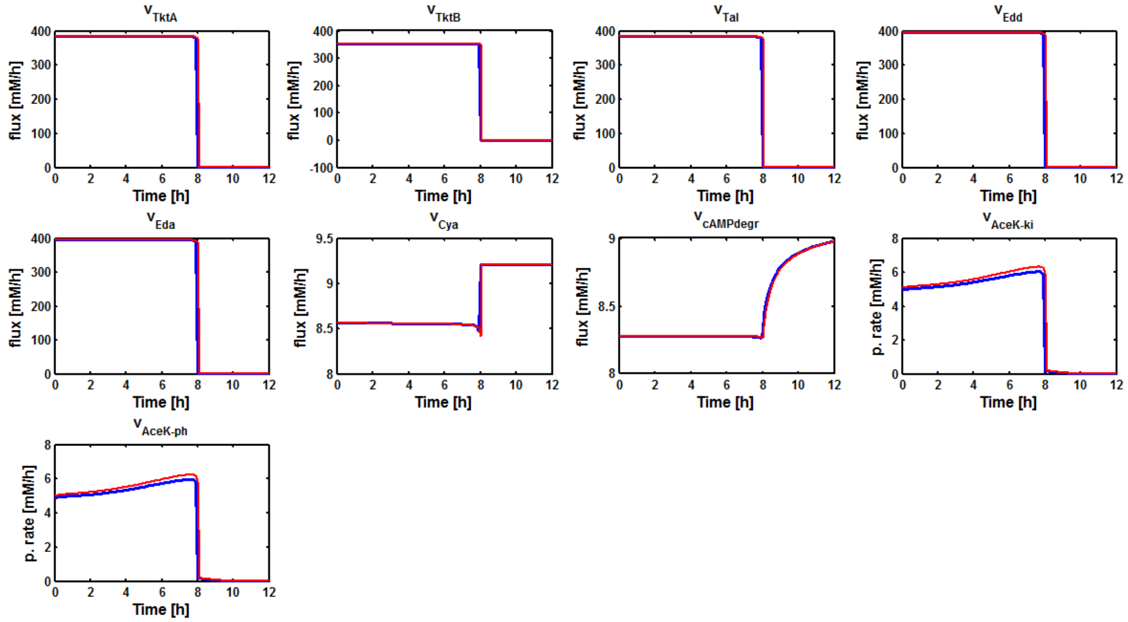

Figure S9: Effect of allosteric regulation of Mez on dynamic behaviors.

The blue and red lines indicate WT and the virtual mutant lacking the allosteric regulation of Mez.

A. Metabolite concentrations; **B.** Rates (Specific growth rate [s.g.rate]; flux; phosphorylation rate [p.rate]).

A

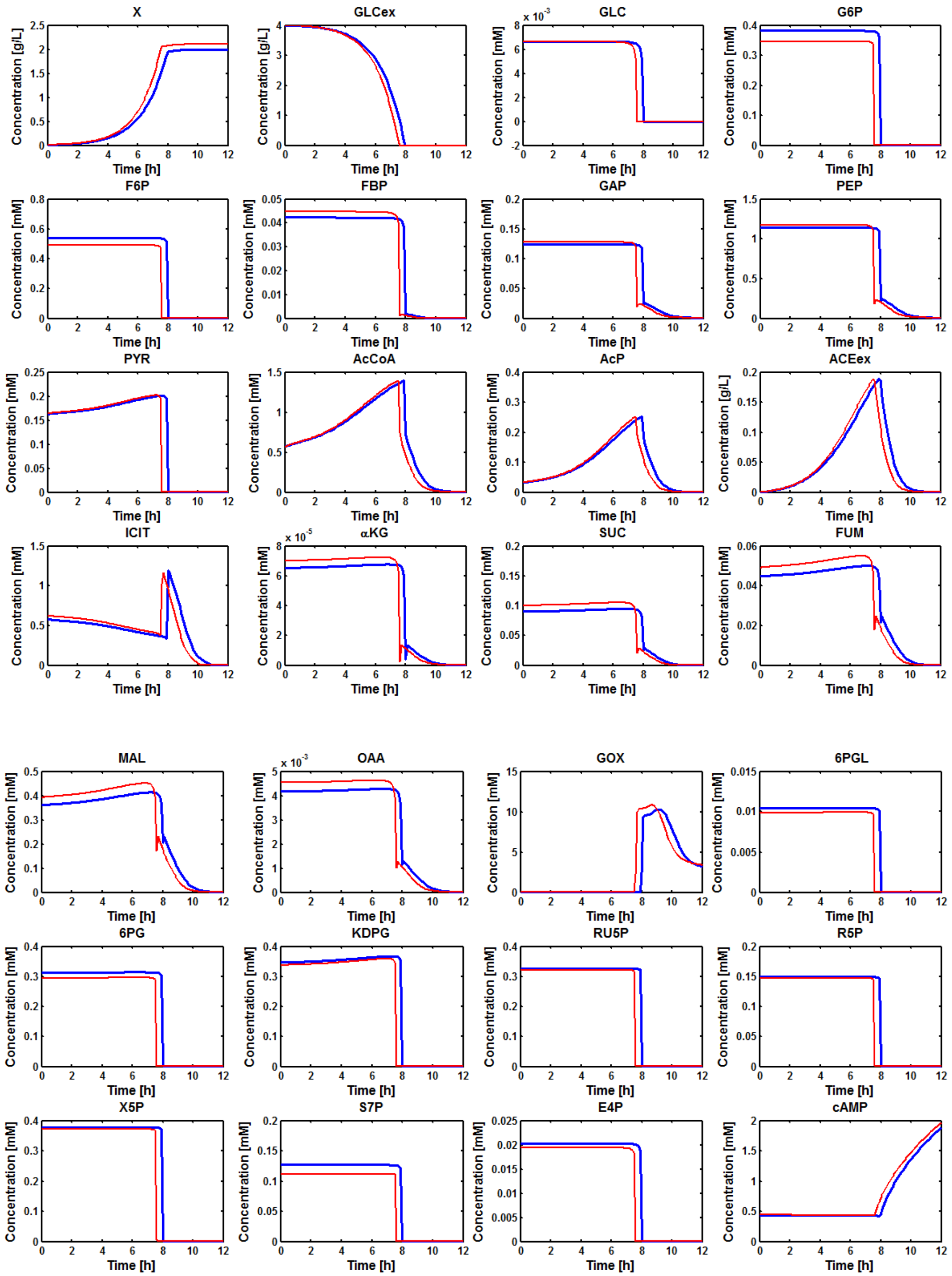
X
GLCex
GLC
G6P
F6P
FBP
GAP
PEP
PYR
AcCoA
AcP
ACEex
ICIT
αKG
SUC
FUM
MAL
OAA
GOX
6PGL
6PG
KDPG
RU5P
R5P
X5P
S7P
E4P
cAMP
Concentration [g/L]
Concentration [mM]
Time [h]
x 10^-3
x 10^-5

B

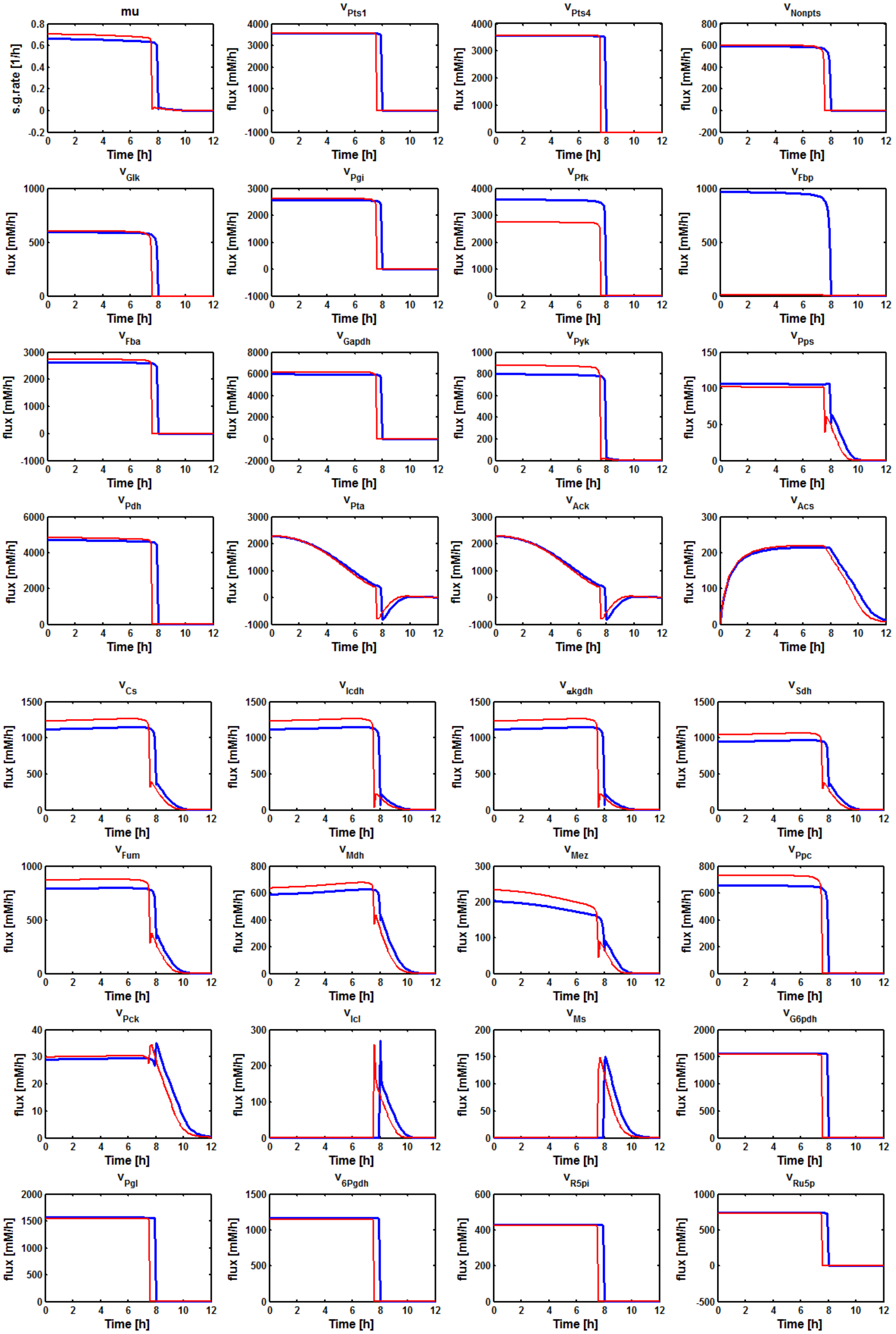

mu
s.g.rate [1/h]
Time [h]
v Pts1
v Pts4
v Nonpts
v Glk
v Pgi
v Pfk
v Fbp
v Fba
v Gapdh
v Pyk
v Pps
v Pdh
v Pta
v Ack
v Acs
v Cs
v Icdh
v αkgdh
v Sdh
v Fum
v Mdh
v Mez
v Ppc
v Pck
v Icl
v Ms
v G6pdh
v Pgl
v 6Pgdh
v R5pi
v Ru5p
flux [mM/h]

B (continue…)

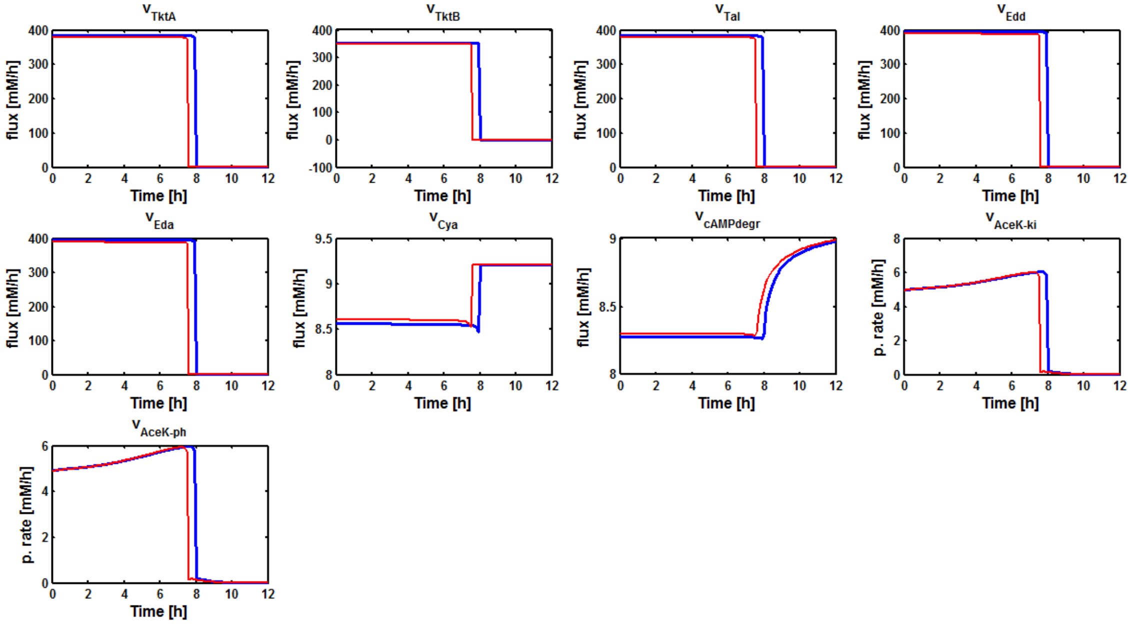

Figure S10: Effect of allosteric regulation of Fbp on dynamic behaviors.

The blue and red lines indicate WT and the virtual mutant lacking the allosteric regulation of Fbp.

A. Metabolite concentrations; **B.** Rates (Specific growth rate [s.g.rate]; flux; phosphorylation rate [p.rate]).

A

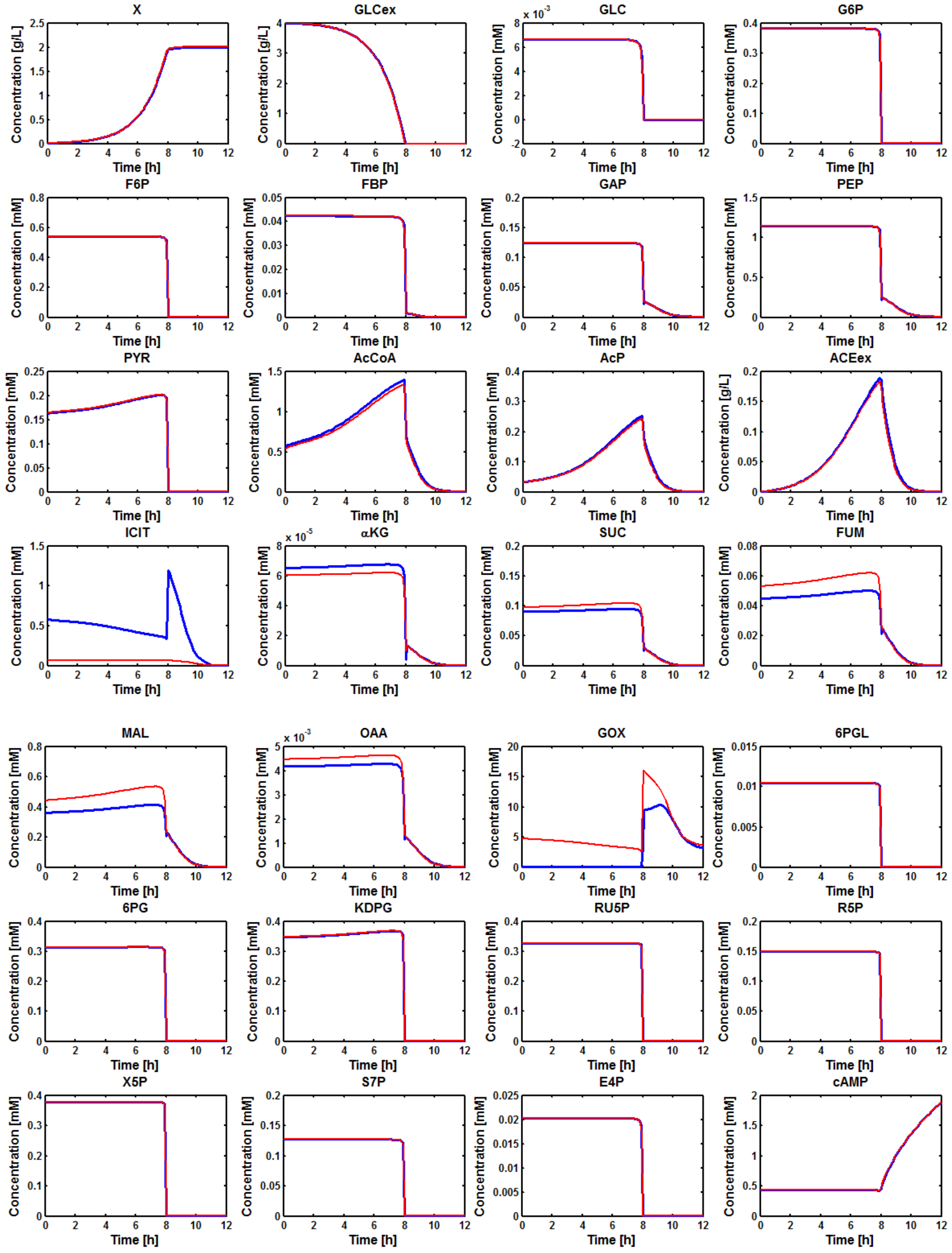

X
GLCex
GLC
G6P
F6P
FBP
GAP
PEP
PYR
AcCoA
AcP
ACEex
ICIT
αKG
SUC
FUM
MAL
OAA
GOX
6PGL
6PG
KDPG
RU5P
R5P
X5P
S7P
E4P
cAMP
Concentration [g/L]
Concentration [mM]
Time [h]

B

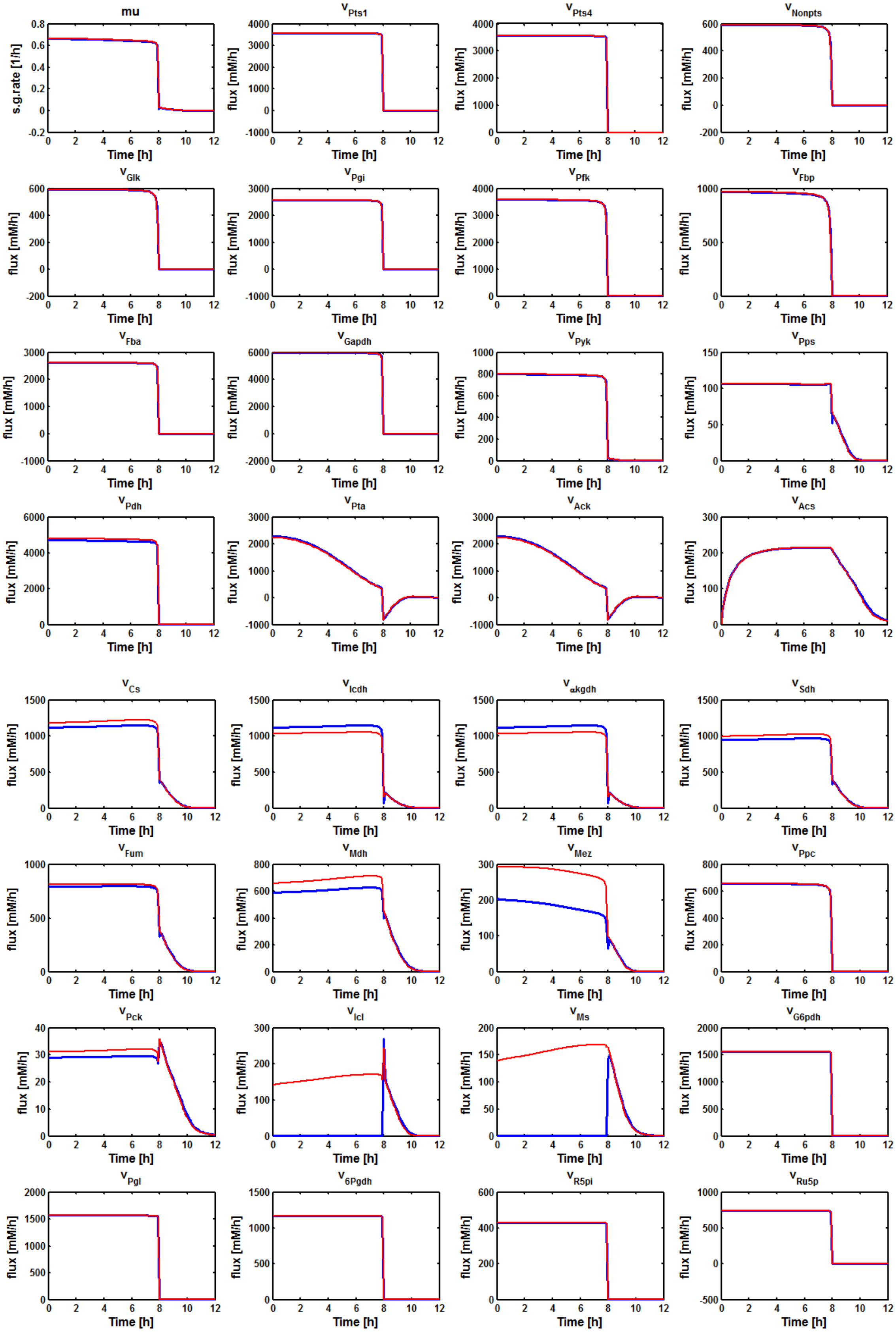
mu
s.g.rate [1/h]
vPts1
vPts4
vNonpts
vGlk
vPgi
vPfk
vFbp
vFba
vGapdh
vPyk
vPps
vPdh
vPta
vAck
vAcs
vCs
vIcdh
vαkgdh
vSdh
vFum
vMdh
vMez
vPpc
vPck
vIcl
vMs
vG6pdh
vPgl
v6Pgdh
vR5pi
vRu5p
flux [mM/h]
Time [h]

B (continue…)

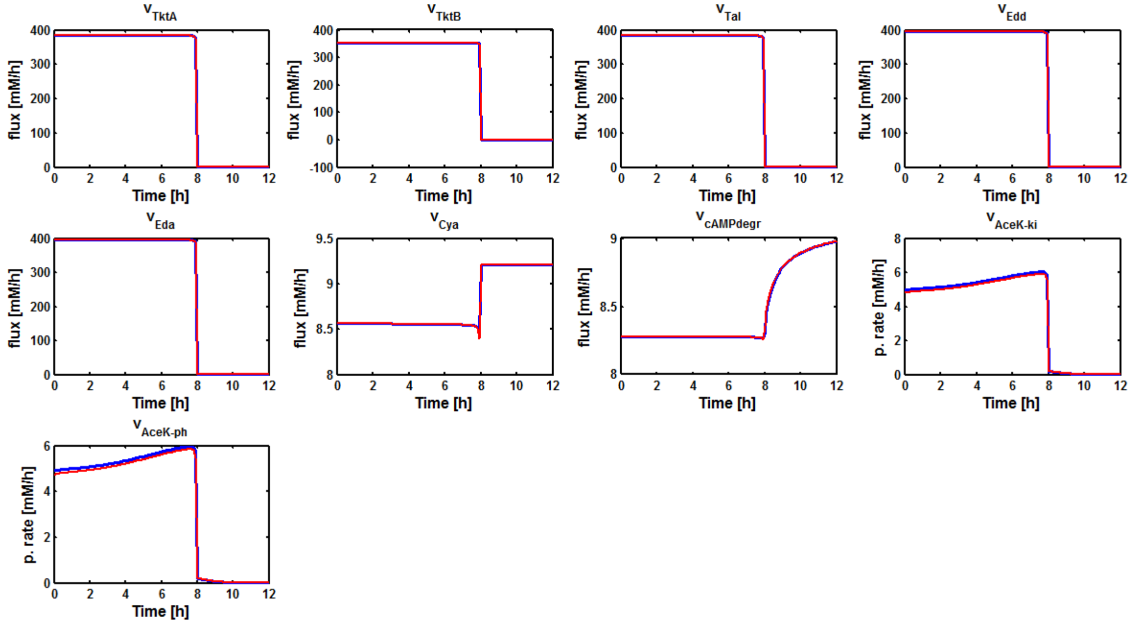

Figure S11: Effect of allosteric regulation of Icl on dynamic behaviors.

The blue and red lines indicate WT and the virtual mutant lacking the allosteric regulation of Icl.

A. Metabolite concentrations; **B.** Rates (Specific growth rate [s.g.rate]; flux; phosphorylation rate [p.rate]).

A

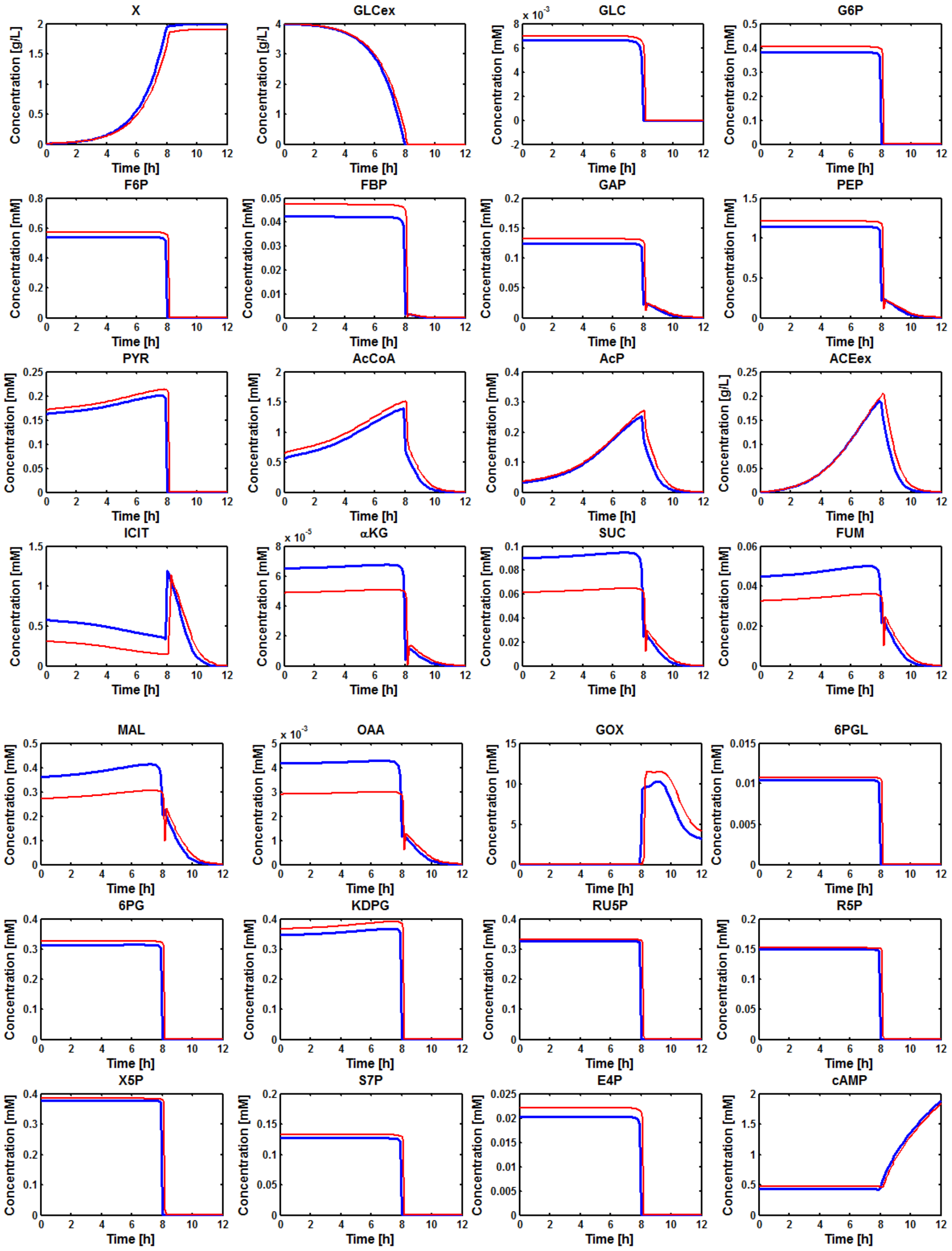
X
GLCex
GLC
G6P
F6P
FBP
GAP
PEP
PYR
AcCoA
AcP
ACEex
ICIT
αKG
SUC
FUM
MAL
OAA
GOX
6PGL
6PG
KDPG
RU5P
R5P
X5P
S7P
E4P
cAMP
x 10^-3
x 10^-5
Concentration [g/L]
Concentration [mM]
Time [h]

B

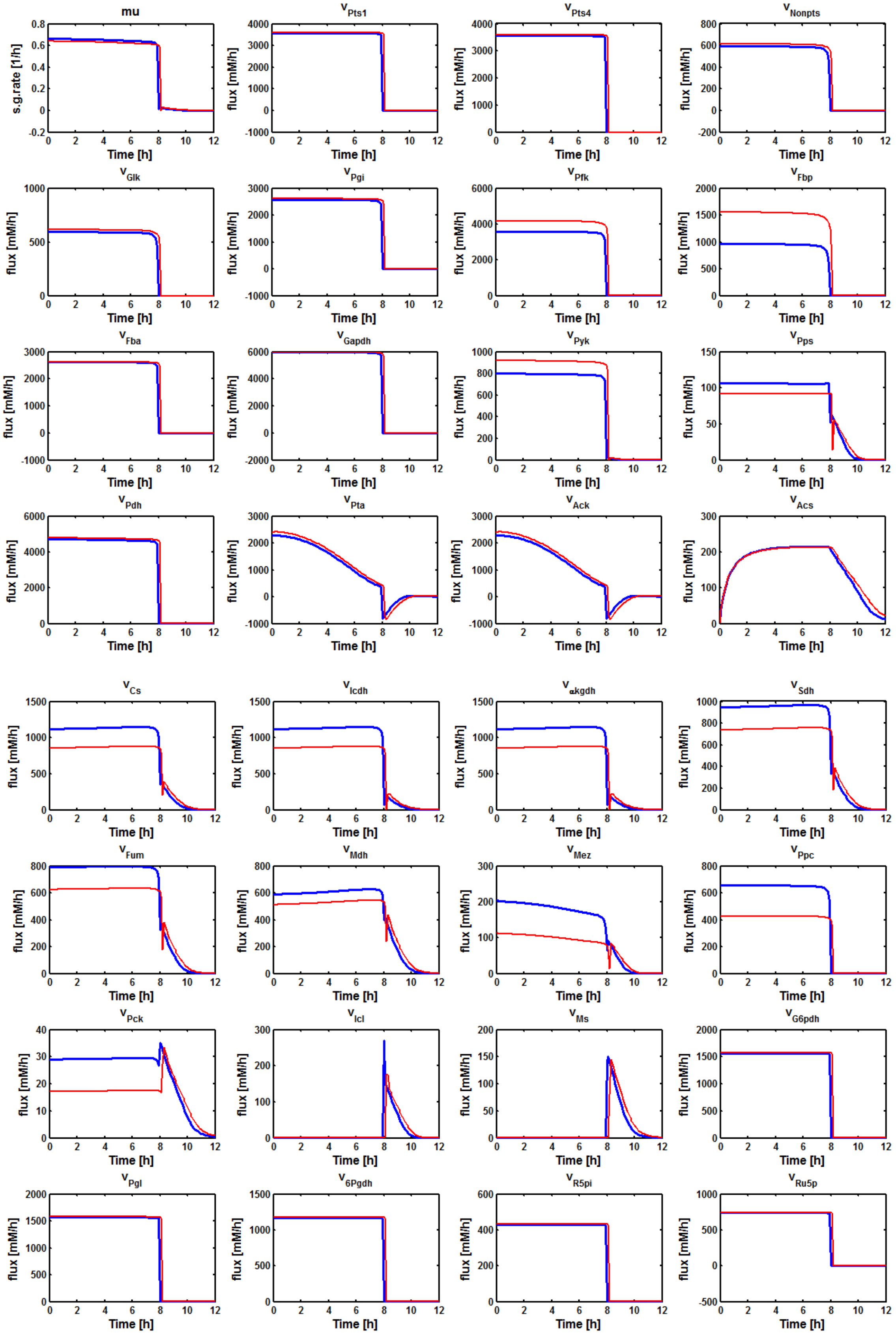
mu
s.g.rate [1/h]
Time [h]
flux [mM/h]
v Pts1
v Pts4
v Nonpts
v Glk
v Pgi
v Pfk
v Fbp
v Fba
v Gapdh
v Pyk
v Pps
v Pdh
v Pta
v Ack
v Acs
v Cs
v Icdh
v αkgdh
v Sdh
v Fum
v Mdh
v Mez
v Ppc
v Pck
v Icl
v Ms
v G6pdh
v Pgl
v 6Pgdh
v R5pi
v Ru5p

B (continue…)

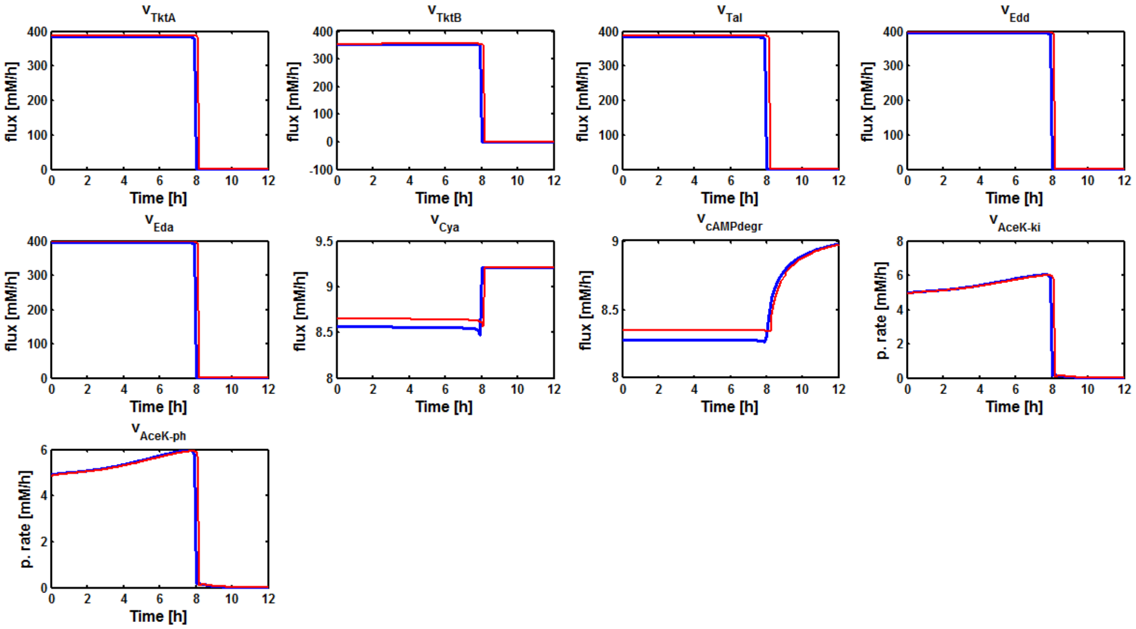

Figure S12: Effect of allosteric regulation of Ppc on dynamic behaviors.

The blue and red lines indicate WT and the virtual mutant lacking the allosteric regulation of Ppc.

A. Metabolite concentrations; **B.** Rates (Specific growth rate [s.g.rate]; flux; phosphorylation rate [p.rate]).

References

1. Toya Y, Ishii N, Nakahigashi K, Hirasawa T, Soga T, Tomita M, Shimizu K: **13C-metabolic flux analysis for batch culture of *Escherichia coli* and its *Pyk* and *Pgi* gene knockout mutants based on mass isotopomer distribution of intracellular metabolites.** *Biotechnol Prog* 2010, **26:**975-992.
2. Ishii N, Nakahigashi K, Baba T, Robert M, Soga T, Kanai A, Hirasawa T, Naba M, Hirai K, Hoque A, et al: **Multiple high-throughput analyses monitor the response of *E. coli* to perturbations.** *Science* 2007, **316:**593-597.
