# Supplementary material for: Development of an accurate kinetic model for the central carbon metabolism of Escherichia coli
Source: Microb Cell Fact. 2016 Jun 21;15:112. doi: 10.1186/s12934-016-0511-x (PMC4915146; doi:10.1186/s12934-016-0511-x)
Supplement: Supplementary file 2 — 10.1186/s12934-016-0511-x Comparison of our kinetic model with other existing models. [file 12934_2016_511_MOESM4_ESM.pdf]

Details of the parameter estimation method

1. Experimental data used for parameter estimation:

Batch culture time-course data for WT, Δ*pykF* and Δ*pgi* from [1]

- Cell concentration and extracellular glucose and acetate: Figure 2a and 2b of [1]
- Intracellular metabolite concentrations: Figure 5a of [1]
- Flux distribution data: Supplementary Table S-IV to S-VII of [1]

Batch culture time-course data for Δ*ppc* from [2]

- Cell concentration and extracellular glucose and acetate: Figure 4A of [2]

2. Methods of parameter estimation

2.1 Constrained optimization

Parameter estimation problem can be formulated as a constrained optimization problem:

$$\begin{aligned} &\text{Minimize}\, f(\mathbf{p}) \\ &\text{Subject to } \mathbf{g}(\mathbf{p}) \le \mathbf{0} \end{aligned}, \qquad \text{(S1)}$$

where $\mathbf{p} = (p_1, p_2, \ldots, p_n)$ is the model parameter vector. f is the objective function which evaluates the deviation between estimated and reference parameters. $\mathbf{g} = (g_1, g_2, \ldots, g_m)$ is the constraint function vector. f is given by

$$f(\mathbf{p}) = \lambda_1 \sum_{p_i \in Class\ I} \left| \log_{10} \frac{p_i}{p_i{*}} \right| + \lambda_2 \sum_{p_i \in Class\ II} \left| \log_{10} \frac{p_i}{p_i{*}} \right| + \lambda_3 \sum_{p_i \in Class\ III} \left| \log_{10} \frac{p_i}{p_i{*}} \right|. \qquad \text{(S2)}$$

We categorized the model parameters into three classes according to reliability of their reference values. Class I includes the parameters for which measured values are available. Class II includes the parameters for which estimations are available from references. Class III includes the parameters for which no information is available. λs are the penalty weights ($\lambda_1 = 100$, $\lambda_2 = 10$, $\lambda_3 = 0$). If parameter values are changed from reliable reference values, large penalties are imposed. **g** consists of nine constraint functions:

g_1 for fitting to experimental data (WT)

g_2 for fitting to experimental data (Δ*pykF*)

g_3 for fitting to experimental data (Δ*pgi*)

g_4 for fitting to experimental data (Δ*ppc*)

g_5 for reasonable molecular concentrations and fluxes (WT)

g_6 for reasonable molecular concentrations and fluxes (Δ*pykF*)

g_7 for reasonable molecular concentrations and fluxes (Δ*pgi*)

g_8 for reasonable molecular concentrations and fluxes (Δ*ppc*)

g_9 for reasonable activity changes of EIIA and transcription factors (WT).Detailed calculation procedures for gs are shown in the following sections. In order to solve the above constrained optimization problem, we employed a real-coded genetic algorithm (GA) with UNDX [3] as a crossover method and MGG [4] as a generation alternation method. We paralleled the GA using MPI. The GA was run on the super computer Shirokane3 provided by the Human Genome Center at The University of Tokyo.

2.2 Constraints for fitting to experimental data

g_1-g_4 are the constraints for the deviation between model predictions and experimental data. g_1 is given by

$$g_1 = h_{1,1} + h_{1,2} + h_{1,3} - AE \,, \tag{S3}$$

where AE is the allowable error and set to 0.18 in this study. $h_{1,1}$ evaluates fitting to the cell concentration, extracellular glucose and acetate concentrations (Figure 2a and b of [1]):

$$h_{1,1} = \sum_{t=\{0,1,\ldots,10\}} \left(\frac{X(t) - X^*(t)}{\max_{t=\{0,1,\ldots,10\}}\left(X^*(t)\right)} \right)^2 + \sum_{t=\{0,1,\ldots,10\}} \left(\frac{GLC^{ex}(t) - GLC^{ex*}(t)}{\max_{t=\{0,1,\ldots,10\}}\left(GLC^{ex*}(t)\right)} \right)^2 + \sum_{t=\{0,1,\ldots,10\}} \left(\frac{ACE^{ex}(t) - ACE^{ex*}(t)}{\max_{t=\{0,1,\ldots,10\}}\left(ACE^{ex*}(t)\right)} \right)^2 , \tag{S4}$$

where t is the time (in hour). X, GLC^{ex} and ACE^{ex} show the predicted cell concentration, predicted extracellular glucose and acetate concentrations, respectively. The variables with an asterisk show experimental data.

$h_{1,2}$ evaluates fitting to the intracellular metabolite concentrations reported in Figure 5a of [1]:

$$h_{1,2}(x_i) = \sum_{t=\{4,5,6,7,8,8.5,9,10\}} \sum_{x_i \in LIST1} \left(\frac{x_i(t) - x_i^*(t)}{\max_{t=\{4,5,6,7,8,8.5,9,10\}}\left(x_i^*(t)\right)} \right)^2 , \tag{S5}$$

$$LIST1 = \{PYR, MAL, PEP, GAP, Ru5P, R5P, G6P, 6PG, S7P, FBP\} , \tag{S6}$$

where x_i shows the ith molecular concentration.

$h_{1,3}$ evaluates fitting to the flux distribution reported in Supplementary Table S-IV to S-VII of [1].

$$h_{1,3}(x_i) = \sum_{t=\{4,5,6,7\}} \sum_{x_i \in LIST2} \left(\frac{v_i(t)}{v_{Pts4}(t)} - \frac{v_i^*(t)}{v_{Pts4}^*(t)} \right)^2 + \sum_{t=\{8,8.5,9,10\}} \sum_{x_i \in LIST2} \left(\frac{v_i(t)}{v_{Ack}(t)} - \frac{v_i^*(t)}{v_{Ack}^*(t)} \right)^2 , \tag{S7}$$

$$\begin{aligned} LIST2 = \{ & v_{Pts4}, v_{E,Pgi}, v_{E,Pfk}, v_{E,Fba}, v_{E,Gapdh}, v_{E,Pyk}, v_{E,Pdh}, v_{E,Ack}, v_{E,G6pdh}, v_{E,6gdh}, \\ & v_{E,Ru5p}, v_{E,R5pi}, v_{E,TktA}, v_{E,Tal}, v_{E,TktB}, v_{E,Cs}, v_{E,Icdh}, v_{E,akgdh}, v_{E,Sdh}, v_{E,Fum}, \\ & v_{E,Mdh}, v_{E,Ppc}, v_{E,Mez}, v_{E,Icl}, v_{E,Ms}, v_{E,Kdpg} \} \end{aligned} , \tag{S8}$$

where v_i shows the ith flux. g_2 and g_3 are calculated in the same way as g_1, where g_2 is for $\Delta pykF$ and g_3 for Δpgi. Since intracellular metabolite concentration and flux distribution data are lacking for Δppc, g_4 evaluates only fitting to data for cell concentration and extracellular glucose and acetate, i.e. $g_4 = h_{4,1} - AE$.

2.3 Constraints for concentrations and fluxes

All of molecular concentrations and fluxes should be within a biologically reasonable range. g_5 is employed for this propose and given by

$$g_5 = \sum_{t=\{0,0.1,\ldots,12\}} \left(\sum_{x_i \,\in\, Intracellular\ Metabolites} h_{4,1}\left(x_i(t)\right) + \sum_{x_i \,\in\, Proteins} h_{4,2}\left(x_i(t)\right) + \sum_{All\ v_i} h_{4,3}\left(v_i(t)\right) \right), \tag{S9}$$

where x_i and v_i are the ith molecular concentration and the ith flux, respectively. $h_{5,1}$, $h_{5,2}$, and $h_{5,3}$ are given by

$$h_{5,1}\left(x_i(t)\right)=\begin{cases} x_i(t)^2 & (x_i(t)<0) \\ 0 & (0\le x_i(t)\le ub_{metabolite}) \\ \left(\dfrac{x_i(t)-ub_{metabolite}}{ub_{metabolite}}\right)^2 & (ub_{metabolite}<x_i(t)) \end{cases}, \tag{S10}$$

$$h_{5,2}\left(x_i(t)\right)=\begin{cases} x_i(t)^2 & (x_i(t)<0) \\ 0 & (0\le x_i(t)\le ub_{protein}) \\ \left(\dfrac{x_i(t)-ub_{protein}}{ub_{protein}}\right)^2 & (ub_{protein}<x_i(t)) \end{cases}, \tag{S11}$$

$$h_{5,3}\left(v_i(t)\right)=\begin{cases} 0 & (\left|v_i(t)\right|\le ub_{flux}) \\ \left(\dfrac{\left|v_i(t)\right|-ub_{flux}}{ub_{flux}}\right)^2 & (ub_{flux}<\left|v_i(t)\right|) \end{cases}. \tag{S12}$$

$ub_{metabolite}$, $ub_{protein}$ and ub_{flux} are the upper bounds and set to 20 mM, 0.2 mM, 6000 mM/h, respectively. g_6, g_7, and g_8 are calculated in the same way as g_5, where g_6 is for Δ*pykF*, g_7 for Δ*pgi*, and g_8 for Δ*ppc*.

2.4 Constraints for transcription factors

It is known that the activities of EIIA and transcription factors change in response to the carbon source shift from glucose to acetate. g_7 is employed to reproduce those experimental observations:

$$g_9=\sum_{i=\{EIIA,Crp,Cra,PdhR\}} h_{9,1}\left(Variation_i\right). \tag{S13}$$

$h_{9,1}$ is given by

$$h_{9,1}\left(Variation_i\right)=\begin{cases} \left(\dfrac{Variation_i-lb_{Variation}}{lb_{Variation}}\right)^2 & (Variation_i<lb_{Variation}) \\ 0 & (lb_{Variation}\le Variation_i) \end{cases}, \tag{S14}$$

where $lb_{Variation}$ is the lower bound and set to 0.5. $Variation_i$ is given by

$$\begin{aligned} Variation_{EIIA}=&\max_{t=\{0,0.1,\ldots,12\}}\left(\frac{EIIAP(t)}{EIIAP(t)+EIIA(t)}\right) \\ &-\min_{t=\{0,0.1,\ldots,12\}}\left(\frac{EIIAP(t)}{EIIAP(t)+EIIA(t)}\right), \end{aligned} \tag{S15}$$

$$\begin{aligned} Variation_{Crp}=&\max_{t=\{0,0.1,\ldots,12\}}\left(\frac{CrpcAMP(t)}{CrpcAMP(t)+Crp(t)}\right) \\ &-\min_{t=\{0,0.1,\ldots,12\}}\left(\frac{CrpcAMP(t)}{CrpcAMP(t)+Crp(t)}\right), \end{aligned} \tag{S16}$$

$$
\begin{aligned}
Variation_{Cra} = &\max_{t=\{0,0.1,...,12\}}\left(\frac{CraFBP(t)}{CraFBP(t)+Cra(t)}\right) \\
&-\min_{t=\{0,0.1,...,12\}}\left(\frac{CraFBP(t)}{CraFBP(t)+Cra(t)}\right),
\end{aligned}
\qquad (S17)
$$

$$
\begin{aligned}
Variation_{PdhR} = &\max_{t=\{0,0.1,...,12\}}\left(\frac{PdhRPYR(t)}{PdhRPYR(t)+PdhR(t)}\right) \\
&-\min_{t=\{0,0.1,...,12\}}\left(\frac{PdhRPYR(t)}{PdhRPYR(t)+PdhR(t)}\right).
\end{aligned}
\qquad (S18)
$$

References

1. Toya Y, Ishii N, Nakahigashi K, Hirasawa T, Soga T, Tomita M, Shimizu K: **13C-metabolic flux analysis for batch culture of Escherichia coli and its Pyk and Pgi gene knockout mutants based on mass isotopomer distribution of intracellular metabolites**. *Biotechnol Prog* 2010, **26**(4):975-992.
2. Abdul Kadir TA, Mannan AA, Kierzek AM, McFadden J, Shimizu K: **Modeling and simulation of the main metabolism in Escherichia coli and its several single-gene knockout mutants with experimental verification**. *Microb Cell Fact* 2010, **9**(1):88.
3. Ono I, Kobayashi S: **A real-coded genetic algorithm for function optimization using unimordal normal distribution crossover**. *Proc of 7th Int Conf on Genetic Algorithms* 1997:246-253.
4. Satoh H, Yamamura M, Kobayashi S: **Minimal generation gap model for GAs considering both exploration and exploitation**. *Proc of Int Conf on Fuzzy Logic, Neural Networks and Soft Computing* 1997:494-497.
