# Supplementary material for: Development of an accurate kinetic model for the central carbon metabolism of Escherichia coli
Source: Microb Cell Fact. 2016 Jun 21;15:112. doi: 10.1186/s12934-016-0511-x (PMC4915146; doi:10.1186/s12934-016-0511-x)
Supplement: Supplementary file 3 — 10.1186/s12934-016-0511-x Details of the kinetic model. [file 12934_2016_511_MOESM2_ESM.pdf]

Comparison of our kinetic model with other existing models

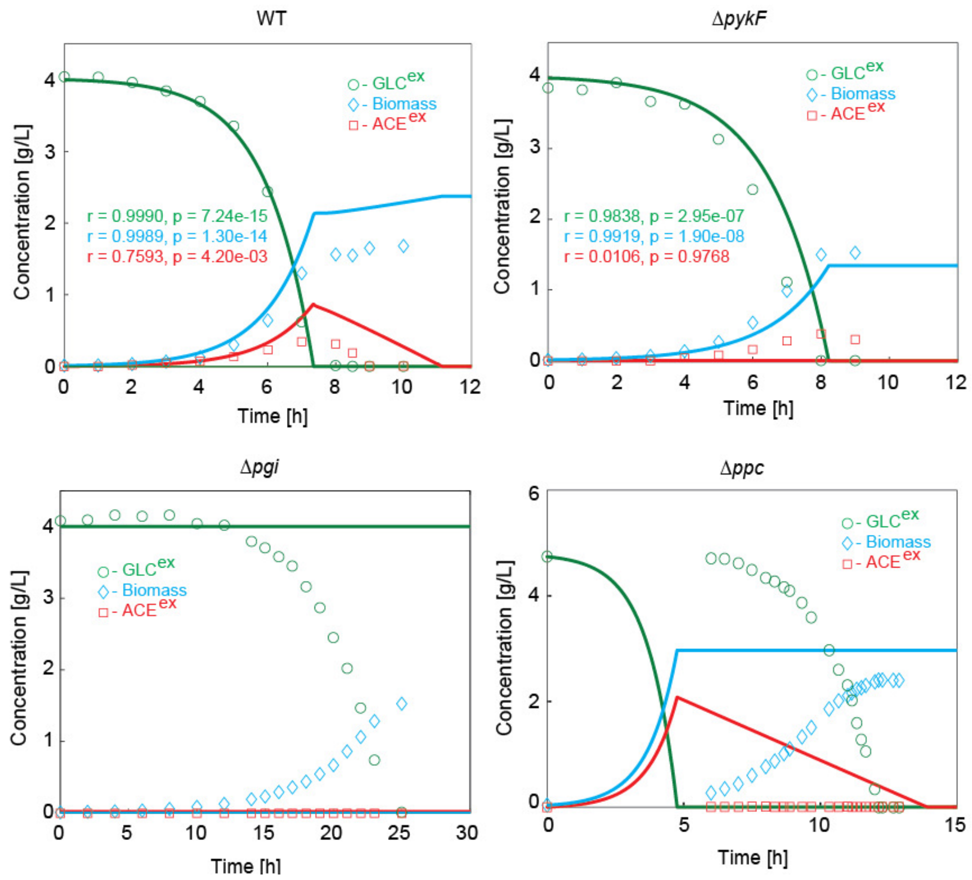

Figure S13: Simulated results by the Matsuoka model [1] for WT, Δ*pykF*, Δ*pgi*, and Δ*ppc* in a batch culture.

The green, blue and red lines represent the simulated results of the extracellular glucose, biomass and acetate, respectively. The corresponding open circles represent the experimental data [2, 3].

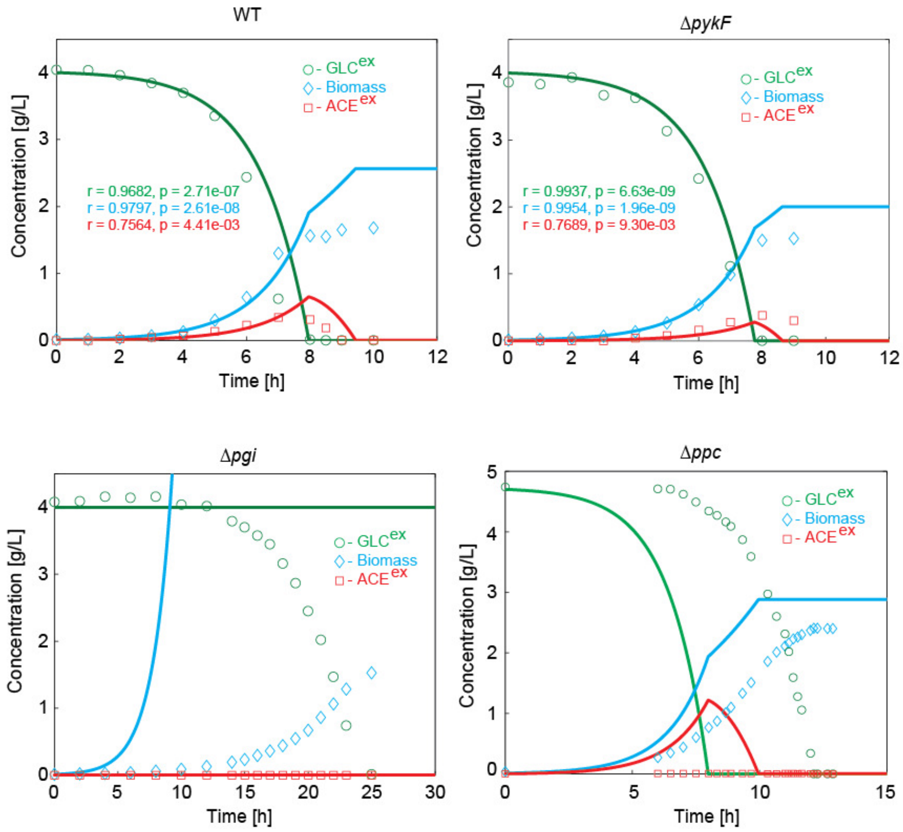

Figure S14: Simulated results by the Kotte model [4] for WT, Δ*pykF*, Δ*pgi*, and Δ*ppc* in a batch culture.

The green, blue and red lines represent the simulated results of the extracellular glucose, biomass and acetate, respectively. The corresponding open circles represent the experimental data [2, 3].

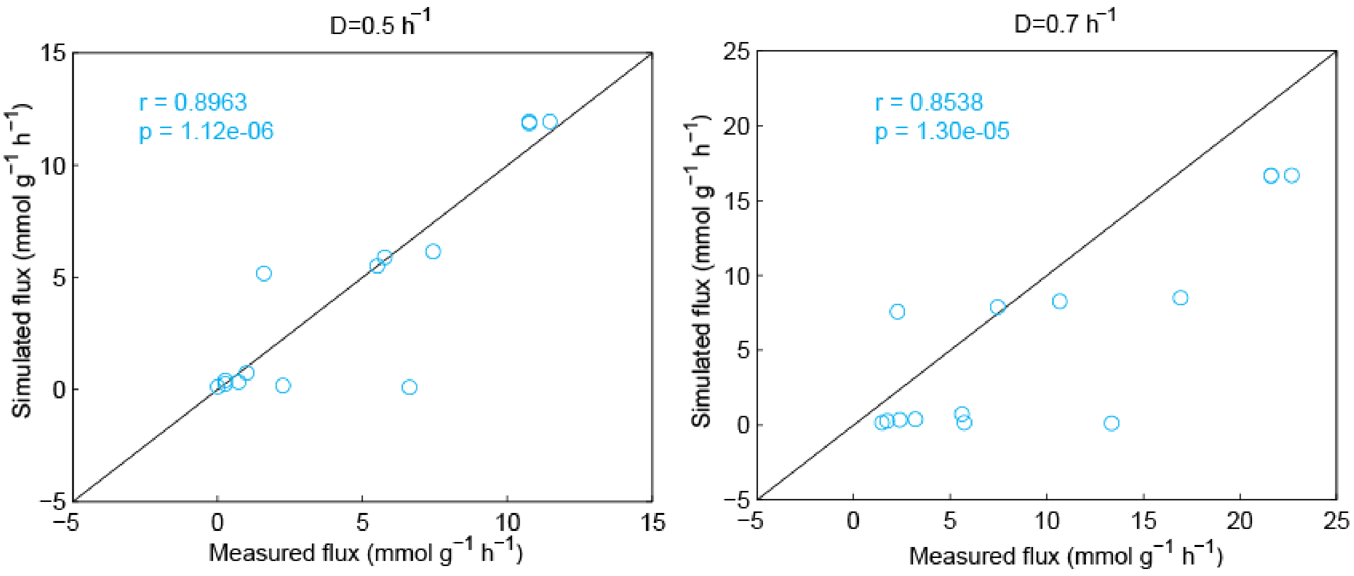

Figure S15: Comparison of the simulated flux by the Chassagnole model [5] with experimental data [6] for WT in a continuous culture.

References

1. Matsuoka Y, Shimizu K: **Catabolite regulation analysis of *Escherichia coli* for acetate overflow mechanism and co-consumption of multiple sugars based on systems biology approach using computer simulation.** *J Biotechnol* 2013, **168:**155-173.
2. Toya Y, Ishii N, Nakahigashi K, Hirasawa T, Soga T, Tomita M, Shimizu K: **13C-metabolic flux analysis for batch culture of *Escherichia coli* and its *Pyk* and *Pgi* gene knockout mutants based on mass isotopomer distribution of intracellular metabolites.** *Biotechnol Prog* 2010, **26:**975-992.
3. Kadir TA, Mannan AA, Kierzek AM, McFadden J, Shimizu K: **Modeling and simulation of the main metabolism in Escherichia coli and its several single-gene knockout mutants with experimental verification.** *Microb Cell Fact* 2010, **9:**88.
4. Kotte O, Zaugg JB, Heinemann M: **Bacterial adaptation through distributed sensing of metabolic fluxes.** *Mol Syst Biol* 2010, **6:**355
5. Chassagnole C, Noisommit-Rizzi N, Schmid JW, Mauch K, Reuss M: **Dynamic modeling of the central carbon metabolism of *Escherichia coli*.** *Biotechnol Bioeng* 2002, **79:**53-73.
6. Ishii N, Nakahigashi K, Baba T, Robert M, Soga T, Kanai A, Hirasawa T, Naba M, Hirai K, Hoque A, et al: **Multiple high-throughput analyses monitor the response of *E. coli* to perturbations.** *Science* 2007, **316:**593-597.
