# Supplementary material for: Development of an accurate kinetic model for the central carbon metabolism of Escherichia coli
Source: Microb Cell Fact. 2016 Jun 21;15:112. doi: 10.1186/s12934-016-0511-x (PMC4915146; doi:10.1186/s12934-016-0511-x)
Supplement: Supplementary file 4 — 10.1186/s12934-016-0511-x Details of the parameter estimation method. [file 12934_2016_511_MOESM3_ESM.pdf]

Details of the kinetic model

Table S1. Differential equations

Differential equation
$\frac{d[X]}{dt}=v_{growth}-v_{D,X}$
$\frac{d[GLC^{ex}]}{dt}=v_{D,GLC^{feed}}-v_{D,GLC^{ex}}-v_{Pts4,medium}-v_{Nonpts,medium}$
$\frac{d[GLC]}{dt}=v_{Nonpts}-v_{E,Glk}-v_{D,GLC}$
$\frac{d[G6P]}{dt}=v_{Pts4}+v_{E,Glk}-v_{E,Pgi}-v_{E,G6pdh}-v_{D,G6P}-v_{BM,G6P}$
$\frac{d[F6P]}{dt}=v_{E,Pgi}+v_{E,TktB}+v_{E,Tal}+v_{E,Fbp}-v_{E,Pfk}-v_{D,F6P}-v_{BM,F6P}$
$\frac{d[FBP]}{dt}=v_{E,Pfk}-v_{E,Fba}-v_{E,Fbp}-v_{D,FBP}$
$\frac{d[GAP]}{dt}=2v_{E,Fba}+v_{E,TktA}+v_{E,TktB}+v_{E,Eda}-v_{E,Tal}-v_{E,Gapdh}-v_{D,GAP}-v_{BM,GAP}$
$\frac{d[PEP]}{dt}=v_{E,Gapdh}+v_{E,Pck}+v_{E,Pps}-v_{E,Pyk}-v_{Pts1}-v_{E,Ppc}-v_{D,PEP}-v_{BM,PEP}$
$\frac{d[PYR]}{dt}=v_{E,Pyk}+v_{Pts1}+v_{E,Mez}+v_{E,Eda}-v_{E,Pdh}-v_{E,Pps}-v_{D,PYR}-v_{BM,PYR}$
$\frac{d[AcCoA]}{dt}=v_{E,Pdh}+v_{E,Acs}-v_{E,Cs}-v_{E,Ms}-v_{E,Pta}-v_{D,AcCoA}-v_{BM,AcCoA}$
$\frac{d[AcP]}{dt}=v_{E,Pta}-v_{E,Ack}-v_{D,AcP}$
$\frac{d[ACE^{ex}]}{dt}=v_{E,Ack,medium}-v_{E,Acs,medium}-v_{D,ACE^{ex}}$
$\frac{d[ICIT]}{dt}=v_{E,Cs}-v_{E,Icdh}-v_{E,Icl}-v_{D,ICIT}$
$\frac{d[\alpha KG]}{dt}=v_{E,Icdh}-v_{E,\alpha kgdh}-v_{D,\alpha KG}-v_{BM,\alpha KG}$
$\frac{d[SUC]}{dt}=v_{E,\alpha kgdh}+v_{E,Icl}-v_{E,Sdh}-v_{D,SUC}-v_{BM,SUC}$
$\frac{d[FUM]}{dt}=v_{E,Sdh}-v_{E,Fum}-v_{D,FUM}-v_{BM,FUM}$
$\frac{d[MAL]}{dt}=v_{E,Fum}+v_{E,Ms}-v_{E,Mdh}-v_{E,Mez}-v_{D,MAL}$
$\frac{d[OAA]}{dt}=v_{E,Mdh}+v_{E,Ppc}-v_{E,Pck}-v_{E,Cs}-v_{D,OAA}-v_{BM,OAA}$
$\frac{d[GOX]}{dt}=v_{E,Icl}-v_{E,Ms}-v_{D,GOX}$
$\frac{d[6PGL]}{dt}=v_{E,G6pdh}-v_{E,Pgl}-v_{D,6PGL}$
$\frac{d[6PG]}{dt}=v_{E,Pgl}-v_{E,6Pgdh}-v_{E,Edd}-v_{D,6PG}$
$\frac{d[KDPG]}{dt}=v_{E,Edd}-v_{E,Eda}-v_{D,KDPG}$
$\frac{d[RU5P]}{dt}=v_{E,6Pgdh}-v_{E,Ru5p}-v_{E,R5pi}-v_{D,RU5P}$
$\frac{d[R5P]}{dt}=v_{E,R5pi}-v_{E,TktA}-v_{D,R5P}-v_{BM,R5P}$
$\frac{d[X5P]}{dt}=v_{E,Ru5p}-v_{E,TktA}-v_{E,TktB}-v_{D,X5P}$
$\frac{d[S7P]}{dt}=v_{E,TktA}-v_{E,Tal}-v_{D,S7P}$
$\frac{d[E4P]}{dt}=v_{E,Tal}-v_{E,TktB}-v_{D,E4P}-v_{BM,E4P}$

$$\frac{d[cAMP]}{dt} = v_{E,Cya} - v_{E,cAMP_{degr}} - v_{D,cAMP}$$
$$\frac{d[EIIA\text{-}P]}{dt} = v_{Pts1} - v_{Pts4}$$
$$\frac{d[Glk]}{dt} = v_{G,glk} - v_{D,Glk}$$
$$\frac{d[Pfk]}{dt} = v_{G,pfkA} - v_{D,Pfk}$$
$$\frac{d[Fbp]}{dt} = v_{G,fbp} - v_{D,Fbp}$$
$$\frac{d[Fba]}{dt} = v_{G,fbaA} - v_{D,Fba}$$
$$\frac{d[Gapdh]}{dt} = v_{G,gapA} - v_{D,Gapdh}$$
$$\frac{d[Pyk]}{dt} = v_{G,pykF} - v_{D,Pyk}$$
$$\frac{d[Pps]}{dt} = v_{G,ppsA} - v_{D,Pps}$$
$$\frac{d[Pdh]}{dt} = v_{G,pdh} - v_{D,Pdh}$$
$$\frac{d[Acs]}{dt} = v_{G,acs} - v_{D,Acs}$$
$$\frac{d[Cs]}{dt} = v_{G,gltA} - v_{D,Cs}$$
$$\frac{d[Icdh]}{dt} = v_{G,icdA} + v_{E,AceK\text{-}ph} - v_{E,AceK\text{-}ki} - v_{D,Icdh}$$
$$\frac{d[Icdh\text{-}P]}{dt} = v_{E,AceK\text{-}ki} - v_{E,AceK\text{-}ph} - v_{D,Icdh\text{-}P}$$
$$\frac{d[\alpha kgdh]}{dt} = v_{G,sucAB} - v_{D,\alpha kgdh}$$
$$\frac{d[Sdh]}{dt} = v_{G,sdhCDAB} - v_{D,Sdh}$$
$$\frac{d[Fum]}{dt} = v_{G,fumABC} - v_{D,Fum}$$
$$\frac{d[Mdh]}{dt} = v_{G,mdh} - v_{D,Mdh}$$
$$\frac{d[Mez]}{dt} = v_{G,maeB} - v_{D,Mez}$$
$$\frac{d[Pck]}{dt} = v_{G,pckA} - v_{D,Pck}$$
$$\frac{d[Ppc]}{dt} = v_{G,ppc} - v_{D,Ppc}$$
$$\frac{d[Icl]}{dt} = v_{G,aceA} - v_{D,Icl}$$
$$\frac{d[Ms]}{dt} = v_{G,aceB} - v_{D,Ms}$$
$$\frac{d[AceK]}{dt} = v_{G,aceK} - v_{D,AceK}$$

Table S2. Rate equations

Rate equation	Reference
$v_{growth} = \mu[X]$	-
$v_{Pts1} = k_{Pts1}[PEP][EIIA] - km_{Pts1}[PYR][EIIA\text{-}P]$	[1]
$v_{Pts4} = \frac{v_{Pts4}^{\max}[EIIA\text{-}P][GLC^{ex}]}{\left(K_{Pts\_EIIA} + [EIIA\text{-}P]\right)\left(K_{Pts\_GLC} + [GLC^{ex}]\right)}$	[1]
$v_{Pts4,medium} = v_{Pts4}\frac{[X]}{\rho}$	-
$v_{Nonpts} = \frac{v_{Nonpts}^{\max}[GLC^{ex}]}{K_{Nonpts\_S} + \left(1 + \frac{[EIIA]}{K_{Nonpts\_I}}\right)[GLC^{ex}]}$	[2]
$v_{Nonpts,medium} = v_{Nonpts}\frac{[X]}{\rho}$	-
$v_{E,Glk} = \frac{[Glk]k_{Glk\_cat}\left(\frac{[GLC]}{K_{Glk\_GLC\_m}}\right)\left(\frac{[ATP]}{K_{Glk\_ATP\_m}\left(1+\frac{[G6P]}{K_{Glk\_G6P\_i}}\right)}\right)}{1+\frac{[GLC]}{K_{Glk\_GLC\_m}} + \frac{[ATP]}{K_{Glk\_ATP\_m}\left(1+\frac{[G6P]}{K_{Glk\_G6P\_i}}\right)} + \frac{[GLC][ATP]}{K_{Glk\_GLC\_m}K_{Glk\_ATP\_m}\left(1+\frac{[G6P]}{K_{Glk\_G6P\_i}}\right)} + \frac{[G6P]}{K_{Glk\_G6P\_i}}}$	[3]
$v_{E,Pgi} = \frac{v_{Pgi}^{\max}\left([G6P] - \frac{[F6P]}{K_{Pgi\_eq}}\right)}{K_{Pgi\_G6P}\left(1 + \frac{[F6P]}{K_{Pgi\_F6P}\left(1+\frac{[6PG]}{K_{Pgi\_F6P\_6pginh}}\right)} + \frac{[6PG]}{K_{Pgi\_G6P\_6pginh}}\right) + [G6P]}$	[4]
$v_{E,Pfk} = \frac{[Pfk]k_{Pfk\_cat}[ATP][F6P]}{\left([ATP] + K_{Pfk\_ATP\_s}\left(1+\frac{[ADP]}{K_{Pfk\_ADP\_c}}\right)\right)\left([F6P] + K_{Pfk\_F6P\_s}\cdot\frac{A}{B}\right)\left(1 + \frac{L_{Pfk}}{\left(1+[F6P]\frac{B}{K_{Pfk\_F6P\_s}A}\right)^{n_{Pfk}}}\right)}$ $A = 1 + \frac{[PEP]}{K_{Pfk\_PEP}} + \frac{[ADP]}{K_{Pfk\_ADP\_b}} + \frac{[AMP]}{K_{Pfk\_AMP\_b}}$ $B = 1 + \frac{[ADP]}{K_{Pfk\_ADP\_a}} + \frac{[AMP]}{K_{Pfk\_AMP\_a}}$	[4]
$v_{E,Fbp} = \frac{[Fbp]k_{Fbp\_cat}\frac{[FBP]}{K_{Fbp\_FBP}}\left(1+\frac{[FBP]}{K_{Fbp\_FBP}}\right)^{n_{Fbp}-1}}{\left(1+\frac{[FBP]}{K_{Fbp\_FBP}}\right)^{n_{Fbp}} + \frac{L_{Fbp}}{\left(1+\frac{[PEP]}{K_{Fbp\_PEP}}\right)^{n_{Fbp}}}}$	[1]
$v_{E,Fba} = \frac{[Fba]k_{Fba\_cat}\left([FBP] - \frac{[GAP]^2}{K_{Fba\_eq}}\right)}{K_{Fba\_FBP} + [FBP] + \frac{K_{Fba\_GAP}[GAP]}{K_{Fba\_eq}V_{Fba\_blf}} + \frac{K_{Fba\_DHAP}[GAP]}{K_{Fba\_eq}V_{Fba\_blf}} + \frac{[FBP][GAP]}{K_{Fba\_GAP\_inh}} + \frac{[GAP]^2}{K_{Fba\_eq}V_{Fba\_blf}}}$	[4]

$v_{E,Gapdh} = \frac{[Gapdh]k_{Gapdh\_cat}\left([GAP][NAD]-\frac{[PEP][NADH]}{K_{Gapdh\_eq}}\right)}{\left(K_{Gapdh\_GAP}\left(1+\frac{[PEP]}{K_{Gapdh\_PGP}}\right)+[GAP]\right)\left(K_{Gapdh\_NAD}\left(1+\frac{[NADH]}{K_{Gapdh\_NADH}}\right)+[NAD]\right)}$	[4]
$v_{E,Pyk} = \frac{[Pyk]k_{Pyk\_cat}[PEP]\left(\frac{[PEP]}{K_{Pyk\_PEP}}+1\right)^{n_{Pyk}-1}[ADP]}{K_{Pyk\_PEP}\left(L_{Pyk}\left(\frac{1+\frac{[ATP]}{K_{Pyk\_ATP}}}{\frac{[FBP]}{K_{Pyk\_FBP}}+\frac{[AMP]}{K_{Pyk\_AMP}}+1}\right)^{n_{Pyk}}+\left(\frac{[PEP]}{K_{Pyk\_PEP}}+1\right)^{n_{Pyk}}\right)\left([ADP]+K_{Pyk\_ADP}\right)}$	[4]
$v_{E,Pps} = \frac{[Pps]k_{Pps\_cat}\frac{[PYR]}{K_{Pps\_PYR}}\left(1+\frac{[PYR]}{K_{Pps\_PYR}}\right)^{n_{Pps}-1}}{\left(1+\frac{[PYR]}{K_{Pps\_PYR}}\right)^{n_{Pps}}+L_{Pps}\left(1+\frac{[PEP]}{K_{Pps\_PEP}}\right)^{n_{Pps}}}$	[1]
$v_{E,Pdh} = \frac{[Pdh]k_{Pdh\_cat}\left(\frac{1}{1+K_{Pdh\_i}\frac{[NADH]}{[NAD]}}\right)\left(\frac{[PYR]}{K_{Pdh\_PYR\_m}}\right)\left(\frac{[NAD]}{K_{Pdh\_NAD\_m}}\right)\left(\frac{[CoA]}{K_{Pdh\_CoA\_m}}\right)}{\left(1+\frac{[PYR]}{K_{Pdh\_PYR\_m}}\right)\left(1+\frac{[NAD]}{K_{Pdh\_NAD\_m}}+\frac{[NADH]}{K_{Pdh\_NADH\_m}}\right)\left(1+\frac{[CoA]}{K_{Pdh\_CoA\_m}}+\frac{[AcCoA]}{K_{Pdh\_AcCoA\_m}}\right)}$	[5]
$v_{E,Pta} = \frac{v_{Pta}^{\max}\left(\frac{1}{K_{Pta\_AcCoA\_i}K_{Pta\_Pi\_m}}\right)\left([AcCoA][P_i]-\frac{[AcP][CoA]}{K_{Pta\_eq}}\right)}{1+\frac{[AcCoA]}{K_{Pta\_AcCoA\_i}}+\frac{[P_i]}{K_{Pta\_Pi\_i}}+\frac{[AcP]}{K_{Pta\_AcP\_i}}+\frac{[CoA]}{K_{Pta\_CoA\_i}}+\left(\frac{[AcCoA][P_i]}{K_{Pta\_AcCoA\_i}K_{Pta\_Pi\_m}}\right)+\left(\frac{[AcP][CoA]}{K_{Pta\_AcP\_m}K_{Pta\_CoA\_i}}\right)}$	[5]
$v_{E,Ack} = \frac{v_{Ack}^{\max}\left(\frac{1}{K_{Ack\_ADP\_m}K_{Ack\_AcP\_m}}\right)\left([AcP][ADP]-\frac{[ACE^{ex}][ATP]}{K_{Ack\_eq}}\right)}{\left(1+\frac{[AcP]}{K_{Ack\_AcP\_m}}+\frac{[ACE^{ex}]}{K_{Ack\_ACE\_m}}\right)\left(1+\frac{[ADP]}{K_{Ack\_ADP\_m}}+\frac{[ATP]}{K_{Ack\_ATP\_m}}\right)}$	[5]
$v_{E,Ack,medium} = v_{E,Ack}\frac{[X]}{\rho}$	-
$v_{E,Acs} = \frac{[Acs]k_{Acs\_cat}[ACE^{ex}]}{[ACE^{ex}]+K_{Acs\_ACE}}$	[1]
$v_{E,Acs,medium} = v_{E,Acs}\frac{[X]}{\rho}$	-
$v_{E,Cs} = \frac{[Cs]k_{Cs\_cat}[OAA][AcCoA]}{\left(1+\frac{[\alpha KG]}{K_{Cs\_\alpha KG}}\right)K_{Cs\_OAA\_AcCoA}K_{Cs\_AcCoA}+K_{Cs\_AcCoA}[OAA]+\left(1+\frac{[\alpha KG]}{K_{Cs\_\alpha KG}}\right)K_{Cs\_OAA}[AcCoA]+[OAA][AcCoA]}$	[1]
$v_{E,Icdh} = \frac{[Icdh]k_{Icdh\_cat}\frac{[ICIT]}{K_{Icdh\_ICIT}}\left(1+\frac{[ICIT]}{K_{Icdh\_ICIT}}\right)^{n_{Icdh}-1}}{\left(1+\frac{[ICIT]}{K_{Icdh\_ICIT}}\right)^{n_{Icdh}}+L_{Icdh}\left(1+\frac{[PEP]}{K_{Icdh\_PEP}}\right)^{n_{Icdh}}}$	[1]

[6]

$$v_{E,\alpha kgdh}=\frac{[\alpha kgdh]k_{\alpha kgdh\_cat}[\alpha KG][CoA][NAD]}{\left(\begin{array}{l}K_{\alpha kgdh\_NAD\_m}[\alpha KG][CoA]+K_{\alpha kgdh\_CoA\_m}[\alpha KG][NAD]+K_{\alpha kgdh\_\alpha KG\_m}[CoA][NAD]\\+[\alpha KG][CoA][NAD]+\frac{K_{\alpha kgdh\_\alpha KG\_m}K_{\alpha kgdh\_Z}[SUC][NADH]}{K_{\alpha kgdh\_SUC\_I}}+\frac{K_{\alpha kgdh\_NAD\_m}[\alpha KG][CoA][NADH]}{K_{\alpha kgdh\_NADH\_I}}\\+\frac{K_{\alpha kgdh\_CoA\_m}[\alpha KG][NAD][SUC]}{K_{\alpha kgdh\_SUC\_I}}+\frac{K_{\alpha kgdh\_\alpha KG\_m}K_{\alpha kgdh\_Z}[\alpha KG][SUC][NADH]}{K_{\alpha kgdh\_\alpha KG\_I}K_{\alpha kgdh\_SUC\_I}}\end{array}\right)}$$

---

[6]

$$v_{Sdh1}^{\max}=[Sdh]k_{Sdh1\_cat}$$

$$v_{Sdh2}^{\max}=[Sdh]k_{Sdh2\_cat}$$

$$v_{E,Sdh}=\frac{v_{Sdh1}^{\max}v_{Sdh2}^{\max}\left([SUC]-\frac{[FUM]}{K_{Sdh\_eq}}\right)}{K_{Sdh\_SUC\_m}v_{Sdh2}^{\max}+v_{Sdh2}^{\max}[SUC]+\frac{v_{Sdh1}^{\max}[FUM]}{K_{Sdh\_eq}}}$$

---

[6]

$$v_{Fum1}^{\max}=[Fum]k_{Fum1\_cat}$$

$$v_{Fum2}^{\max}=[Fum]k_{Fum2\_cat}$$

$$v_{E,Fum}=\frac{v_{Fum1}^{\max}v_{Fum2}^{\max}\left([FUM]-\frac{[MAL]}{K_{Fum\_eq}}\right)}{K_{Fum\_FUM\_m}v_{Fum2}^{\max}+v_{Fum2}^{\max}[FUM]+\frac{v_{Fum1}^{\max}[MAL]}{K_{Fum\_eq}}}$$

---

[6]

$$v_{Mdh1}^{\max}=[Mdh]k_{Mdh1\_cat}$$

$$v_{Mdh2}^{\max}=[Mdh]k_{Mdh2\_cat}$$

$$v_{E,Mdh}=\frac{v_{Mdh1}^{\max}v_{Mdh2}^{\max}\left([NAD][MAL]-\frac{[NADH][OAA]}{K_{Mdh\_eq}}\right)}{\left(\begin{array}{l}K_{Mdh\_NAD\_I}K_{Mdh\_MAL\_m}v_{Mdh2}^{\max}+K_{Mdh\_MAL\_m}v_{Mdh2}^{\max}[NAD]+K_{Mdh\_NAD\_m}v_{Mdh2}^{\max}[MAL]\\+v_{Mdh2}^{\max}[NAD][MAL]+\frac{K_{Mdh\_OAA\_m}v_{Mdh1}^{\max}[NADH]}{K_{Mdh\_eq}}+\frac{K_{Mdh\_NADH\_m}v_{Mdh1}^{\max}[OAA]}{K_{Mdh\_eq}}\\+\frac{v_{Mdh1}^{\max}[NADH][OAA]}{K_{Mdh\_eq}}+\frac{v_{Mdh1}^{\max}K_{Mdh\_OAA\_m}[NAD][NADH]}{K_{Mdh\_eq}K_{Mdh\_NAD\_I}}+\frac{v_{Mdh2}^{\max}K_{Mdh\_NAD\_m}[MAL][OAA]}{K_{Mdh\_OAA\_I}}\\+\frac{v_{Mdh2}^{\max}[NAD][MAL][NADH]}{K_{Mdh\_NADH\_I}}+\frac{v_{Mdh1}^{\max}[MAL][NADH][OAA]}{K_{Mdh\_eq}K_{Mdh\_MAL\_I}}+\frac{v_{Mdh2}^{\max}[NAD][MAL][OAA]}{K_{Mdh\_OAA\_II}}\\+\frac{v_{Mdh1}^{\max}[NAD][NADH][OAA]}{K_{Mdh\_NAD\_II}K_{Mdh\_eq}}+\frac{K_{Mdh\_NAD\_I}v_{Mdh2}^{\max}[NAD][MAL][NADH][OAA]}{K_{Mdh\_NAD\_II}K_{Mdh\_OAA\_m}K_{Mdh\_NADH\_I}}\end{array}\right)}$$

---

[1]

$$v_{E,Mez}=\frac{[Mez]k_{Mez\_cat}\frac{[MAL]}{K_{Mez\_MAL}}\left(1+\frac{[MAL]}{K_{Mez\_MAL}}\right)^{n_{Mez}-1}}{\left(1+\frac{[MAL]}{K_{Mez\_MAL}}\right)^{n_{Mez}}+L_{Mez}\left(1+\frac{[AcCoA]}{K_{Mez\_AcCoA}}+\frac{[cAMP]}{K_{Mez\_cAMP}}\right)^{n_{Mez}}}$$

---

[7]

$$v_{E,Pck}=\frac{[Pck]k_{Pck\_cat}[OAA]\frac{[ATP]}{[ADP]}}{\left(\begin{array}{l}K_{Pck\_OAA}\cdot\frac{[ATP]}{[ADP]}+[OAA]\cdot\frac{[ATP]}{[ADP]}+\frac{K_{Pck\_ATP\_i}K_{Pck\_OAA}}{K_{Pck\_ADP\_i}}+\frac{K_{Pck\_ATP\_i}K_{Pck\_OAA}}{K_{Pck\_PEP}K_{Pck\_ADP\_i}}\cdot[PEP]\\+\frac{K_{Pck\_ATP\_i}K_{Pck\_OAA}}{K_{Pck\_PEP\_i}K_{Pck\_ATP\_I}}\cdot\frac{[ATP]}{[ADP]}\cdot[PEP]+\frac{K_{Pck\_ATP\_i}K_{Pck\_OAA}}{K_{Pck\_ADP\_i}K_{Pck\_OAA\_I}}\cdot[OAA]\end{array}\right)}$$

$v_{E,Ppc} = \dfrac{[Ppc]k_{Ppc\_cat}\dfrac{[PEP]}{K_{Ppc\_PEP}}\left(1+\dfrac{[PEP]}{K_{Ppc\_PEP}}\right)^{n_{Ppc}-1}}{\left(1+\dfrac{[PEP]}{K_{Ppc\_PEP}}\right)^{n_{Ppc}}+\dfrac{L_{Ppc}}{\left(1+\dfrac{[FBP]}{K_{Ppc\_FBP}}\right)^{n_{Ppc}}}}$	[1]
$v_{E,Icl} = \dfrac{[Icl]k_{Icl\_cat}\dfrac{[ICIT]}{K_{Icl\_ICIT}}\left(1+\dfrac{[ICIT]}{K_{Icl\_ICIT}}\right)^{n_{Icl}-1}}{\left(1+\dfrac{[ICIT]}{K_{Icl\_ICIT}}\right)^{n_{Icl}}+L_{Icl}\left(1+\dfrac{[PEP]}{K_{Icl\_PEP}}+\dfrac{[GAP]}{K_{Icl\_3PG}}+\dfrac{[\alpha KG]}{K_{Icl\_\alpha KG}}\right)^{n_{Icl}}}$	[1]
$v_{E,Ms} = \dfrac{[Ms]k_{Ms\_cat}[GOX][AcCoA]}{K_{Ms\_GOX\_AcCoA}K_{Ms\_AcCoA}+K_{Ms\_AcCoA}[GOX]+K_{Ms\_GOX}[AcCoA]+[GOX][AcCoA]}$	[1]
$v_{E,AceK\text{-}ki} = \dfrac{[AceK]\,k_{AceK\text{-}ki\_cat}\dfrac{[ICDH]}{K_{AceK\_ICDH}}\left(1+\dfrac{[ICDH]}{K_{AceK\_ICDH}}\right)^{n_{AceK}-1}}{\left(1+\dfrac{[ICDH]}{K_{AceK\_ICDH}}\right)^{n_{AceK}}+L_{AceK}\left(\begin{array}{l}1+\dfrac{[ICIT]}{K_{AceK\_ICIT}}+\dfrac{[GOX]}{K_{AceK\_GOX}}+\dfrac{[OAA]}{K_{AceK\_OAA}}\\+\dfrac{[\alpha KG]}{K_{AceK\_\alpha KG}}+\dfrac{[PEP]}{K_{AceK\_PEP}}+\dfrac{[GAP]}{K_{AceK\_3PG}}+\dfrac{[PYR]}{K_{AceK\_PYR}}\end{array}\right)^{n_{AceK}}}$	[1]
$v_{E,AceK\text{-}ph} = \dfrac{[AceK]\,k_{AceK\text{-}ph\_cat}\dfrac{[ICDH\text{-}P]}{K_{AceK\_ICDH\text{-}P}}\left(1+\dfrac{[ICDH\text{-}P]}{K_{AceK\_ICDH\text{-}P}}\right)^{n_{AceK}-1}}{\left(1+\dfrac{[ICDH\text{-}P]}{K_{AceK\_ICDH\text{-}P}}\right)^{n_{AceK}}+\dfrac{L_{AceK}}{\left(\begin{array}{l}1+\dfrac{[OAA]}{K_{AceK\_OAA}}+\dfrac{[\alpha KG]}{K_{AceK\_\alpha KG}}+\dfrac{[PEP]}{K_{AceK\_PEP}}\\+\dfrac{[GAP]}{K_{AceK\_3PG}}+\dfrac{[PYR]}{K_{AceK\_PYR}}\end{array}\right)^{n_{AceK}}}}$	[1]
$v_{E,G6pdh} = \dfrac{v_{G6pdh}^{\max}[G6P][NADP]}{\left([G6P]+K_{G6pdh\_G6P}\right)\left(1+\dfrac{[NADPH]}{K_{G6pdh\_NADPH\_g6pinh}}\right)\left(K_{G6pdh\_NADP}\left(1+\dfrac{[NADPH]}{K_{G6pdh\_NADPH\_nadpinh}}\right)+[NADP]\right)}$	[4]
$v_{E,Pgl} = \dfrac{v_{Pgl}^{\max}\left([6PGL]-\dfrac{[6PG]}{K_{Pgl\_eq}}\right)}{\left(1+\dfrac{[H^+]}{K_{Pgl\_h1}}+\dfrac{K_{Pgl\_h2}}{[H^+]}\right)\left(K_{Pgl\_6PGL\_m}+[6PGL]+\dfrac{K_{Pgl\_6PGL\_m}}{K_{Pgl\_6PG\_m}}[6PG]\right)}$	[8]
$v_{E,Edd} = \dfrac{v_{Edd}^{\max}Q_{Edd\_pH}\left([6PG]-\dfrac{[KDPG]}{K_{Edd\_eq}}\right)}{K_{Edd\_6PG\_m}+[6PG]+\dfrac{K_{Edd\_6PG\_m}[KDPG]}{K_{Edd\_KDPG\_m}}}$ $Q_{Edd\_pH} = \dfrac{1+2\cdot 10^{\left(pH_{Edd\_m}-pK_{Edd}\right)}}{1+10^{\left(pH-pK_{Edd}\right)}+10^{\left(2\cdot pH_{Edd\_m}-pH-pK_{Edd}\right)}}$	[8]
$v_{E,Eda} = \dfrac{v_{Eda}^{\max}Q_{Eda\_pH}\left([KDPG]-\dfrac{[GAP][PYR]}{K_{Eda\_eq}}\right)}{K_{Eda\_KDPG\_m}+[KDPG]+K_{Eda\_KDPG\_m}\left(\dfrac{[PYR]}{K_{Eda\_PYR\_m}}+\dfrac{[GAP]}{K_{Eda\_GAP\_m}}+\dfrac{[PYR][GAP]}{K_{Eda\_PYR\_m}K_{Eda\_GAP\_m}}\right)}$ $Q_{Eda\_pH} = \dfrac{1+2\cdot 10^{\left(pH_{Eda\_m}-pK_{Eda}\right)}}{1+10^{\left(pH-pK_{Eda}\right)}+10^{\left(2\cdot pH_{Eda\_m}-pH-pK_{Eda}\right)}}$	[8]
$v_{E,6Pgdh} = \dfrac{v_{6Pgdh}^{\max}[6PG][NADP]}{\left([6PG]+K_{6Pgdh\_6PG}\right)\left([NADP]+K_{6Pgdh\_NADP}\left(1+\dfrac{[NADPH]}{K_{6Pgdh\_NADPH\_inh}}\right)\left(1+\dfrac{[ATP]}{K_{6Pgdh\_ATP\_inh}}\right)\right)}$	[4]

$v_{E,R5pi} = v_{R5pi}^{\max}\left([RU5P] - \frac{[R5P]}{K_{R5pi\_eq}}\right)$	[4]
$v_{E,Ru5p} = v_{Ru5p}^{\max}\left([RU5P] - \frac{[X5P]}{K_{Ru5p\_eq}}\right)$	[4]
$v_{E,TktA} = v_{TktA}^{\max}\left([R5P][X5P] - \frac{[S7P][GAP]}{K_{TktA\_eq}}\right)$	[4]
$v_{E,TktB} = v_{TktB}^{\max}\left([X5P][E4P] - \frac{[F6P][GAP]}{K_{TktB\_eq}}\right)$	[4]
$v_{E,Tal} = v_{Tal}^{\max}\left([GAP][S7P] - \frac{[E4P][F6P]}{K_{Tal\_eq}}\right)$	[4]
$v_{E,Cya} = \frac{v_{Cya}^{\max}[EIIA\text{-}P]}{[EIIA\text{-}P] + K_{Cya\_EIIA\text{-}P}}$	[1]
$v_{E,cAMP_{degr}} = \frac{v_{cAMP_{degr}}^{\max}[cAMP]}{[cAMP] + K_{cAMP_{degr}\_cAMP}}$	[1]
$v_{G,glk} = \mu k_{expr}\left(\left(1 - \frac{[Cra]}{[Cra]+K_{glk\_Cra}}\right)v_{glk\_Cra\_unbound} + \frac{[Cra]}{[Cra]+K_{glk\_Cra}}v_{glk\_Cra\_bound}\right)$	[1]
$v_{G,pfkA} = \mu k_{expr}\left(\left(1 - \frac{[Cra]}{[Cra]+K_{pfkA\_Cra}}\right)v_{pfkA\_Cra\_unbound} + \frac{[Cra]}{[Cra]+K_{pfkA\_Cra}}v_{pfkA\_Cra\_bound}\right)$	[1]
$v_{G,fbp} = \mu k_{expr}\left(\left(1 - \frac{[Cra]}{[Cra]+K_{fbp\_Cra}}\right)v_{fbp\_Cra\_unbound} + \frac{[Cra]}{[Cra]+K_{fbp\_Cra}}v_{fbp\_Cra\_bound}\right)$	[1]
$v_{G,fbaA} = \mu k_{expr}\left(\begin{array}{l}\left(1 - \frac{[Cra]}{[Cra]+K_{fbaA\_Cra}}\right)v_{fbaA\_Cra\_unbound} + \frac{[Cra]}{[Cra]+K_{fbaA\_Cra}}v_{fbaA\_Cra\_bound} \\ +\left(1 - \frac{[Crp\text{-}cAMP]}{[Crp\text{-}cAMP]+K_{fbaA\_Crp}}\right)v_{fbaA\_Crp\_unbound} + \frac{[Crp\text{-}cAMP]}{[Crp\text{-}cAMP]+K_{fbaA\_Crp}}v_{fbaA\_Crp\_bound}\end{array}\right)$	[1]
$v_{G,gapA} = \mu k_{expr}\left(\begin{array}{l}\left(1 - \frac{[Cra]}{[Cra]+K_{gapA\_Cra}}\right)v_{gapA\_Cra\_unbound} + \frac{[Cra]}{[Cra]+K_{gapA\_Cra}}v_{gapA\_Cra\_bound} \\ +\left(1 - \frac{[Crp\text{-}cAMP]}{[Crp\text{-}cAMP]+K_{gapA\_Crp}}\right)v_{gapA\_Crp\_unbound} + \frac{[Crp\text{-}cAMP]}{[Crp\text{-}cAMP]+K_{gapA\_Crp}}v_{gapA\_Crp\_bound}\end{array}\right)$	[1]
$v_{G,pykF} = \mu k_{expr}\left(\left(1 - \frac{[Cra]}{[Cra]+K_{pykF\_Cra}}\right)v_{pykF\_Cra\_unbound} + \frac{[Cra]}{[Cra]+K_{pykF\_Cra}}v_{pykF\_Cra\_bound}\right)$	[1]
$v_{G,ppsA} = \mu k_{expr}\left(\left(1 - \frac{[Cra]}{[Cra]+K_{ppsA\_Cra}}\right)v_{ppsA\_Cra\_unbound} + \frac{[Cra]}{[Cra]+K_{ppsA\_Cra}}v_{ppsA\_Cra\_bound}\right)$	[1]
$v_{G,pdh} = \mu k_{expr}\left(\left(1 - \frac{[PdhR]}{[PdhR]+K_{pdh\_PdhR}}\right)v_{pdh\_PdhR\_unbound} + \frac{[PdhR]}{[PdhR]+K_{pdh\_PdhR}}v_{pdh\_PdhR\_bound}\right)$	[1]
$v_{G,acs} = \mu k_{expr}\left(\left(1 - \frac{[Crp\text{-}cAMP]^{n_{acs}}}{[Crp\text{-}cAMP]^{n_{acs}}+K_{acs\_Crp}^{\ n_{acs}}}\right)v_{acs\_Crp\_unbound} + \frac{[Crp\text{-}cAMP]^{n_{acs}}}{[Crp\text{-}cAMP]^{n_{acs}}+K_{acs\_Crp}^{\ n_{acs}}}v_{acs\_Crp\_bound}\right)$	[1]
$v_{G,gltA} = \mu k_{expr}\left(\left(1 - \frac{[Crp\text{-}cAMP]^{n_{gltA}}}{[Crp\text{-}cAMP]^{n_{gltA}}+K_{gltA\_Crp}^{\ n_{gltA}}}\right)v_{gltA\_Crp\_unbound} + \frac{[Crp\text{-}cAMP]^{n_{gltA}}}{[Crp\text{-}cAMP]^{n_{gltA}}+K_{gltA\_Crp}^{\ n_{gltA}}}v_{gltA\_Crp\_bound}\right)$	[1]
$v_{G,icdA} = \mu k_{expr}\left(\left(1 - \frac{[Cra]}{[Cra]+K_{icdA\_Cra}}\right)v_{icdA\_Cra\_unbound} + \frac{[Cra]}{[Cra]+K_{icdA\_Cra}}v_{icdA\_Cra\_bound}\right)$	[1]
$v_{G,sucAB} = \mu k_{expr}\left(\begin{array}{l}\left(1 - \frac{[Crp\text{-}cAMP]^{n_{sucAB}}}{[Crp\text{-}cAMP]^{n_{sucAB}}+K_{sucAB\_Crp}^{\ n_{sucAB}}}\right)v_{sucAB\_Crp\_unbound} \\ +\frac{[Crp\text{-}cAMP]^{n_{sucAB}}}{[Crp\text{-}cAMP]^{n_{sucAB}}+K_{sucAB\_Crp}^{\ n_{sucAB}}}v_{sucAB\_Crp\_bound}\end{array}\right)$	[1]

$v_{G,sdhCDAB} = \mu k_{expr} \left(\left(1 - \frac{[Crp\text{-}cAMP]^{n_{sdhCDAB}}}{[Crp\text{-}cAMP]^{n_{sdhCDAB}} + K_{sdhCDAB\_Crp}{}^{n_{sdhCDAB}}} \right) v_{sdhCDAB\_Crp\_unbound} + \frac{[Crp\text{-}cAMP]^{n_{sdhCDAB}}}{[Crp\text{-}cAMP]^{n_{sdhCDAB}} + K_{sdhCDAB\_Crp}{}^{n_{sdhCDAB}}} v_{sdhCDAB\_Crp\_bound} \right)$	[1]
$v_{G,fumABC} = \mu k_{expr} \left(\left(1 - \frac{[Crp\text{-}cAMP]^{n_{fumABC}}}{[Crp\text{-}cAMP]^{n_{fumABC}} + K_{fumABC\_Crp}{}^{n_{fumABC}}} \right) v_{fumABC\_Crp\_unbound} + \frac{[Crp\text{-}cAMP]^{n_{fumABC}}}{[Crp\text{-}cAMP]^{n_{fumABC}} + K_{fumABC\_Crp}{}^{n_{fumABC}}} v_{fumABC\_Crp\_bound} \right)$	[1]
$v_{G,mdh} = \mu k_{expr} \left(\left(1 - \frac{[Crp\text{-}cAMP]}{[Crp\text{-}cAMP] + K_{mdh\_Crp}} \right) v_{mdh\_Crp\_unbound} + \frac{[Crp\text{-}cAMP]}{[Crp\text{-}cAMP] + K_{mdh\_Crp}} v_{mdh\_Crp\_bound} \right)$	[1]
$SS_{Mez} = \alpha_{GLC} SS_{Mez}^{GLC} + \alpha_{ACE} SS_{Mez}^{ACE}$<br>$v_{G,maeB} = \left(\mu + k_{degr} \right) SS_{Mez}$	[1]
$v_{G,pckA} = \mu k_{expr} \left(\left(1 - \frac{[Cra]}{[Cra] + K_{pckA\_Cra}} \right) v_{pckA\_Cra\_unbound} + \frac{[Cra]}{[Cra] + K_{pckA\_Cra}} v_{pckA\_Cra\_bound} \right)$	[1]
$SS_{Ppc} = \alpha_{GLC} SS_{Ppc}^{GLC} + \alpha_{ACE} SS_{Ppc}^{ACE}$<br>$v_{G,ppc} = \left(\mu + k_{degr} \right) SS_{Ppc}$	[1]
$v_{G,aceA} = \mu k_{\exp r} \left(\left(1 - \frac{[Cra]}{[Cra\ ] + K_{aceBAK\_Cra}} \right) v_{aceBAK\_Cra\_unbound} + \frac{[Cra]}{[Cra] + K_{aceBAK\_Cra}} v_{aceBAK\_Cra\_bound} + \left(1 - \frac{[Crp\text{ - }cAMP]}{[Crp\text{ - }cAMP] + K_{aceBAK\_Crp}} \right) v_{aceBAK\_Crp\_unbound} + \frac{[Crp\text{ - }cAMP]}{[Crp\text{ - }cAMP\ ] + K_{aceBAK\_Crp}} v_{aceBAK\_Crp\_bound} + \left[1 - \frac{[aceBAK\_DNA]}{K_{aceBAK\_DNA}} \frac{\left(1 + \frac{[PYR]}{K_{aceBAK\_PYR_{prime}}} \right)}{\left(1 + \frac{1}{L_{aceBAK}} \left(\frac{[GOX]}{K_{aceBAK\_GOX}} \right) \left(1 + \frac{[GOX]}{K_{aceBAK\_GOX}} \right) + \frac{[aceBAK\_DNA]}{K_{aceBAK\_DNA}} + \frac{[PYR]}{K_{aceBAK\_PYR}} + \frac{[aceBAK\_DNA]}{K_{aceBAK\_DNA}} \frac{[PYR]}{K_{aceBAK\_PYR_{prime}}} \right)} \right] .k_{aceBAK\_cat\_IclR} [IclR_{total}] \right)$	[1]
$v_{G,aceB} = Factor_{aceB} \cdot v_{G,aceA}$	[1]
$v_{G,aceK} = Factor_{aceK} \cdot v_{G,aceA}$	[1]
$v_{D,X} = D[X]$	-
$v_{D,GLC^{feed}} = D\left[GLC^{feed} \right]$	-
$v_{D,GLC^{ex}} = D\left[GLC^{ex} \right]$	-
$v_{D,GLC} = \mu [GLC]$	-
$v_{D,G6P} = \mu [G6P]$	-
$v_{D,F6P} = \mu [F6P]$	-
$v_{D,FBP} = \mu [FBP]$	-

$v_{D,GAP} = \mu[GAP]$	-
$v_{D,PEP} = \mu[PEP]$	-
$v_{D,PYR} = \mu[PYR]$	-
$v_{D,AcCoA} = \mu[AcCoA]$	-
$v_{D,AcP} = \mu[AcP]$	-
$v_{D,ACE^{ex}} = D\left[ACE^{ex}\right]$	-
$v_{D,ICIT} = \mu[ICIT]$	-
$v_{D,\alpha KG} = \mu[\alpha KG]$	-
$v_{D,SUC} = \mu[SUC]$	-
$v_{D,FUM} = \mu[FUM]$	-
$v_{D,MAL} = \mu[MAL]$	-
$v_{D,OAA} = \mu[OAA]$	-
$v_{D,GOX} = \mu[GOX]$	-
$v_{D,6PGL} = \mu[6PGL]$	-
$v_{D,6PG} = \mu[6PG]$	-
$v_{D,KDPG} = \mu[KDPG]$	-
$v_{D,RU5P} = \mu[RU5P]$	-
$v_{D,R5P} = \mu[R5P]$	-
$v_{D,X5P} = \mu[X5P]$	-
$v_{D,S7P} = \mu[S7P]$	-
$v_{D,E4P} = \mu[E4P]$	-
$v_{D,cAMP} = \mu[cAMP]$	-
$v_{D,Glk} = (\mu + k_{degr})[Glk]$	[1]
$v_{D,Pfk} = (\mu + k_{degr})[Pfk]$	[1]
$v_{D,Fbp} = (\mu + k_{degr})[Fbp]$	[1]
$v_{D,Fba} = (\mu + k_{degr})[Fba]$	[1]
$v_{D,Gapdh} = (\mu + k_{degr})[Gapdh]$	[1]
$v_{D,Pyk} = (\mu + k_{degr})[Pyk]$	[1]
$v_{D,Pps} = (\mu + k_{degr})[Pps]$	[1]
$v_{D,Pdh} = (\mu + k_{degr})[Pdh]$	[1]
$v_{D,Acs} = (\mu + k_{degr})[Acs]$	[1]
$v_{D,Cs} = (\mu + k_{degr})[Cs]$	[1]
$v_{D,Icdh} = (\mu + k_{degr})[Icdh]$	[1]
$v_{D,Icdh\text{-}P} = (\mu + k_{degr})[Icdh\text{-}P]$	[1]
$v_{D,\alpha kgdh} = (\mu + k_{degr})[\alpha kgdh]$	[1]
$v_{D,Sdh} = (\mu + k_{degr})[Sdh]$	[1]
$v_{D,Fum} = (\mu + k_{degr})[Fum]$	[1]
$v_{D,Mdh} = (\mu + k_{degr})[Mdh]$	[1]
$v_{D,Mez} = (\mu + k_{degr})[Mez]$	[1]
$v_{D,Pck} = (\mu + k_{degr})[Pck]$	[1]
$v_{D,Ppc} = (\mu + k_{degr})[Ppc]$	[1]
$v_{D,Icl} = (\mu + k_{degr})[Icl]$	[1]
$v_{D,Ms} = (\mu + k_{degr})[Ms]$	[1]
$v_{D,AceK} = (\mu + k_{degr})[AceK]$	[1]

$v_{BM,G6P} = \left(\alpha_{GLC} k_{BM\_GLC\_G6P} + \alpha_{ACE} k_{BM\_ACE\_G6P}\right)[G6P]$	[1]
$v_{BM,F6P} = \left(\alpha_{GLC} k_{BM\_GLC\_F6P} + \alpha_{ACE} k_{BM\_ACE\_F6P}\right)[F6P]$	[1]
$v_{BM,GAP} = \left(\alpha_{GLC} k_{BM\_GLC\_GAP} + \alpha_{ACE} k_{BM\_ACE\_GAP}\right)[GAP]$	[1]
$v_{BM,PEP} = \left(\alpha_{GLC} k_{BM\_GLC\_PEP} + \alpha_{ACE} k_{BM\_ACE\_PEP}\right)[PEP]$	[1]
$v_{BM,PYR} = \left(\alpha_{GLC} k_{BM\_GLC\_PYR} + \alpha_{ACE} k_{BM\_ACE\_PYR}\right)[PYR]$	[1]
$v_{BM,AcCoA} = \left(\alpha_{GLC} k_{BM\_GLC\_AcCoA} + \alpha_{ACE} k_{BM\_ACE\_AcCoA}\right)[AcCoA]$	[1]
$v_{BM,\alpha KG} = \left(\alpha_{GLC} k_{BM\_GLC\_\alpha KG} + \alpha_{ACE} k_{BM\_ACE\_\alpha KG}\right)[\alpha KG]$	[1]
$v_{BM,SUC} = \left(\alpha_{GLC} k_{BM\_GLC\_SUC} + \alpha_{ACE} k_{BM\_ACE\_SUC}\right)[SUC]$	[1]
$v_{BM,FUM} = \left(\alpha_{GLC} k_{BM\_GLC\_FUM} + \alpha_{ACE} k_{BM\_ACE\_FUM}\right)[FUM]$	[1]
$v_{BM,OAA} = \left(\alpha_{GLC} k_{BM\_GLC\_OAA} + \alpha_{ACE} k_{BM\_ACE\_OAA}\right)[OAA]$	[1]
$v_{BM,R5P} = \left(\alpha_{GLC} k_{BM\_GLC\_R5P} + \alpha_{ACE} k_{BM\_ACE\_R5P}\right)[R5P]$	Derived based on [1]
$v_{BM,E4P} = \left(\alpha_{GLC} k_{BM\_GLC\_E4P} + \alpha_{ACE} k_{BM\_ACE\_E4P}\right)[E4P]$	Derived based on [1]

Table S3. Ancillary variables and their equations.

Equation	Unit	Reference
$\alpha_{GLC} = \frac{[GLC^{ex}]}{[GLC^{ex}] + K_{Pts\_GLC}}$	-	[1]
$\alpha_{ACE} = \frac{[ACE^{ex}]}{[ACE^{ex}] + K_{Acs\_ACE}}(1-\alpha_{GLC})$	-	[1]
$\left[H^{+}\right] = 10^{-pH}$	M	-
$[EIIA] = [EIIA_{total}] - [EIIA\text{-}P]$	mM	-
$[Crp\text{-}cAMP] = \frac{[Crp_{total}][cAMP]^{n_{Crp\text{-}cAMP}}}{[cAMP]^{n_{Crp\text{-}cAMP}} + K_{Crp\text{-}cAMP}^{n_{Crp\text{-}cAMP}}}$	mM	Derived based on [1]
$[Crp] = [Crp_{total}] - [Crp\text{-}cAMP]$	mM	-
$[Cra\text{-}FBP] = \frac{[Cra_{total}][FBP]^{n_{Cra\text{-}FBP}}}{[FBP]^{n_{Cra\text{-}FBP}} + K_{Cra\text{-}FBP}^{n_{Cra\text{-}FBP}}}$	mM	Derived based on [1]
$[Cra] = [Cra_{total}] - [Cra\text{-}FBP]$	mM	-
$[PdhR\text{-}PYR] = \frac{[PdhR_{total}][PYR]^{n_{PdhR\text{-}PYR}}}{[PYR]^{n_{PdhR\text{-}PYR}} + K_{PdhR\text{-}PYR}^{n_{PdhR\text{-}PYR}}}$	mM	Derived based on [1]
$[PdhR] = [PdhR_{total}] - [PdhR\text{-}PYR]$	mM	-
$OP_{NADH} = \left(v_{E,Gapdh} + v_{E,Pdh} + v_{E,\alpha kgdh} + v_{E,Mdh}\right) \times (P/O)$	mM h^{-1}	[9]
$OP_{FADH_2} = v_{E,Sdh} \times (P/O)'$	mM h^{-1}	[9]
$v_{ATP} = OP_{NADH} + OP_{FADH_2} - v_{E,Glk} - v_{E,Pfk} + v_{E,Gapdh} + v_{E,Pyk} - v_{E,Pps} + v_{E,Ack} - v_{E,Acs} + v_{E,\alpha kgdh} - v_{E,Pck} - v_{E,AceK\text{-}ki} - v_{E,Cya}$	mM h^{-1}	Developed based on [9]
$\mu = k_{ATP} v_{ATP}$	h^{-1}	Assumed

Table S4. Model variables (Class I: Measured, Class II: Estimated by reference, ClassIII: Assumed by this paper, '-': Fixed, i.e. not optimized).

Name	Description	Initital value	Unit	Class	Reference	Optimized value (used in simulation)
X	Biomass	0.012	$g_{DW}\ L_{Culture}^{-1}$	-	[10]	0.012
GLC^{ex}	External glucose	22.5	mM	-	[10]	22.5
GLC	Internal glucose	3.48	mM	III	Assumed	2.85
G6P	Glucose-6-phosphate	3.48	mM	I	[4]	3.48
F6P	Fructose-6-phosphate	0.6	mM	I	[4]	0.57
FBP	Fructose-1,6-bisphosphate	15	mM	I	[11]	15.03
GAP	Glycelaldehyde-3-phosphate	0.218	mM	I	[4]	0.218
PEP	Phosphoenol pyruvate	0.18	mM	I	[11]	0.40
PYR	Pyruvate	2.67	mM	I	[4]	1.77
AcCoA	Acetyl-CoA	0.61	mM	I	[11]	0.612
AcP	Acetyl phosphate	1.1	mM	I	[11]	1.10
ACE^{ex}	External acetate	0	mM	-	[10]	0
ICIT	Isocitrate	0.17	mM	I	[12]	0.11
αKG	α-Ketoglutarate	0.44	mM	I	[11]	0.44
SUC	Succinate	0.57	mM	I	[11]	0.57
FUM	Fumarate	0.12	mM	I	[11]	0.12
MAL	Malate	1.7	mM	I	[11]	1.43
OAA	Oxaloacetate	0.68	mM	I	[12]	0.65
GOX	Glyoxylate	0.10	mM	II	[13]	0.08
6PGL	6-Phosphogluconolactone	1	mM	III	Assumed	1.61
6PG	6-Phosphogluconate	3.8	mM	I	[11]	3.46
KDPG	2-Keto-3-deoxy-6-phosphogluconate	1	mM	III	Assumed	1.04
RU5P	Ribulose-5-phosphate	0.111	mM	II	[4]	0.16
R5P	Ribose-5-phosphate	0.398	mM	II	[4]	0.398
X5P	Xylulose-5-phosphate	0.138	mM	II	[4]	0.188
S7P	Sedoheptulose-7-phosphate	0.276	mM	II	[4]	0.066

E4P	Erythrose-4-phosphate	0.098	mM	II	[4]	0.097
cAMP	Cyclic AMP	0.035	mM	I	[11]	0.019
EIIA-P	Phosphorylated Pts protein EIIA	0.0731	mM	II	[13]	0.014
Glk	Glucokinase	1.42e-03	mM	III	Assumed	3.0e-03
Pfk	Phosphofructokinase	1.42e-03	mM	I	[14]	1.4e-03
Fbp	Fructose-1,6-bisphosphatase	2.55e-04	mM	I	[15]	2.97e-04
Fba	Fructose-1,6-bisphosphate aldolase class II	0.0309	mM	I	[16]	0.023
Gapdh	Glyceraldehyde 3-phosphate dehydrogenase	0.0356	mM	I	[17]	0.040
Pyk	Pyruvate kinase I	2.75e-03	mM	I	[18]	2.88e-03
Pps	Phosphoenolpyruvate synthase	3.86e-03	mM	I	[19]	3.86e-03
Pdh	Pyruvate dehydrogenase	3.32e-04	mM	I	[20]	5.30e-04
Acs	Acetyl coenzyme A synthetase	2.84e-04	mM	II	[1]	1.68e-04
Cs	Citrate synthase	3.68e-03	mM	I	[21]	2.78e-03
Icdh	Isocitrate dehydrogenase	0.119	mM	I	[22]	0.172
Icdh-P	phosphorylated isocitrate dehydrogenase	0.119	mM	I	[22]	0.119
αkgdh	α-Keto-D-gluconate dehydrogenase	1.76e-03	mM	I	[23]	2.6e-03
Sdh	Succinate dehydrogenase	0.0508	mM	I	[24]	0.059
Fum	Fumarase	2.10e-03	mM	I	[25]	2.1e-03
Mdh	Malate dehydrogenase	0.0144	mM	I	[26]	7.3e-03
Mez	Malic enzyme	1.87e-04	mM	I	[27]	1.87e-04
Pck	Phosphoenolpyruvate carboxykinase	1.08e-03	mM	I	[28]	6.7e-04
Ppc	Phosphoenolpyruvate carboxylase	1.89e-06	mM	I	[29]	3.42e-06
Icl	Isocitrate Lyase	0.0120	mM	I	[30]	0.012
Ms	Malate synthase	3.61e-03	mM	I	[31]	3.62e-03
AceK	Isocitrate dehydrogenase phosphatase/kinase	3.61e-05	mM	I	[31]	2.86e-05

Table S5. Constant components (Class I: Measured, Class II: Estimated by reference, ClassIII: Assumed by this paper, '-': Fixed, i.e. not optimized).

Name	Description	Value	Unit	Class	Reference
GLC^{feed}	Glucose concentration in feeding medium	0 for batch culture and 22.2 for continuous culture	mM	-	[10]
ATP	Adenosine-5-triphosphate	9.6	mM	-	[11]
ADP	Adenosine-5-diphosphate	0.56	mM	-	[11]
AMP	Adenosine-5-monophosphate	0.28	mM	-	[11]
NAD	Nicotinamide adenine dinucleotide	2.6	mM	-	[11]
NADH	Nicotinamide adenine dinucleotide reduced	0.083	mM	-	[11]
NADP	Dihydronicotinamide adenine dinucleotide phosphate	2.1e-03	mM	-	[11]
NADPH	Dihydronicotinamide adenine dinucleotide phosphate reduced	0.12	mM	-	[11]
CoA	Coenzyme A	1.4	mM	-	[11]
P_i	Inorganic phosphate	10	mM	-	[32]
$EIIA_{total}$	Total concentration of EIIA	0.0769	mM	-	[33]
Crp_{total}	Total concentration of Crp	0.0115	mM	-	[34]
Cra_{total}	Total concentration of Cra	3.00e-04	mM	-	[13]
$PdhR_{total}$	Total concentration of PdhR	6.66e-05	mM	-	[35]
$IclR_{total}$	Total concentration of IclR	8.30e-05	mM	-	[13]
pH	Intracellular pH	7.5	-	-	[8]
aceBAK_DNA	DNA concentration	5.15e-07	mM	-	[36]

Table S6. Kinetic parameters (Class I: Measured, Class II: Estimated by reference, ClassIII: Assumed by this paper, '-': Fixed, i.e. not optimized).

Reaction involved	Parameter	Value	Unit	Class	Reference	Optimized value (used in simulation)
v_{Pts1}	k_{Pts1}	3.70e+04	$mM^{-1}\ h^{-1}$	II	[1]	5.59e+04
	km_{Pts1}	1.48e+04	$mM^{-1}\ h^{-1}$	II	[1]	2.77e+04
v_{Pts4}	v_{Pts4}^{max}	1.54e+04	$mM\ h^{-1}$	II	[1]	3.94e+03
	K_{Pts_EIIA}	0.240	mM	II	[1]	2.11e-03
	K_{Pts_GLC}	6.67e-03	mM	II	[1]	4.89e-03
v_{Nonpts}	v_{Nonpts}^{max}	931	$mM\ h^{-1}$	II	[2]	3.99e+03
	K_{Nonpts_S}	0.846	mM	II	[2]	1.55
	K_{Nonpts_I}	5.64e-04	mM	II	[2]	0.01
$v_{E,Glk}$	k_{Glk_cat}	1.26e+06	h^{-1}	II	[3]	2.01e+06
	$K_{Glk\_GLC\_m}$	0.22	mM	I	[37]	0.15
	$K_{Glk\_ATP\_m}$	0.8	mM	I	[37]	0.71
	$K_{Glk\_G6P\_i}$	15	mM	I	[37]	14.98
$v_{E,Pgi}$	v_{Pgi}^{max}	2.34e+06	$mM\ h^{-1}$	II	[4]	3.56e+06
	K_{Pgi_eq}	0.43	-	I	[38]	1.44
	K_{Pgi_G6P}	2.46	mM	I	[39]	2.46
	K_{Pgi_F6P}	0.2	mM	I	[40]	0.34
	$K_{Pgi\_F6P\_6pginh}$	0.2	mM	I	[41]	0.19
	$K_{Pgi\_G6P\_6pginh}$	0.2	mM	I	[41]	0.18
$v_{E,Pfk}$	k_{Pfk_cat}	4.67e+09	h^{-1}	II	[4]	2.49e+10
	K_{Pfk_PEP}	3.26	mM	II	[4]	1.73
	$K_{Pfk\_ADP\_b}$	0.25	mM	I	[42]	0.26
	$K_{Pfk\_AMP\_b}$	0.01	mM	I	[42]	0.03
	$K_{Pfk\_ADP\_a}$	239	mM	I	[42]	276.95
	$K_{Pfk\_AMP\_a}$	8.74	mM	I	[42]	10.05
	$K_{Pfk\_ATP\_s}$	0.16	mM	I	[43]	0.16
	$K_{Pfk\_ADP\_c}$	0.36	mM	I	[42]	0.45
	$K_{Pfk\_F6P\_s}$	0.14	mM	I	[43]	0.02
	L_{Pfk}	4e+06	-	I	[44]	1.77e+06
	n_{Pfk}	4	-	-	[44]	4
$v_{E,Fbp}$	k_{Fbp_cat}	8.17e+06	h^{-1}	II	[1]	7.86e+06
	K_{Fbp_FBP}	1.70e-03	mM	II	[1]	8.92e-03
	K_{Fbp_PEP}	0.169	mM	II	[1]	0.49
	L_{Fbp}	4.00e+06	-	II	[1]	4.41e+06
	n_{Fbp}	4	-	-	[1]	4
$v_{E,Fba}$	k_{Fba_cat}	2.03e+06	h^{-1}	II	[4]	6.95e+06
	K_{Fba_eq}	0.14	mM	I	[38]	0.37
	K_{Fba_FBP}	0.133	mM	I	[45]	0.084
	K_{Fba_GAP}	0.088	mM	I	[45]	0.154
	K_{Fba_DHAP}	0.088	mM	I	[45]	0.0884
	V_{Fba_blf}	2	-	I	[45]	1.54
	$K_{Fba\_GAP\_inh}$	0.6	mM	I	[45]	0.6
$v_{E,Gapdh}$	k_{Gapdh_cat}	9.32e+07	h^{-1}	II	[4]	5.04e+07
	K_{Gapdh_eq}	0.63	-	I	[38]	0.30
	K_{Gapdh_GAP}	0.15	mM	I	[46]	0.15
	K_{Gapdh_PGP}	0.1	mM	I	[46]	0.13
	K_{Gapdh_NAD}	0.45	mM	I	[46]	0.45
	K_{Gapdh_NADH}	0.02	mM	I	[46]	0.02
$v_{E,Pyk}$	k_{Pyk_cat}	8.00e+04	h^{-1}	II	[4]	8.16e+04
	K_{Pyk_PEP}	0.31	mM	I	[47]	0.31
	K_{Pyk_FBP}	0.19	mM	I	[47]	0.25
	K_{Pyk_AMP}	0.2	mM	II	[4]	0.26

	K_{Pyk_ADP}	0.26	mM	I	[47]	0.21
	K_{Pyk_ATP}	22.5	mM	I	[47]	20.17
	L_{Pyk}	1e+03	-	I	[47]	997.10
	n_{Pyk}	4	-	-	[47]	4
$v_{E,Pps}$	k_{Pps_cat}	399	h^{-1}	II	[1]	2.49e+03
	K_{Pps_PYR}	9.98e-04	mM	II	[1]	7.13e-04
	K_{Pps_PEP}	5.64e-04	mM	II	[1]	2.16e-04
	L_{Pps}	1.00e-79	-	II	[1]	1.04e-79
	n_{Pps}	2	-	-	[1]	2
$v_{E,Pdh}$	k_{Pdh_cat}	4.68e+07	h^{-1}	II	[5]	4.83e+07
	K_{Pdh_i}	46.4	-	II	[5]	68.34
	$K_{Pdh\_PYR\_m}$	1	mM	I	[48]	1
	$K_{Pdh\_NAD\_m}$	0.4	mM	I	[49]	0.4
	$K_{Pdh\_NADH\_m}$	0.1	mM	II	[5]	0.05
	$K_{Pdh\_CoA\_m}$	0.014	mM	II	[5]	0.005
	$K_{Pdh\_AcCoA\_m}$	0.008	mM	I	[50]	0.008
$v_{E,Pta}$	v_{Pta}^{max}	2.52e+03	mM h^{-1}	I	[51]	5.36e+03
	K_{Pta_eq}	0.0281	-	I	[52]	0.028
	$K_{Pta\_AcCoA\_i}$	0.2	mM	II	[5]	0.2
	$K_{Pta\_CoA\_i}$	0.029	mM	II	[5]	0.08
	$K_{Pta\_Pi\_m}$	2.6	mM	II	[5]	0.69
	$K_{Pta\_Pi\_i}$	2.6	mM	II	[5]	2.11
	$K_{Pta\_AcP\_m}$	0.7	mM	II	[5]	0.23
	$K_{Pta\_AcP\_i}$	0.2	mM	II	[5]	0.32
$v_{E,Ack}$	v_{Ack}^{max}	1.62e+05	mM h^{-1}	I	[51]	1.95e+05
	K_{Ack_eq}	174.2	-	I	[52]	233.912
	$K_{Ack\_ADP\_m}$	0.5	mM	II	[5]	0.18
	$K_{Ack\_AcP\_m}$	0.16	mM	II	[5]	0.05
	$K_{Ack\_ACE\_m}$	7	mM	II	[5]	6.09
	$K_{Ack\_ATP\_m}$	0.07	mM	I	[53]	0.09
$v_{E,Acs}$	k_{Acs_cat}	8.81e+04	h^{-1}	II	[1]	1.29e+05
	K_{Acs_ACE}	0.0167	mM	II	[1]	0.024
$v_{E,Cs}$	k_{Cs_cat}	2.79e+05	h^{-1}	II	[1]	5.43e+05
	$K_{Cs\_\alpha KG}$	0.355	mM	II	[1]	0.19
	$K_{Cs\_OAA\_AcCoA}$	0.0164	mM	II	[1]	0.009
	K_{Cs_AcCoA}	0.120	mM	II	[1]	0.03
	K_{Cs_OAA}	0.0164	mM	II	[1]	0.018
$v_{E,Icdh}$	k_{Icdh_cat}	1.14e+05	h^{-1}	II	[1]	8.58e+04
	K_{Icdh_ICIT}	9.02e-05	mM	II	[1]	2.01e-04
	K_{Icdh_PEP}	0.188	mM	II	[1]	0.05
	L_{Icdh}	127	-	II	[1]	92.58
	n_{Icdh}	2	-	-	[1]	2
$v_{E,\alpha kgdh}$	$k_{\alpha kgdh\_cat}$	2.59e+08	h^{-1}	II	[6]	7.02e+08
	$K_{\alpha kgdh\_NAD\_m}$	0.07	mM	I	[54]	0.06
	$K_{\alpha kgdh\_CoA\_m}$	0.002	mM	I	[54]	0.003
	$K_{\alpha kgdh\_\alpha KG\_m}$	1	mM	I	[54]	0.24
	$K_{\alpha kgdh\_Z}$	1.5	mM	II	[6]	4.17
	$K_{\alpha kgdh\_SUC\_I}$	1	mM	II	[6]	2.07
	$K_{\alpha kgdh\_NADH\_I}$	0.018	mM	I	[54]	0.018
	$K_{\alpha kgdh\_\alpha KG\_I}$	0.75	mM	II	[6]	1
$v_{E,Sdh}$	k_{Sdh1_cat}	3.72e+03	h^{-1}	II	[6]	1.96e+04
	k_{Sdh2_cat}	3.72e+03	h^{-1}	II	[6]	1.96e+04
	K_{Sdh_eq}	10	-	II	[6]	29.66
	$K_{Sdh\_SUC\_m}$	0.22	mM	I	[55]	0.17
$v_{E,Fum}$	k_{Fum1_cat}	7.34e+05	h^{-1}	II	[6]	3.82e+05
	k_{Fum2_cat}	7.34e+05	h^{-1}	II	[6]	3.82e+05
	K_{Fum_eq}	10	-	II	[6]	12.87
	$K_{Fum\_FUM\_m}$	0.1	mM	II	[6]	0.09
$v_{E,Mdh}$	k_{Mdh1_cat}	3.24e+05	h^{-1}	II	[6]	4.60e+05
	k_{Mdh2_cat}	3.24e+05	h^{-1}	II	[6]	4.60e+05
	K_{Mdh_eq}	1	-	II	[6]	0.75
	$K_{Mdh\_NAD\_m}$	0.1	mM	I	[56]	0.05

	$K_{Mdh\_NAD\_I}$	0.31	mM	I	[56]	0.11
	$K_{Mdh\_NAD\_II}$	0.31	mM	II	[6]	0.95
	$K_{Mdh\_MAL\_m}$	1.33	mM	I	[56]	1.77
	$K_{Mdh\_MAL\_I}$	3.30	mM	II	[6]	3.46
	$K_{Mdh\_OAA\_m}$	0.27	mM	I	[56]	0.21
	$K_{Mdh\_OAA\_I}$	0.27	mM	II	[6]	0.34
	$K_{Mdh\_OAA\_II}$	0.17	mM	II	[6]	0.07
	$K_{Mdh\_NADH\_m}$	0.04	mM	I	[56]	0.03
	$K_{Mdh\_NADH\_I}$	0.04	mM	II	[6]	0.02
$v_{E,Mez}$	k_{Mez_cat}	5.58e+05	h^{-1}	II	[1]	3.68e+05
	K_{Mez_MAL}	3.50e-03	mM	II	[1]	1.46e-03
	K_{Mez_AcCoA}	2.05	mM	II	[1]	1.82
	K_{Mez_cAMP}	3.69	mM	II	[1]	4.55
	L_{Mez}	1.04e+05	-	II	[1]	2.39e+05
	n_{Mez}	1.33	-	II	[1]	1.99
$v_{E,Pck}$	k_{Pck_cat}	2.36e+06	h^{-1}	II	[9]	3.44e+06
	K_{Pck_OAA}	0.67	mM	I	[57]	0.58
	$K_{Pck\_ATP\_i}$	0.04	mM	I	[57]	0.04
	$K_{Pck\_ADP\_i}$	0.04	mM	II	[7]	0.02
	K_{Pck_PEP}	0.07	mM	I	[57]	0.07
	$K_{Pck\_PEP\_i}$	0.06	mM	I	[57]	0.06
	$K_{Pck\_OAA\_I}$	0.45	mM	II	[7]	0.35
	$K_{Pck\_ATP\_I}$	0.04	mM	I	[57]	0.04
$v_{E,Ppc}$	k_{Ppc_cat}	5.31e+06	h^{-1}	II	[1]	5.39e+06
	K_{Ppc_PEP}	0.0271	mM	II	[1]	0.03
	K_{Ppc_FBP}	0.230	mM	II	[1]	0.18
	L_{Ppc}	5.2e+06	-	II	[1]	5.65e+06
	n_{Ppc}	3	-	-	[1]	3
$v_{E,Icl}$	k_{Icl_cat}	1.76e+06	h^{-1}	II	[1]	9.42+05
	K_{Icl_ICIT}	0.0124	mM	II	[1]	0.02
	K_{Icl_PEP}	0.0310	mM	II	[1]	0.02
	K_{Icl_3PG}	0.406	mM	II	[1]	0.52
	$K_{Icl\_\alpha KG}$	0.466	mM	II	[1]	0.83
	L_{Icl}	5.01e+04	-	II	[1]	1.91e+05
	n_{Icl}	4	-	-	[1]	4
$v_{E,Ms}$	k_{Ms_cat}	1.04e+04	h^{-1}	II	[1]	1.70e+04
	$K_{Ms\_GOX\_AcCoA}$	0.406	mM	II	[1]	0.39
	K_{Ms_AcCoA}	0.426	mM	II	[1]	0.46
	K_{Ms_GOX}	0.536	mM	II	[1]	1.11
$v_{E,AceK\text{-}ki}$	$k_{AceK\_ki\_cat}$	1.22e+16	h^{-1}	II	[1]	5.27e+15
$v_{E,AceK\text{-}ph}$	$k_{AceK\_ph\_cat}$	6.12e+12	h^{-1}	II	[1]	1.82e+12
	K_{AceK_ICDH}	0.530	mM	II	[1]	0.19
	$K_{AceK\_ICDH\text{-}P}$	7.93	mM	II	[1]	7.26
	K_{AceK_ICIT}	0.0773	mM	II	[1]	0.09
	K_{AceK_GOX}	0.488	mM	II	[1]	0.50
	K_{AceK_OAA}	0.0976	mM	II	[1]	0.07
	$K_{AceK\_\alpha KG}$	0.463	mM	II	[1]	0.42
	K_{AceK_PEP}	0.304	mM	II	[1]	0.36
	K_{AceK_3PG}	0.886	mM	II	[1]	0.42
	K_{AceK_PYR}	0.0214	mM	II	[1]	0.03
	L_{AceK}	1.00e+08	-	II	[1]	2.83e+08
	n_{AceK}	2	-	-	[1]	2
$v_{E,G6pdh}$	v_{G6pdh}^{max}	4.97e+03	mM h^{-1}	II	[4]	1.66e+04
	K_{G6pdh_G6P}	0.07	mM	I	[58]	0.07
	$K_{G6pdh\_NADPH\_g6pinh}$	0.18	mM	I	[58]	0.19
	K_{G6pdh_NADP}	0.015	mM	I	[58]	0.004
	$K_{G6pdh\_NADPH\_nadpinh}$	0.01	mM	I	[58]	0.08
$v_{E,Pgl}$	v_{Pgl}^{max}	4.5e+04	mM h^{-1}	II	[8]	2.28e+04
	K_{Pgl_eq}	42.8	-	I	[59]	42.69
	$K_{Pgl\_6PGL\_m}$	0.023	mM	I	[37]	0.023
	$K_{Pgl\_6PG\_m}$	10	mM	I	[37]	10.01

	K_{Pgl_h1}	5.61e-03	mM	I	[60]	0.004
	K_{Pgl_h2}	9.73e-06	mM	I	[60]	9.70e-06
$v_{E,Edd}$	v_{Edd}^{max}	300	mM h^{-1}	II	[8]	515.41
	K_{Edd_eq}	1e+03	-	I	[61]	1.01e+03
	$K_{Edd\_6PG\_m}$	0.6	mM	I	[61]	0.12
	$K_{Edd\_KDPG\_m}$	1	mM	II	[8]	2.02
	pH_{Edd_m}	6.4	-	I	[61]	7.53
	pK_{Edd}	10	-	II	[8]	8.74
$v_{E,Eda}$	v_{Eda}^{max}	300	mM h^{-1}	II	[8]	667.33
	K_{Eda_eq}	0.5	-	I	[62]	0.50
	$K_{Eda\_PYR\_m}$	10	mM	II	[8]	7.69
	$K_{Eda\_KDPG\_m}$	0.35	mM	I	[62]	0.15
	$K_{Eda\_GAP\_m}$	1	mM	II	[8]	1.18
	pH_{Eda_m}	7.5	-	I	[62]	10.38
	pK_{Eda}	10	-	II	[8]	36.97
$v_{E,6Pgdh}$	v_{6Pgdh}^{max}	5.84e+04	mM h^{-1}	II	[4]	2.48e+05
	K_{6Pgdh_6PG}	0.1	mM	I	[63]	0.10
	K_{6Pgdh_NADP}	0.028	mM	I	[63]	0.02
	$K_{6Pgdh\_NADPH\_inh}$	0.01	mM	I	[63]	0.04
	$K_{6Pgdh\_ATP\_inh}$	3	mM	I	[63]	3.01
$v_{E,R5pi}$	v_{R5pi}^{max}	1.74e+04	h^{-1}	II	[4]	3.21e+04
	K_{R5pi_eq}	4	-	I	[64]	0.48
$v_{E,Ru5p}$	v_{Ru5p}^{max}	2.42e+04	h^{-1}	II	[4]	1.27e+04
	K_{Ru5p_eq}	1.4	-	I	[64]	1.41
$v_{E,TktA}$	v_{TktA}^{max}	3.41e+04	mM^{-1} h^{-1}	II	[4]	8.87e+03
	K_{TktA_eq}	1.2	-	I	[64]	1.20
$v_{E,TktB}$	v_{TktB}^{max}	3.12e+05	mM^{-1} h^{-1}	II	[4]	3.79e+05
	K_{TktB_eq}	10	-	I	[64]	9.97
$v_{E,Tal}$	v_{Tal}^{max}	3.91e+04	mM^{-1} h^{-1}	II	[4]	7.17e+04
	K_{Tal_eq}	1.05	-	I	[64]	1.05
$v_{E,Cya}$	v_{Cya}^{max}	28.9	mM h^{-1}	II	[1]	9.45
	$K_{Cya\_EIIA\text{-}P}$	0.0479	mM	II	[1]	0.002
$v_{E,cAMP_{degr}}$	$v_{cAMP_{degr}}^{max}$	2.03	mM h^{-1}	II	[1]	9.21
	$K_{cAMPdegr\_cAMP}$	0.0564	mM	II	[1]	0.048
$v_{G,glk}$	K_{glk_Cra}	9.35e-06	mM	II	[1]	1.22e-08
	$v_{glk\_Cra\_unbound}$	0.0812	mM h^{-1}	II	[1]	0.206
	$v_{glk\_Cra\_bound}$	6.38e-04	mM h^{-1}	II	[1]	0.002
$v_{G,pfkA}$	K_{pfkA_Cra}	9.35e-06	mM	II	[1]	9.87e-09
	$v_{pfkA\_Cra\_unbound}$	0.0812	mM h^{-1}	II	[1]	0.05
	$v_{pfkA\_Cra\_bound}$	6.38e-04	mM h^{-1}	II	[1]	0.001
$v_{G,fbp}$	K_{fbp_Cra}	0.0175	mM	II	[1]	3.75e-05
	$v_{fbp\_Cra\_unbound}$	0	mM h^{-1}	II	[1]	0
	$v_{fbp\_Cra\_bound}$	1.07e-03	mM h^{-1}	II	[1]	0.001
$v_{G,fbaA}$	K_{fbaA_Cra}	1.34	mM	II	[1]	3.26e-03
	$v_{fbaA\_Cra\_unbound}$	0.0310	mM h^{-1}	II	[1]	0.025
	$v_{fbaA\_Cra\_bound}$	0	mM h^{-1}	II	[1]	0
	K_{fbaA_Crp}	0.286	mM	II	[1]	0.009
	$v_{fbaA\_Crp\_unbound}$	0	mM h^{-1}	II	[1]	0
	$v_{fbaA\_Crp\_bound}$	0.0239	mM h^{-1}	II	[1]	0.016
$v_{G,gapA}$	K_{gapA_Cra}	1.34	mM	II	[1]	0.02
	$v_{gapA\_Cra\_unbound}$	0.0349	mM h^{-1}	II	[1]	0.02
	$v_{gapA\_Cra\_bound}$	0	mM h^{-1}	II	[1]	0
	K_{gapA_Crp}	0.286	mM	II	[1]	0.05
	$v_{gapA\_Crp\_unbound}$	0	mM h^{-1}	II	[1]	0
	$v_{gapA\_Crp\_bound}$	0.0269	mM h^{-1}	II	[1]	0.03
$v_{G,pykF}$	K_{pykF_Cra}	0.0341	mM	II	[1]	7.26e-05
	$v_{pykF\_Cra\_unbound}$	6.40e-03	mM h^{-1}	II	[1]	0.011
	$v_{pykF\_Cra\_bound}$	3.52e-05	mM h^{-1}	II	[1]	4.30e-05
$v_{G,ppsA}$	K_{ppsA_Cra}	0.252	mM	II	[1]	4.88e-04
	$v_{ppsA\_Cra\_unbound}$	0	mM h^{-1}	II	[1]	0
	$v_{ppsA\_Cra\_bound}$	0.0798	mM h^{-1}	II	[1]	0.053

$v_{G,pdh}$	K_{pdh_PdhR}	0.0652	mM	II	[1]	2.46e-05
	$v_{pdh\_PdhR\_unbound}$	1.57e-03	mM h^{-1}	II	[1]	0.001
	$v_{pdh\_PdhR\_bound}$	5.70e-06	mM h^{-1}	II	[1]	7.50e-06
$v_{G,acs}$	K_{acs_Crp}	0.112	mM	II	[1]	1.36e-03
	$v_{acs\_Crp\_unbound}$	0	mM h^{-1}	II	[1]	0
	$v_{acs\_Crp\_bound}$	1.13e-03	mM h^{-1}	II	[1]	3.62e-04
	n_{acs}	2.31	-	-	[1]	2.31
$v_{G,gltA}$	K_{gltA_Crp}	0.954	mM	II	[1]	0.056
	$v_{gltA\_Crp\_unbound}$	0	mM h^{-1}	II	[1]	0
	$v_{gltA\_Crp\_bound}$	0.0275	mM h^{-1}	II	[1]	0.03
	n_{gltA}	1.07	-	-	[1]	1.07
$v_{G,icdA}$	K_{icdA_Cra}	0.0174	mM	II	[1]	2.92e-05
	$v_{icdA\_Cra\_unbound}$	4.88e-03	mM h^{-1}	II	[1]	0.01
	$v_{icdA\_Cra\_bound}$	0.0377	mM h^{-1}	II	[1]	0.02
$v_{G,sucAB}$	K_{sucAB_Crp}	2.17	mM	II	[1]	0.31
	$v_{sucAB\_Crp\_unbound}$	0	mM h^{-1}	II	[1]	0
	$v_{sucAB\_Crp\_bound}$	0.0271	mM h^{-1}	II	[1]	0.02
	n_{sucAB}	0.74	-	-	[1]	0.74
$v_{G,sdhCDAB}$	$K_{sdhCDAB\_Crp}$	2.17	mM	II	[1]	0.086
	$v_{sdhCDAB\_Crp\_unbound}$	0	mM h^{-1}	II	[1]	0
	$v_{sdhCDAB\_Crp\_bound}$	0.199	mM h^{-1}	II	[1]	0.24
	$n_{sdhCDAB}$	0.74	-	-	[1]	0.74
$v_{G,fumABC}$	K_{fumABC_Crp}	2.17	mM	II	[1]	0.15
	$v_{fumABC\_Crp\_unbound}$	0	mM h^{-1}	II	[1]	0
	$v_{fumABC\_Crp\_bound}$	0.0462	mM h^{-1}	II	[1]	0.05
	n_{fumABC}	0.74	-	-	[1]	0.74
$v_{G,mdh}$	K_{mdh_Crp}	1.43	mM	II	[1]	0.21
	$v_{mdh\_Crp\_unbound}$	0	mM h^{-1}	II	[1]	0
	$v_{mdh\_Crp\_bound}$	0.0816	mM h^{-1}	II	[1]	0.10
$v_{G,maeB}$	SS_{Mez}^{GLC}	6.84e-03	mM	II	[1]	5.00e-03
	SS_{Mez}^{ACE}	0.0233	mM	II	[1]	0.015
$v_{G,pckA}$	K_{pckA_Cra}	0.0794	mM	II	[1]	1.52e-04
	$v_{pckA\_Cra\_unbound}$	0	mM h^{-1}	II	[1]	0
	$v_{pckA\_Cra\_bound}$	0.0137	mM h^{-1}	II	[1]	0.014
$v_{G,ppc}$	SS_{Ppc}^{GLC}	2.15e-03	mM	II	[1]	4.93e-03
	SS_{Ppc}^{ACE}	6.04e-04	mM	II	[1]	1.47e-03
$v_{G,aceA}$	K_{aceBAK_Cra}	0.0542	mM	II	[1]	5.4e-04
	$v_{aceBAK\_Cra\_unbound}$	1.02e-04	mM h^{-1}	II	[1]	1.76e-04
	$v_{aceBAK\_Cra\_bound}$	0.107	mM h^{-1}	II	[1]	0.05
	K_{aceBAK_Crp}	8.14	mM	II	[1]	0.53
	$v_{aceBAK\_Crp\_unbound}$	1.72e-03	mM h^{-1}	II	[1]	3.47e-03
	$v_{aceBAK\_Crp\_bound}$	1.98e-05	mM h^{-1}	II	[1]	2.18e-05
	K_{aceBAK_DNA}	1.13e-06	mM	II	[1]	9.51e-07
	K_{aceBAK_PYR}	0.506	mM	II	[1]	2.01
	$K_{aceBAK\_PYRprime}$	1.70e-03	mM	II	[1]	2.723e-03
	K_{aceBAK_GOX}	2.75e-03	mM	II	[1]	2.246e-03
	$k_{aceBAK\_cat\_IclR}$	3.35	h^{-1}	II	[1]	4.41
	L_{aceBAK}	923	-	II	[1]	412.07
$v_{G,aceB}$	$Factor_{aceB}$	0.3	-	II	[1]	0.31
$v_{G,aceK}$	$Factor_{aceK}$	0.03	-	II	[1]	0.02
$v_{G,\bullet}$	k_{expr}	5.56	h	II	[1]	7.26
$v_{D,\bullet}$	k_{degr}	1.8	h^{-1}	I	[65]	0.33
$v_{BM,G6P}$	$k_{BM\_GLC\_G6P}$	554	h^{-1}	II	[1]	48.92
	$k_{BM\_ACE\_G6P}$	274	h^{-1}	II	[1]	456.46
$v_{BM,F6P}$	$k_{BM\_GLC\_F6P}$	554	h^{-1}	III	Assumed	1.27e+03
	$k_{BM\_ACE\_F6P}$	274	h^{-1}	III	Assumed	368.62
$v_{BM,GAP}$	$k_{BM\_GLC\_GAP}$	176	h^{-1}	III	Assumed	101.66
	$k_{BM\_ACE\_GAP}$	238	h^{-1}	III	Assumed	420.74
$v_{BM,PEP}$	$k_{BM\_GLC\_PEP}$	1.52e+03	h^{-1}	II	[1]	957.04
	$k_{BM\_ACE\_PEP}$	169	h^{-1}	II	[1]	253.05
$v_{BM,PYR}$	$k_{BM\_GLC\_PYR}$	1.99e+03	h^{-1}	II	[1]	850.96
	$k_{BM\_ACE\_PYR}$	1.87e+04	h^{-1}	II	[1]	1.24e+04
$v_{BM,AcCoA}$	$k_{BM\_GLC\_AcCoA}$	6.77e+03	h^{-1}	II	[1]	2.37e+03

	$k_{BM\_ACE\_AcCoA}$	389	h^{-1}	II	[1]	763.07
$v_{BM,\alpha KG}$	$k_{BM\_GLC\_\alpha KG}$	3.52e+03	h^{-1}	II	[1]	3.00e+03
	$k_{BM\_ACE\_\alpha KG}$	202	h^{-1}	II	[1]	323.912
$v_{BM,SUC}$	$k_{BM\_GLC\_SUC}$	3.52e+03	h^{-1}	III	Assumed	1.89e+03
	$k_{BM\_ACE\_SUC}$	202	h^{-1}	III	Assumed	227.03
$v_{BM,FUM}$	$k_{BM\_GLC\_FUM}$	3.52e+03	h^{-1}	III	Assumed	3.47e+03
	$k_{BM\_ACE\_FUM}$	202	h^{-1}	III	Assumed	288.72
$v_{BM,OAA}$	$k_{BM\_GLC\_OAA}$	2.30e+04	h^{-1}	II	[1]	2.31e+04
	$k_{BM\_ACE\_OAA}$	5.15e+03	h^{-1}	II	[1]	1.31e+04
$v_{BM,R5P}$	$k_{BM\_GLC\_R5P}$	554	h^{-1}	III	Assumed	307.97
	$k_{BM\_ACE\_R5P}$	274	h^{-1}	III	Assumed	499.54
$v_{BM,E4P}$	$k_{BM\_GLC\_E4P}$	554	h^{-1}	III	Assumed	1.51e+03
	$k_{BM\_ACE\_E4P}$	274	h^{-1}	III	Assumed	289.66
Others	$K_{Crp\text{-}cAMP}$	0.505	mM	II	[1]	0.420
	$n_{Crp\text{-}cAMP}$	1	-	-	[1]	1
	$K_{Cra\text{-}FBP}$	0.767	mM	II	[1]	0.029
	$n_{Cra\text{-}FBP}$	2	-	-	[1]	2
	$K_{PdhR\text{-}PYR}$	0.0925	mM	II	[1]	0.043
	$n_{PdhR\text{-}PYR}$	1	-	-	[1]	1
	(P/O) (P/O ratio for NADH)	2.5	-	I	[2]	3.48
	(P/O)' (P/O ratio for $FADH_2$)	1.5	-	I	[2]	1.49
	k_{ATP}	8.87e-06	mM^{-1}	I	[9]	1.32e-05
	ρ (Cell density)	564	$g_{DW}\ L_{Cell}^{-1}$	-	[4]	564
	D (Dilution rate)	0 for batch culture<br><br>See main text for continuous culture	h^{-1}	-	-	

References

1. Kotte O, Zaugg JB, Heinemann M: **Bacterial adaptation through distributed sensing of metabolic fluxes.** *Mol Syst Biol* 2010, **6:**355.

2. Matsuoka Y, Shimizu K: **Catabolite regulation analysis of *Escherichia coli* for acetate overflow mechanism and co-consumption of multiple sugars based on systems biology approach using computer simulation.** *J Biotechnol* 2013, **168:**155-173.

3. Altintas MM, Eddy CK, Zhang M, McMillan JD, Kompala DS: **Kinetic modeling to optimize pentose fermentation in *Zymomonas mobilis*.** *Biotechnol Bioeng* 2006, **94:**273-295.

4. Chassagnole C, Noisommit-Rizzi N, Schmid JW, Mauch K, Reuss M: **Dynamic modeling of the central carbon metabolism of *Escherichia coli*.** *Biotechnol Bioeng* 2002, **79:**53-73.

5. Hoefnagel MH, Starrenburg MJ, Martens DE, Hugenholtz J, Kleerebezem M, Van S, II, Bongers R, Westerhoff HV, Snoep JL: **Metabolic engineering of lactic acid bacteria, the combined approach: kinetic modelling, metabolic control and experimental analysis.** *Microbiology* 2002, **148:**1003-1013.

6. Wright BE, Butler MH, Albe KR: **Systems analysis of the tricarboxylic acid cycle in *Dictyostelium discoideum*. I. The basis for model construction.** *J Biol Chem* 1992, **267:**3101-3105.

7. Yang C, Hua Q, Baba T, Mori H, Shimizu K: **Analysis of *Escherichia coli* anaplerotic metabolism and its regulation mechanisms from the metabolic responses to altered dilution rates and phosphoenolpyruvate carboxykinase knockout.** *Biotechnol Bioeng* 2003, **84:**129-144.

8. Peskov K, Mogilevskaya E, Demin O: **Kinetic modelling of central carbon metabolism in *Escherichia coli*.** *FEBS J* 2012, **279:**3374-3385.

9. Kadir TA, Mannan AA, Kierzek AM, McFadden J, Shimizu K: **Modeling and simulation of the main metabolism in *Escherichia coli* and its several single-gene knockout mutants with experimental verification.** *Microb Cell Fact* 2010, **9:**88.

10. Toya Y, Ishii N, Nakahigashi K, Hirasawa T, Soga T, Tomita M, Shimizu K: **13C-metabolic flux analysis for batch culture of *Escherichia coli* and its *Pyk* and *Pgi* gene knockout mutants based on mass isotopomer distribution of intracellular metabolites.** *Biotechnol Prog* 2010, **26:**975-992.

11. Bennett BD, Kimball EH, Gao M, Osterhout R, Van Dien SJ, Rabinowitz JD: **Absolute metabolite concentrations and implied enzyme active site occupancy in *Escherichia coli*.** *Nat Chem Biol* 2009, **5:**593-599.

12. Buchholz A, Hurlebaus J, Wandrey C, Takors R: **Metabolomics: quantification of intracellular metabolite dynamics.** *Biomol Eng* 2002, **19:**5-15.

13. Usuda Y, Nishio Y, Iwatani S, Van Dien SJ, Imaizumi A, Shimbo K, Kageyama N, Iwahata D, Miyano H, Matsui K: **Dynamic modeling of *Escherichia coli* metabolic and regulatory systems for amino-acid production.** *J Biotechnol* 2010, **147:**17-30.

14. Kotlarz D, Buc H: **Phosphofructokinases from *Escherichia coli*.** *Methods Enzymol* 1982, **90 Pt E:**60-70.

15. Babul J, Guixe V: **Fructose bisphosphatase from *Escherichia coli*. Purification and characterization.** *Arch Biochem Biophys* 1983, **225:**944-949.

16. Baldwin SA, Perham RN, Stribling D: **Purification and characterization of the class-II D-fructose 1,6-bisphosphate aldolase from *Escherichia coli* (Crookes' strain).** *Biochem J* 1978, **169:**633-641.

17. Hillman JD: **Mutant analysis of glyceraldehyde 3-phosphate dehydrogenase in *Escherichia coli*.** *Biochem J* 1979, **179:**99-107.

18. Malcovati M, Valentini G: **AMP- and fructose 1,6-bisphosphate-activated pyruvate kinases from *Escherichia coli*.** *Methods Enzymol* 1982, **90 Pt E:**170-179.

19. Berman KM, Cohn M: **Phosphoenolpyruvate synthetase of *Escherichia coli*. Purification, some properties, and the role of divalent metal ions.** *J Biol Chem* 1970, **245:**5309-5318.

20. Visser J, Strating M: **Pyruvate dehydrogenase complex from *Escherichia coli*.** *Methods Enzymol* 1982, **89 Pt D:**391-399.

21. Faloona GR, Srere PA: ***Escherichia coli* citrate synthase. Purification and the effect of potassium on some properties.** *Biochemistry* 1969, **8:**4497-4503.

22. Garnak M, Reeves HC: **Purification and properties of phosphorylated isocitrate dehydrogenase of *Escherichia coli*.** *J Biol Chem* 1979, **254:**7915-7920.

23. Reed LJ, Mukherjee BB: **α- ketoglutarate dehydrogenase complex from *E.coli*.** *Methods Enzymol* 1969, **13:** 55-61.

24. Kita K, Vibat CR, Meinhardt S, Guest JR, Gennis RB: **One-step purification from *Escherichia coli* of complex II (succinate: ubiquinone oxidoreductase) associated with succinate-reducible cytochrome b556.** *J Biol Chem* 1989, **264:**2672-2677.

25. Flint DH: **Initial kinetic and mechanistic characterization of *Escherichia coli* fumarase A.** *Arch Biochem Biophys* 1994, **311:**509-516.

26. Sutherland P, McAlister-Henn L: **Isolation and expression of the *Escherichia coli* gene encoding malate dehydrogenase.** *J Bacteriol* 1985, **163:**1074-1079.

27. Iwakura M, Hattori J, Arita Y, Tokushige M, Katsuki H: **Studies on regulatory functions of malic enzymes. VI. Purification and molecular properties of NADP-linked malic enzyme from *Escherichia coli* W.** *J Biochem* 1979, **85:**1355-1365.

28. Goldie AH, Sanwal BD: **Allosteric control by calcium and mechanism of desensitization of phosphoenolpyruvate carboxykinase of *Escherichia coli*.** *J Biol Chem* 1980, **255:**1399-1405.

29. Wohl RC, Markus G: **Phosphoenolpyruvate carboxylase of *Escherichia coli*. Purification and some properties.** *J Biol Chem* 1972, **247:**5785-5792.

30. MacKintosh C, Nimmo HG: **Purification and regulatory properties of isocitrate lyase from *Escherichia coli* ML308.** *Biochem J* 1988, **250:**25-31.

31. Chung T, Resnik E, Stueland C, LaPorte DC: **Relative expression of the products of glyoxylate bypass operon: contributions of transcription and translation.** *J Bacteriol* 1993, **175:**4572-4575.

32. Wanner BL: **Phosphorus assimilation and control of the phosphate regulon. In *Escherichia coli* and *Salmonella*: cellular and molecular biology.(Neidhardt EA, ed.)** *ASM Press, Washington,DC* 1996**:**1357–1381.

33. Scholte BJ, Schuitema AR, Postma PW: **Isolation of IIIGlc of the phosphoenolpyruvate-dependent glucose phosphotransferase system of *Salmonella typhimurium*.** *J Bacteriol* 1981, **148:**257-264.

34. Ishizuka H, Hanamura A, Kunimura T, Aiba H: **A lowered concentration of cAMP receptor protein caused by glucose is an important determinant for catabolite repression in *Escherichia coli*.** *Mol Microbiol* 1993, **10:**341-350.

35. Quail MA, Guest JR: **Purification, characterization and mode of action of PdhR, the transcriptional repressor of the pdhR-aceEF-lpd operon of *Escherichia coli*.** *Mol Microbiol* 1995, **15:**519-529.

36. Volkmer B, Heinemann M: **Condition-dependent cell volume and concentration of *Escherichia coli* to facilitate data conversion for systems biology modeling.** *PLoS One* 2011, **6:**e23126.

37. Scopes RK: **6-Phosphogluconolactonase from *Zymomonas mobilis*: An enzyme of high catalytic efficiency.** *FEBS Letters* 1985, **193:**185-188.

38. Pettersson G: **What metabolite levels may be evolutionarily reached in the glycolytic pathway?** *Eur J Biochem* 1990, **194:**141-146.

39. Takama M, Nosoh Y: **Purification and some properties of 6-phosphoglucose isomerase from *Bacillus caldotenax*.** *J Biochem* 1980, **87:**1821-1827.

40. Dykhuizen DE, Hartl DL: **Functional effects of PGI allozymes in *Escherichia coli*.** *Genetics* 1983, **105:**1-18.

41. Schreyer R, Bock A: **Phosphoglucose isomerase from *Escherischia coli* K 10: purification, properties and formation under aerobic and anaerobic condition.** *Arch Microbiol* 1980, **127:**289-298.

42. Rizzi M, Baltes M, Theobald U, Reuss M: **In vivo analysis of metabolic dynamics in *Saccharomyces cerevisiae*: II. Mathematical model.** *Biotechnol Bioeng* 1997, **55:**592-608.

43. Deville-Bonne D, Laine R, Garel JR: **Substrate antagonism in the kinetic mechanism of *E. coli* phosphofructokinase-1.** *FEBS Lett* 1991, **290:**173-176.

44. Diaz Ricci JC: **Influence of Phosphoenolpyruvate on the Dynamic Behaviour of Phosphofructokinase of *Escherichia coli*.** *Journal of Theoretical Biology* 1996, **178:**145-150.

45. Babul J, Clifton D, Kretschmer M, Fraenkel DG: **Glucose metabolism in *Escherichia coli* and the effect of increased amount of aldolase.** *Biochemistry* 1993, **32:**4685-4692.

46. Bakker BM, Michels PA, Opperdoes FR, Westerhoff HV: **Glycolysis in bloodstream form Trypanosoma brucei can be understood in terms of the kinetics of the glycolytic enzymes.** *J Biol Chem* 1997, **272:**3207-3215.

47. Boiteux A, Markus M, Plesser T, Hess B, Malcovati M: **Analysis of progress curves. Interaction of pyruvate kinase from *Escherichia coli* with fructose 1,6-bisphosphate and calcium ions.** *Biochem J* 1983, **211:**631-640.

48. Snoep JL, Teixeira de Mattos MJ, Starrenburg MJ, Hugenholtz J: **Isolation, characterization, and physiological role of the pyruvate dehydrogenase complex and alpha-acetolactate synthase of Lactococcus lactis subsp. lactis bv. diacetylactis.** *J Bacteriol* 1992, **174:**4838-4841.

49. Snoep JL, Westphal AH, Benen JA, Teixeira de Mattos MJ, Neijssel OM, de Kok A: **Isolation and characterisation of the pyruvate dehydrogenase complex of anaerobically grown *Enterococcus faecalis* NCTC 775.** *Eur J Biochem* 1992, **203:**245-250.

50. Bresters TW, de Kok A, Veeger C: **The pyruvate-dehydrogenase complex from *Azotobacter vinelandii*. 2. Regulation of the activity.** *Eur J Biochem* 1975, **59:**347-353.

51. Abbe K, Takahashi S, Yamada T: **Involvement of oxygen-sensitive pyruvate formate-lyase in mixed-acid fermentation by Streptococcus mutans under strictly anaerobic conditions.** *J Bacteriol* 1982, **152:**175-182.

52. Thauer RK, Jungermann K, Decker K: **Energy conservation in chemotrophic anaerobic bacteria.** *Bacteriol Rev* 1977, **41:**100-180.

53. Fox DK, Roseman S: **Isolation and characterization of homogeneous acetate kinase from *Salmonella typhimurium* and *Escherichia coli*.** *J Biol Chem* 1986, **261:**13487-13497.

54. Heckert LL, Butler MH, Reimers JM, Albe KR, Wright BE: **Purification and characterization of the 2-oxoglutarate dehydrogenase complex from *Dictyostelium discoideum*.** *J Gen Microbiol* 1989, **135:**155-161.

55. Butler MH, Mell GP, Wright BE: **The pyruvate dehydrogenase complex in *Dictyostelium discoideum*.** *Curr Top Cell Regul* 1985, **26:**337-346.

56. Emyanitoff RG, Kelly PJ: **Kinetic characterization of mitochondrial malate dehydrogenase from *Dictyostelium discoideum*.** *J Gen Microbiol* 1982, **128:**1767-1771.

57. Krebs A, Bridger WA: **The kinetic properties of phosphoenolpyruvate carboxykinase of *Escherichia coli*.** *Can J Biochem* 1980, **58:**309-318.

58. Sanwal BD: **Regulatory mechanisms involving nicotinamide adenine nucleotides as allosteric effectors. 3. Control of glucose 6-phosphate dehydrogenase.** *J Biol Chem* 1970, **245:**1626-1631.

59. Miclet E, Stoven V, Michels PA, Opperdoes FR, Lallemand JY, Duffieux F: **NMR spectroscopic analysis of the first two steps of the pentose-phosphate pathway elucidates the role of 6-phosphogluconolactonase.** *J Biol Chem* 2001, **276:**34840-34846.

60. Clarke JL, Mason PJ: **Murine hexose-6-phosphate dehydrogenase: a bifunctional enzyme with broad substrate specificity and 6-phosphogluconolactonase activity.** *Arch Biochem Biophys* 2003, **415:**229-234.

61. Wood WA: **6-Phosphoglucponic and related dehydrases.** *The Enzymes3rd Ed* 1971, **5**.

62. Cheriyan M, Toone EJ, Fierke CA: **Mutagenesis of the phosphate-binding pocket of KDPG aldolase enhances selectivity for hydrophobic substrates.** *Protein Sci* 2007, **16:**2368-2377.

63. de Silva AO, Fraenkel DG: **The 6-phosphogluconate dehydrogenase reaction in *Escherichia coli*.** *J Biol Chem* 1979, **254:**10237-10242.

64. Vaseghi S, Baumeister A, Rizzi M, Reuss M: **In vivo dynamics of the pentose phosphate pathway in *Saccharomyces cerevisiae*.** *Metab Eng* 1999, **1:**128-140.

65. Bremer H, Dennis PP: **Modulation of chemical composition and other parameters of the cell by growth rate. In: Neidehardt FC, editor. *Escherichia coli* and *Salmonella* cellular and molecular biology.** *Washington (D C): ASM Press* 1996**:**1553–1569.
